# Transcriptional and epigenetic targets of MEF2C in human microglia contribute to cellular functions related to autism risk and age-related disease

**DOI:** 10.1038/s41590-025-02299-0

**Published:** 2025-10-22

**Authors:** Celina Nguyen, Emily H. Broersma, Anna S. Warden, Cristina Mora, Claudia Z. Han, Zahara Keulen, Nathanael Spann, Jing Wang, Gabriela Ramirez, Samantha Mak, Samantha Trescott, Mohammadparsa Khakpour, Avalon Johnson, Fatir Qureshi, Michael R. La Frano, Kiana Mohajeri, Michael E. Talkowski, Olivia Corradin, Marie-Ève Tremblay, Christopher K. Glass, Nicole G. Coufal

**Affiliations:** 1 Department of Pediatrics, University of California, San Diego, La Jolla, CA USA; 2 Sanford Consortium for Regenerative Medicine, La Jolla, CA USA; 3 Department of Cellular and Molecular Medicine, University of California, San Diego, La Jolla, CA USA; 4 School of Medical Sciences, University of Victoria, Victoria, British Columbia Canada; 5 Whitehead Institute for Biomedical Research, Cambridge, MA USA; 6 Salk Institute for Biological Studies, La Jolla, CA USA; 7 Center for Genomic Medicine, Massachusetts General Hospital, Boston, MA USA; 8 Department of Neurology, Harvard Medical School, Boston, MA USA; 9 Department of Biology, Massachusetts Institute of Technology, Cambridge, MA USA

**Keywords:** Neuroimmunology, Gene regulation in immune cells, Haematopoietic stem cells, Chromatin

## Abstract

*MEF2C* encodes a transcription factor that is critical in nervous system development. Here, to examine disease-associated functions of MEF2C in human microglia, we profiled microglia differentiated from isogenic *MEF2C*-haploinsufficient and *MEF2C*-knockout induced pluripotent stem cell lines. Complementary transcriptomic and functional analyses revealed that loss of MEF2C led to a hyperinflammatory phenotype with broad phagocytic impairment, lipid accumulation, lysosomal dysfunction and elevated basal inflammatory cytokine secretion. Genome-wide profiling of MEF2C-bound sites coupled with the active regulatory landscape enabled inference of its transcriptional functions and potential mechanisms for MEF2C-associated cellular functions. Transcriptomic and epigenetic approaches identified substantial overlap with idiopathic autism datasets, suggesting a broader role of human microglial MEF2C dysregulation in idiopathic autism. In a mouse xenotransplantation model, loss of MEF2C led to morphological, lysosomal and lipid abnormalities in human microglia in vivo. Together, these studies reveal mechanisms by which reduced microglial MEF2C could contribute to the development of neurological diseases.

## Abstract (web summary)

Coufal and colleagues generated microglia from human iPS cells to examine mechanistic roles of the transcription factor MEF2C and how these roles might relate to the autism phenotype seen following the loss of MEF2C in human microglia.

## Main

Microglia are the tissue-resident macrophages of the brain, with a distinct ontogeny and gene expression pattern compared to other cells of the myeloid lineage^1^. Microglia contribute to classic macrophage activities, such as phagocytosis and initiation of pathogen-associated inflammatory responses, as well as crucial brain-specific processes including neurogenesis^2,3^, plasticity and learning^4,5^, elimination of apoptotic neurons^6,7^ and modulation of synaptic networks^8,9^. Dysregulation of microglia is increasingly recognized in the pathogenesis of neurodevelopmental, neuropsychiatric and neuroinflammatory disease^10,11^. These ‘microgliopathies’ include microglia-specific single-gene mutations and broad groups of neurodegenerative and neuroinflammatory diseases lacking specific genetic diagnoses. Neuroinflammation has been implicated in the pathogenesis of autism spectrum disorder (ASD), with increased levels of inflammatory cytokines and other markers of inflammation described in postmortem brain samples and cerebrospinal fluid of individuals with ASD^12,13^.

Myocyte enhancer factor 2C (encoded by *MEF2C*) is a transcription factor expressed in neurons and microglia, with known roles in neural crest development and neuronal migration^14,15^. Changes in *MEF2C* expression have been noted in disease contexts and with inflammatory perturbations^16,17^. *MEF2C* downregulation has been associated with the deleterious effects of aging and Alzheimer’s disease (AD)^17,18^, and genome-wide association studies have linked *MEF2C* to late-onset AD^19,20^. Additionally, MEF2C haploinsufficiency syndrome (MHS) results in severe developmental delay and intractable epilepsy^21,22^, implying that *MEF2C* may be broadly involved in ASD and other neurodevelopmental disorders. Together, these data suggest a prominent role for *MEF2C* in diverse neurodevelopmental, neuropsychiatric and neurodegenerative disorders.

The DNA-binding motif for the MEF2 family of transcription factors is highly conserved and enriched in microglia-specific enhancers across species^23–26^, with MEF2C predicted as the key family member^27^. Previous studies have delineated the importance of neuronal MEF2C^15,17,28,29^. Yet very few studies have investigated the role of MEF2C in microglia at homeostasis and in developmental or neurological disorders. Moreover, these studies have only used mouse models. A recent study found that global *Mef2c*^+/–^ heterozygous mutant mice display autism-related behaviors and deficits in cortical excitatory synaptic transmission^28^. Dysregulation of microglia-related gene expression suggested a microglial component to developmental phenotypes in these mutant mice. In another mouse study, microglia-specific *Mef2c* knockout (KO) resulted in no transcriptomic differences, but changes in Iba1 protein expression and decreased social preference were reported only after proinflammatory stimulation^16^. These studies have indicated that microglial MEF2C may have a role in normal brain development and in regulating the inflammatory environment of the brain. However, they fall short of describing how MEF2C regulates microglial function and response to environmental cues. Therefore, to understand how perturbations in microglial MEF2C function may underlie neurodevelopmental and neurodegenerative disorders, studies examining the function of MEF2C in human microglia are critically needed.

In this study, we generated MEF2C heterozygous and homozygous loss-of-function lines of induced pluripotent stem (iPS) cells, differentiated these lines into iPS cell-derived microglia in vitro called induced microglia (herein, iMGs) and identified the transcriptional, epigenetic and functional implications of reduced MEF2C expression in human microglia. MEF2C-deficient iMGs exhibited a hyperinflammatory gene program recapitulating signatures seen in neuropsychiatric disorders and in neurodegenerative and aging microglia. Loss of microglial MEF2C also resulted in functional changes, including lysosomal dysregulation with impaired phagocytosis; a proinflammatory phenotype with increased release of nitric oxide, reactive oxygen species (ROS) and proinflammatory cytokines and aging-associated functional deficits. Using chromatin immunoprecipitation both for active histone marks and for MEF2C itself, we identified potential MEF2C-associated transcription factor interactions and overlaid these networks with human-specific genetic disease variants. Finally, to demonstrate that MEF2C-associated phenotypes can be reproduced in vivo, we xenotransplanted MEF2C-deficient iPS cell-derived microglia into a chimeric mouse model. Together, these studies provide key insight into the functions of MEF2C in human microglia in vitro and in vivo and suggest how loss of MEF2C contributes to key aspects of neurodevelopmental disorders and aging-related phenotypes.

## Results

### MEF2C in human microglia across neurodevelopment

**Fig. 1:**
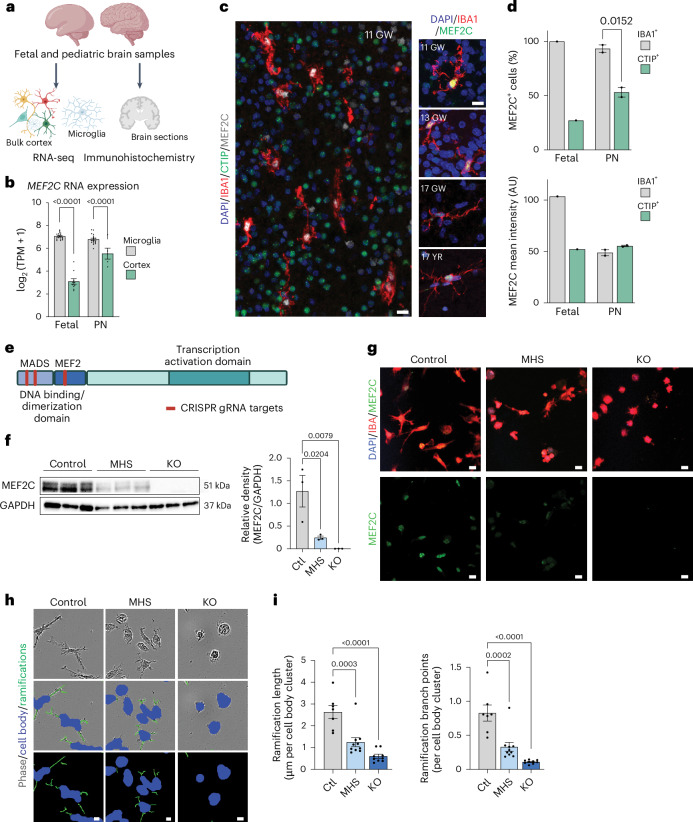
MEF2C is expressed by microglia throughout development, and loss of MEF2C leads to phenotypic changes. **a**, Experimental schematic. Fetal and postnatal brain cortical RNA sequencing data were previously published^27^. In parallel, brain cortical samples were fixed and stained for MEF2C, CTIP and IBA1 across developmental time points. **b**, *MEF2C* RNA levels are greater in isolated microglia than in bulk cortex at both fetal (*n* = 19 microglia and 10 bulk cortex) and postnatal (PN; *n* = 18 microglia and 5 bulk cortex) developmental stages. **c**, Representative confocal images from the cortex of human brain tissue at gestational week (GW) 11 demonstrating colocalization of MEF2C with IBA1^+^ microglia and CTIP^+^ cortical neurons. Left, MEF2C colocalization with IBA^+^ microglia across developmental stages (fetal stages: GW11, 13 and 17; postnatal stage: 17 years (YR)). **d**, Quantification of MEF2C^+^ cells demonstrates more colocalization in IBA1^+^ microglia than in CTIP^+^ cortical neurons in both fetal and postnatal human brain. Fetal microglia also demonstrate higher MEF2C intensity than fetal cortical neurons. Microglia and cortical neurons in postnatal brains demonstrate equivalent levels of MEF2C intensity (*n* = 1 fetal (GW 17) and 2 postnatal (17 and 20 YR) samples; data points represent technical replicates, the mean measurement from five to ten fields per section from three sections per sample); AU, arbitrary units. **e**, Schematic of the CRISPR–Cas9 strategy for MEF2C haploinsufficiency (MHS) and KO human iPS cell lines. **f**, Quantification of western blots showing a dose-dependent reduction in MEF2C protein in MHS and KO iPS cell lines differentiated into microglia (iMGs; *n* = 3 independent iPS cell lines per genotype). **g**, Representative confocal images demonstrating genotype-dependent knockdown of MEF2C protein levels in iMGs (replicated across all lines; *n* = 3 independent iPS cell lines per genotype). **h**, High-throughput image segmentation of iMG morphology for all genotypes. **i**, High-throughput image analysis identifying MEF2C dose-dependent reductions in microglia ramification length and number of branch points per cell body cluster in MHS and KO iMGs (*n* = 7 control (Ctl), 10 MHS and 9 KO iMG biological samples derived from independent iPS cell lines from separate differentiation batches); scale bars, 10 μm in **c**, **g** and **h**. Data in **b**, **d**, **f** and **i** are presented as mean ± s.e.m. Data points in **i** represent the mean measurement for all technical replicates from one independent line in a differentiation batch. Statistical analyses in **b**, **d**, **f** and **i** were performed using one-way analysis of variance (ANOVA) with *P* values adjusted for multiple comparisons by Tukey’s method. Graphics in **a** and **e** were created using BioRender.com. Source data

**Extended Data Fig. 1:**
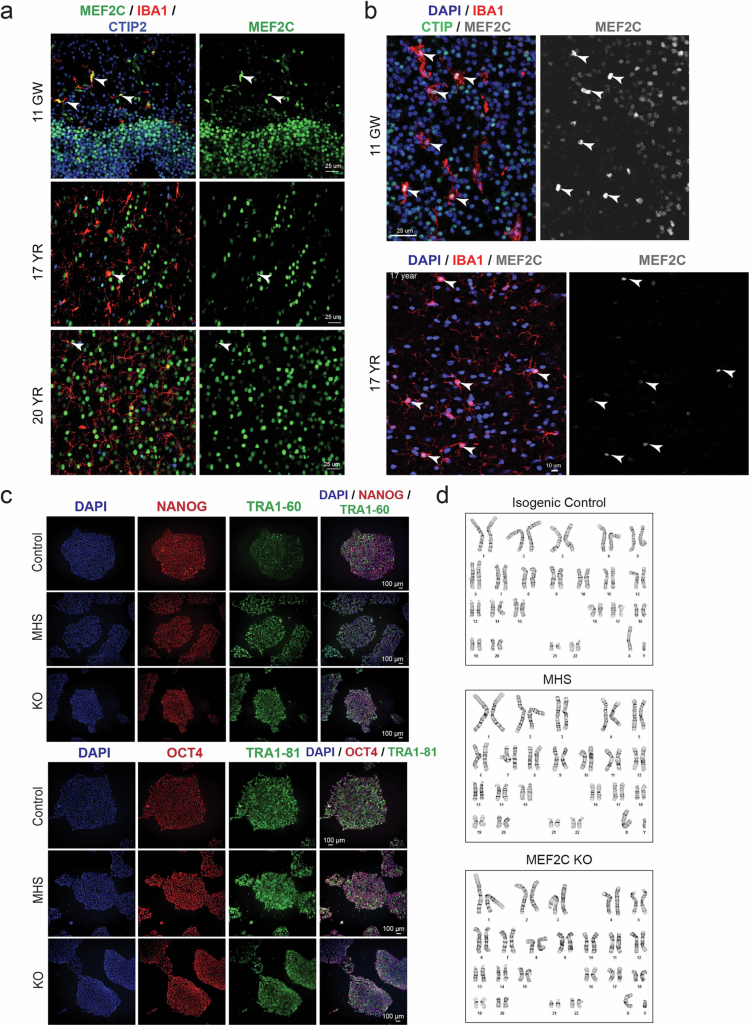
MEF2C expression during brain development and pluripotency marker expression in induced pluripotent stem cell lines in model system. **a**. Microglia demonstrate MEF2C protein expression throughout brain development to adulthood. Arrowheads denote MEF2C signal in IBA+ microglia cell. **b**. 40x magnification showing high levels of MEF2C expression in both fetal and postnatal microglia. Arrowheads denote MEF2C signal in IBA+ microglia cell. **c**. Human induced pluripotent stem cell (iPSC) colonies express canonical pluripotency markers including NANOG, SOX2, TRA-160, and TRA-181. **d**. iPSC lines retain normal 46,XY karyotype after CRISPR-mediated frameshift mutations in *MEF2C* gene. Scale bars = 25 μm in a and b (top); 10 μm in b (bottom); 100 μm in **c**.

MEF2C has been predicted to be a critical regulator of microglial development and function^24,25^. To assess MEF2C in microglia throughout human brain development, we examined microglial MEF2C expression in both primary fetal and postnatal human brain tissue^27^ (Fig. 1a). Analysis of transcriptomic data we previously published^27^ revealed that *MEF2C* RNA was enriched in isolated microglia compared to bulk cerebral cortex at both fetal and postnatal developmental stages (Fig. 1b). In both fetal and postnatal stages, immunostaining revealed that nearly all IBA1^+^ microglia were also positive for MEF2C, whereas a subset of CTIP^+^ neurons co-stained for MEF2C (Fig. 1c,d and Extended Data Fig. 1a,b). MEF2C intensity was higher in fetal IBA1^+^ microglia than in CTIP^+^ neurons, whereas MEF2C intensity was equivalent between IBA^+^ microglia and CTIP^+^ neurons in postnatal brains (Fig. 1d). In keeping with prior transcription factor network analysis identifying MEF2C as integral to the fetal microglial stage^27^, these data suggest that microglial MEF2C may be especially important at early gestational ages.

### CRISPR–Cas9-edited iPS cell-derived model for MEF2C-deficient microglia

**Extended Data Fig. 2:**
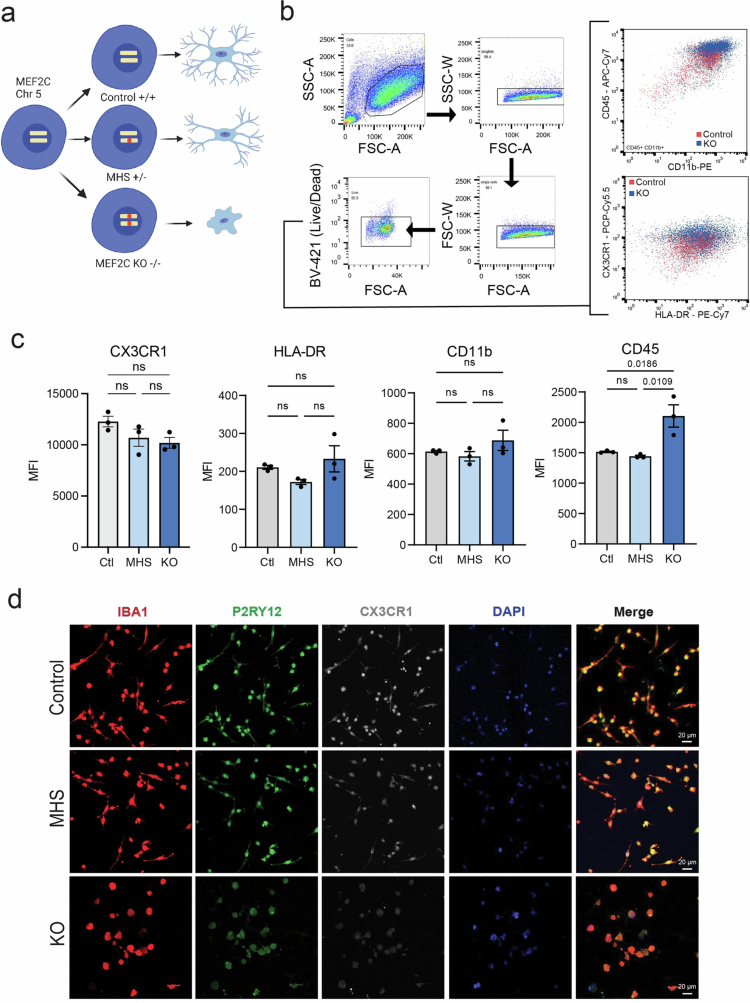
Microglial marker expression in MEF2C-deficient induced pluripotent stem cell-derived microglia *in vitro.* **a**. CRISPR/Cas9-edited human induced pluripotent stem cell (iPSC) model for isogenic control, MEF2C haplo-insufficient (MHS) and knockout (KO) microglia. **b**. Representative flow cytometry data for myeloid and microglia-specific markers, CD45, CD11b, CX3CR1 and HLA-DR in iPSC-derived microglia (iMGs). **c**. Median Fluorescent Index (MFI) of myeloid and microglia-specific markers from iMGs across MEF2C genotypes (*n* = 3 independent iPSC lines per genotype). **d**. Representative images of microglial markers IBA1, P2RY12, and CX3CR1. Scale bars = 20 μm in d. Data in c is presented as mean values ± SEM. Each data point present the mean measurement for all technical replicates from one independent line. Statistical analysis in c was performed using one-way ANOVA tests with P values adjusted for multiple comparisons by Tukey’s method. Graphic in a was created using BioRender.

To further examine the role of MEF2C in microglia development and function, we generated an in vitro iPS cell-based model of MEF2C deficiency using CRISPR–Cas9-mediated gene editing technology to generate frameshift mutations in *MEF2C* in one allele (modeling MHS) or both alleles (KO). In the CRISPR-editing process, three different guide RNAs targeting the MADS or MEF2 domain were used (Fig. 1e) to generate multiple control, MHS and KO isogenic iPS cell lines (Supplementary Table 1 and Extended Data Fig. 1c,d). iPS cell lines from all genotypes were differentiated into iMGs, and loss of MEF2C protein was confirmed (Fig. 1f,g and Extended Data Fig. 2a). There was a dose-dependent decrease in MEF2C protein in MHS compared to control iMGs, suggesting an appropriate model of haploinsufficiency (Fig. 1f,g). Notably, loss of MEF2C protein produced profound changes in microglial morphology. We leveraged a high-throughput automated image analysis approach to characterize microglia ramifications, identifying that loss of MEF2C resulted in decreased length and complexity of ramifications in a genotype-dependent manner (Fig. 1h,i). Further, analysis of microglial marker expression identified an increase in CD45 expression with a modest reduction in CX3CR1 expression (Extended Data Fig. 2c,d). Together, the changes in morphology and elevated CD45 expression suggest that MEF2C may be involved in regulating microglial homeostasis.

### MEF2C regulates inflammatory and disease-associated genes

**Fig. 2:**
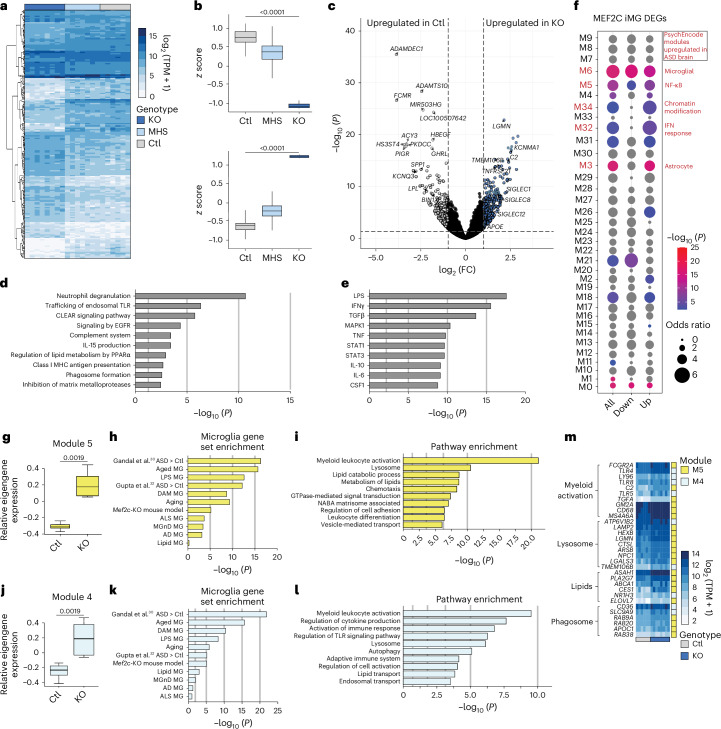
Loss of MEF2C results in transcriptional changes in inflammatory, developmental and neurodegenerative pathways. **a**, Heat map of DEGs in *MEF2C* control, MHS and KO iMGs (*n* = 6 control, 7 MHS and 8 KO iMG biological samples derived from independent iPS cell lines from separate differentiation batches). **b**, *z* Scores of all downregulated (top) or upregulated (bottom) genes between control and KO iMGs demonstrating that MHS iMGs express intermediate levels. Top, control (minimum: 0.311, maximum: 1.155, center: 0.768, 25th percentile: 0.644, 75th percentile: 0.874), MHS (minimum: –0.145, maximum: 0.862, center: 0.363, 25th percentile: 0.217, 75th percentile: 0.508), KO (minimum: –1.155, maximum: –1.014, center: –1.130, 25th percentile: –1.146, 75th percentile: –1.091). Bottom, control (minimum: –1.074, maximum: –0.387, center: –0.736, 25th percentile: –0.825, 75th percentile: –0.647), MHS (minimum: –0.817, maximum: 0.025, center: –0.403, 25th percentile: –0.505, 75th percentile: –0.287), KO (minimum: 1.057, maximum: 1.155, center: 1.136, 25th percentile: 1.111, 75th percentile: 1.149). **c**, Volcano plot of DEGs between control and KO iMGs. Genes significantly upregulated in control microglia are defined by DESeq2 as an FDR of <0.05 and log_2_ (FC) of <–1 and genes significantly upregulated in KO microglia as an FDR of <0.05 and log_2_ (FC) of >1. **d**, IPA for pathway enrichment of DEGs upregulated in KO compared to control iMGs showing strong changes in immune signaling, phagosome formation, autophagy and production of NO/ROS species. **e**, IPA results for upstream regulators of gene expression suggesting that neuroimmune-induced changes in KO iMGs may be regulated by LPS, TNF or IFNγ. **f**, PsychEncode gene module overlap with DEGs between control and KO iMGs demonstrating strong overlap with the neuropsychiatric signature of ASD, BD and SCZ. Inflammatory modules identified by Gandal et al.^30^ are indicated in red text. **g**, WGCNA showing a statistical upregulation of Module 5 in KO iMGs. Control (minimum: –0.368, maximum: –0.241, center: –0.307, 25th percentile: –0.354, 75th percentile: –0.280) and KO (minimum: 0.089, maximum: 0.405, center: 0.193, 25th percentile: 0.091, 75th percentile: 0.307). **h**, Module 5 has strong gene set overlap with ASD signatures, aging microglia, LPS-activated microglia and AD microglia; ALS, amyotrophic lateral sclerosis; DAM, damage-associated microglia; MGnD, neurodegenerative microglia. **i**, Module 5 is enriched in pathways involved in lysosome activity, lipid metabolism, chemotaxis and other homeostatic immune functions in microglia. **j**, WGCNA showing statistical upregulation of Module 4 in KO iMGs. Control (minimum: –0.361, maximum: –0.155, center: –0.260, 25th percentile: –0.321, 75th percentile: –0.170) and KO (minimum: 0.026, maximum: 0.446, center: 0.155, 25th percentile: –0.004, 75th percentile: 0.402). **k**, Module 4 has strong gene set overlap with ASD signatures, aging microglia, LPS-activated microglia and AD microglia. **l**, Module 4 is enriched in pathways involved in the adaptive immune response, cytokine production, autophagy and lipid transport. **m**, Heat map of DEGs between control and KO iMGs by pathway of action (FDR < 0.05, FC < 1.5). Statistical analysis in **b** was performed using a one-way ANOVA with *P* values adjusted for multiple comparisons by Tukey’s method. *P* values for **d**–**f**, **h**–**k** and **l** were determined using hypergeometric tests. Source data

**Extended Data Fig. 3:**
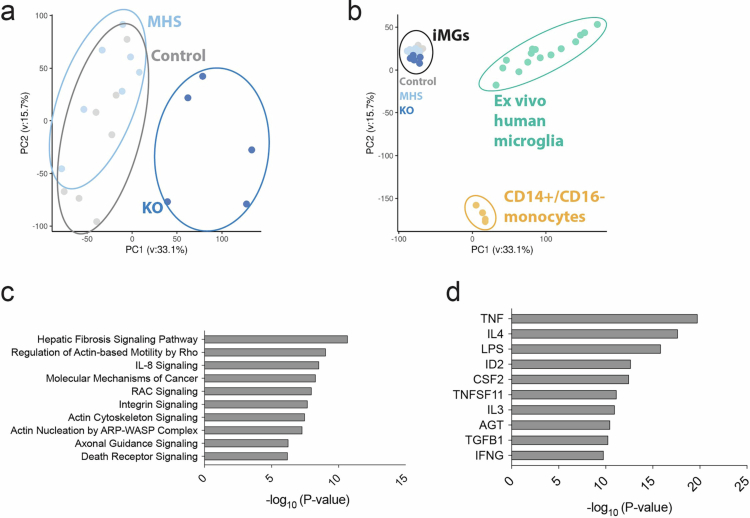
Microglia with complete loss of MEF2C are transcriptomically unique but retain microglia cell identity. **a**. Principal component analysis of whole transcriptome demonstrates similarities between Control (Ctl) and MHS iMGs while KO iMGs cluster separately indicating that complete loss of MEF2C induces a diverging transcriptomic signature (*n* = 7 Ctl, 7 MHS and 5 KO iMG biological samples derived from independent iPSC lines from separate differentiation batches). **b**. Principal component analysis of whole transcriptome demonstrates ultimate similarities between Ctl, MHS and KO iMGs which cluster closer to primary human microglia than other myeloid cells, like CD14±CD16^−^ monocytes (*n* = 7 Ctl, 7 MHS and 5 KO iMG biological samples derived from independent iPSC lines from separate differentiation batches, 4 CD14±CD16^−^ human monocyte samples from different donors, and 15 *ex vivo* microglia samples from Gosselin et al, 2017). **c**. Ingenuity Pathway Analysis (IPA) enrichment for differentially expressed genes downregulated in KO compared to Ctl iMGs. **d**. IPA upstream regulator analysis for differentially expressed genes downregulated in KO compared to Ctl iMGs. P values for c and d were determined using hypergeometric tests. Source data

To investigate the effect of MEF2C dosage on overall gene expression, we performed RNA sequencing (RNA-seq) on iMGs across all three genotypes. We first queried if loss of MEF2C impacted microglial fate. Principal component analysis demonstrated that MHS and KO iMGs clustered with control iMGs near primary ex vivo microglia^24^, but not near other monocytes (Extended Data Fig. 3a,b). For differential gene expression analysis, we used a linear model to account for all group comparisons and uncover potential patterns of differentially expressed genes (DEGs; Fig. 2a). There were no significant DEGs between the control and MHS groups, although there was a tendency (0.05 ≤ *P* ≤ 0.1) toward two distinct patterns of DEGs: downregulation of genes related to cell adhesion, actin cytoskeleton organization and synapse organization as well as upregulation of genes enriched in lysosome function, lipid metabolism and immune activation (Fig. 2b).

We observed strong and significant changes in gene expression between control and *MEF2C*-KO iMGs (Fig. 2c) and therefore focused on comparing the control and KO groups. Differential expression analysis revealed 964 up- and 606 downregulated genes (FDR < 0.05, fold change (FC) > 1.5) in *MEF2C-*KO iMGs (Fig. 2c). Upregulated DEGs were enriched in similar processes as MHS microglia: neuroimmune reactivity, phagosome function, lysosome function and lipid metabolism (Fig. 2d). In addition, downregulated genes were enriched in cell adhesion, synapse organization and lymphocyte differentiation (Extended Data Fig. 3c), suggesting coordinated changes with loss of MEF2C. Moreover, loss of MEF2C led to a downregulation of core microglia genes (for example, *TMEM119* and *CX3CR1*), suggesting a loss of homeostatic control. To predict potential upstream regulators of these coordinated changes in gene expression, we leveraged the upstream regulator analysis tool in Ingenuity Pathway Analysis (IPA). Lipopolysaccharide (LPS) and TNF were predicted to be upstream regulators in *MEF2C*-KO microglia for both up- and downregulated genes (Fig. 2e and Extended Data Fig. 3d), suggesting changes in neuroimmune signaling with loss of MEF2C.

### MEF2C deficiency recapitulates neuropsychiatric transcriptomic signatures

MEF2C is broadly implicated in neuropsychiatric disorders^18^. Therefore, we queried how much our microglial MEF2C model replicates the neuropsychiatric brain transcriptomic signature from the PsychEncode consortium, which includes whole-brain RNA-seq data from individuals diagnosed with ASD, bipolar disorder (BD) and schizophrenia (SCZ)^30^. Comparisons between previously published coexpression network analyses from Gandal and colleagues^30^ and our analysis revealed significant overlap in key modules from PsychEncode, including the microglia-specific module (gene Module 6; Fig. 2f). Specifically, DEGs upregulated in *MEF2C*-KO iMGs compared to control iMGs significantly overlapped with the PsychEncode neuroimmune modules, such as gene Module 5 (NF-κB), gene Module 32 (interferon (IFN) response) and gene Module 34 (chromatin-modifying enzymes; Fig. 2f). These modules were also upregulated in brains from individuals with idiopathic ASD^30^. By contrast, DEGs downregulated in *MEF2C*-KO iMGs overlapped with gene Module 21 (early response genes; Fig. 2f), which are also downregulated in SCZ and BD. Together, these findings suggest that our iMG model of MEF2C loss may capture critical phenotypes of neuropsychiatric and neurodevelopmental disorders such as ASD.

### Network analysis identifies neuropsychiatric-associated gene modules

**Extended Data Fig. 4:**
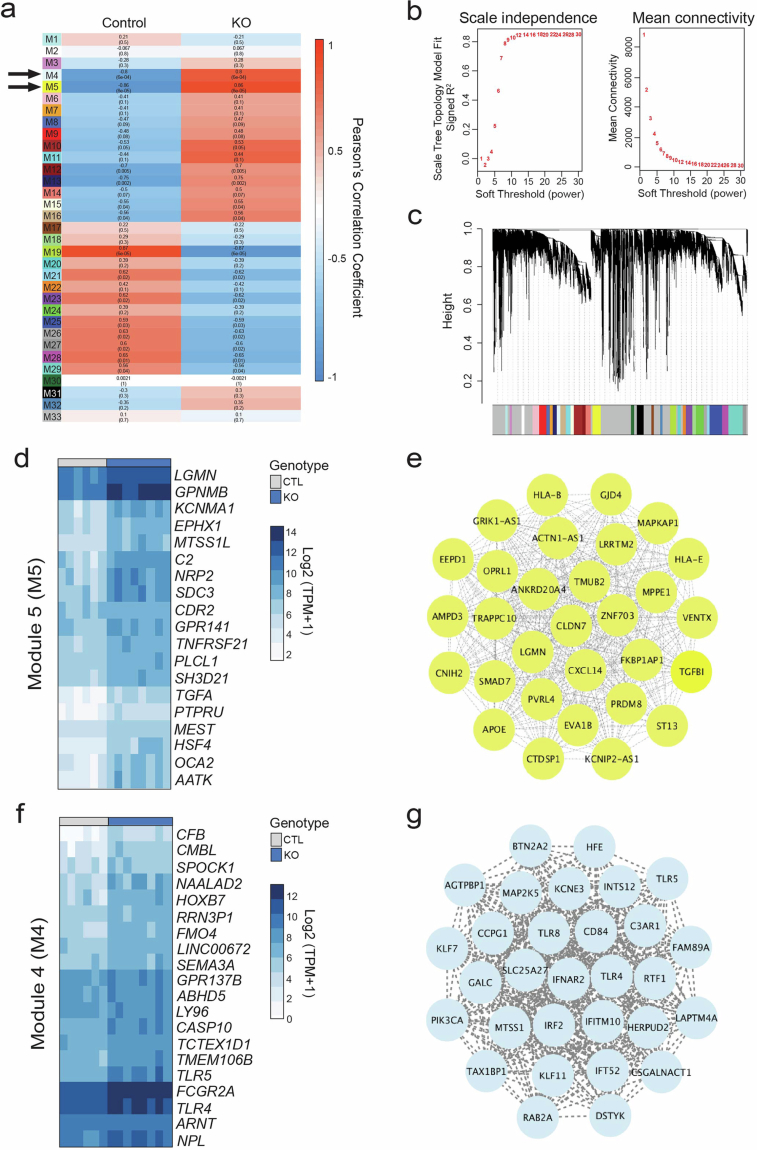
Weighted Gene Correlation Network Analysis of Control and KO RNA-seq data. **a**. Heatmap of Weighted Gene Correlation Network (WGCNA) module-trait relationships for Control (Ctl, left column) and KO (right column) iMG gene expression. Arrows indicate modules that are highly positively correlated with loss of MEF2C. **b**. Scale-independence and mean connectivity of the network in different soft-threshold powers. The left panel displays the correlation of soft threshold with scale-free fit index. The right panel displays the influence of soft-threshold power on mean connectivity. **c**. Dendrogram for WGCNA modules. **d**. Heatmap of expression in Ctl and KO iMGs genes enriched in Module 5. **e**. Module 5 hub genes. **f**. Heatmap of expression in Ctl and KO iMGs genes enriched in Module 4. **g**. Module 4 hub genes. Source data

To further identify molecular targets and develop network-based hypotheses for testing MEF2C function, we next performed a systems-level analysis of *MEF2C* gene expression using Weighted Gene Correlation Network Analysis^31^ (WGCNA; Extended Data Fig. 4a–c). Two modules were highly positively correlated with loss of MEF2C.

The first module, Module 5 (yellow; *P* = 8 × 10^−5^, *r* = 0.86), comprised 576 unique genes (Fig. 2g). Among the genes with a high degree of connectivity (that is, hub genes), the highest-ranking genes regulate key microglia immune pathways, such as *SMAD7* (IFN/transforming growth factor-β (TGFβ) signaling), *HLA-B* (antigen presentation) and *CXCL14* (cytokine signaling; Extended Data Fig. 4d,e). This module is highly enriched in inflammatory genes associated with ASD^30,32^, senescence and aged microglia^33^ (Fig. 2h). Gene Ontology identified pathways related to lysosome function, metabolism of lipids and immune reactivity (Fig. 2i).

The second module, Module 4 (light cyan; *P* = 6 × 10^−4^, *r* = 0.80), comprised 256 unique genes (Fig. 2j). Hub genes for this module demonstrate a clear upregulation of Toll-like receptors (TLRs) and IFN gene expression signaling (*TLR5*, *TLR4*, *IRF2* and *IFNAR1*; Extended Data Fig. 4f,g). This module is highly enriched in gene sets related to ASD, LPS-challenged microglia and disease-associated microglia in AD (Fig. 2k)^30,32,34,35^. Pathway enrichment showed strong enrichment of genes related to cytokine production, TLR signaling/NF-κB activation and lipid transport (Fig. 2l), further supporting our observation that loss of MEF2C produces a hyperinflammatory phenotype with similarities to ASD-related, aging and neurodegenerative (that is, AD) microglial states (Fig. 2m). Together, these modules that are correlated with MEF2C dose indicate potential functional phenotypes to investigate, including lysosomal function, cytokine production and lipid metabolism.

### Loss of MEF2C alters lysosome-associated function

**Fig. 3:**
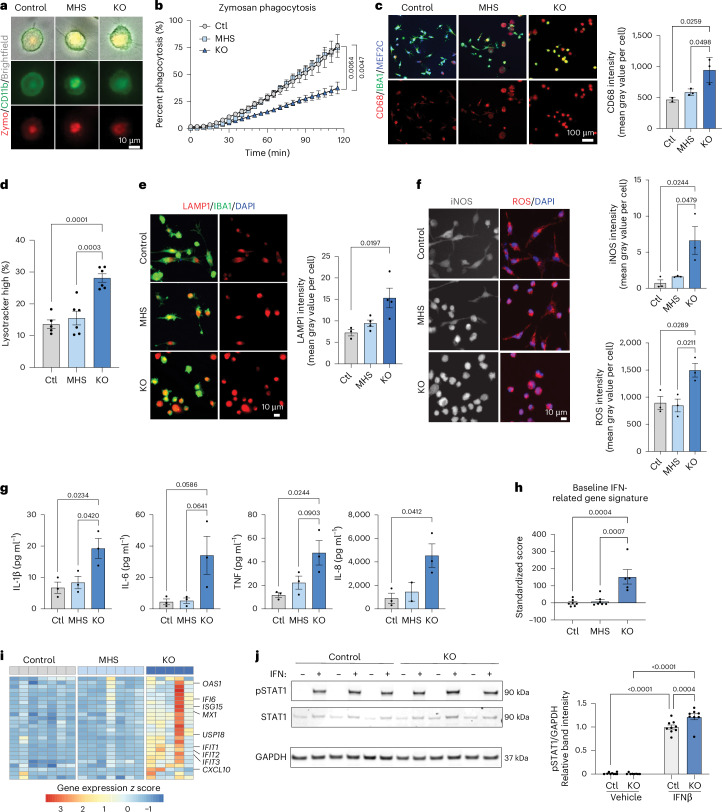
Functional analysis of MEF2C iMGs reveals deficits with loss of MEF2C. **a**, Representative images of phagocytosed zymosan A bioparticles in *MEF2C* control, MHS and KO iMGs. **b**, Quantification of zymosan A phagocytosis demonstrates phagocytosis deficits in KO compared to control and MHS iMGs (*n* = 2 control, 2 MHS and 2 KO iMG biological samples derived from independent iPS cell lines). **c**, Representative confocal images and quantification of lysosomal marker CD68 (*n* = 2 control, 3 MHS and 3 KO iMG biological samples derived from independent iPS cell lines). **d**, Quantification of Lysotracker suggests increased lysosomal mass in KO compared to control and MHS iMGs (*n* = 5 control, 6 MHS and 6 KO iMG biological samples derived from independent iPS cell lines from separate differentiation batches). **e**, Representative confocal images and quantification of the lysosomal marker LAMP1 (*n* = 3 control, 4 MHS and 4 KO iMG biological samples derived from independent iPS cell lines from separate differentiation batches). **f**, Representative confocal images and quantification of iNOS (left, top bar plot) and ROS (right, bottom bar plot; *n* = 3 control, 3 MHS and 3 KO iMG biological samples derived from independent iPS cell lines). **g**, Quantification of cytokines reveals increased cytokine release at baseline in KO compared to control and MHS iMGs (IL-1β, IL-6 and TNF: *n* = 3 control, 3 MHS and 3 KO; IL-8: *n* = 3 control, 2 MHS and 3 KO iMG biological samples derived from independent iPS cell lines). **h**, Standardized scores generated from IFN-related gene expression at homeostasis (*n* = 6 control, 6 MHS and 5 KO iMG biological samples derived from independent iPS cell lines from separate differentiation batches). **i**, Heat map of gene expression *z* scores for the 26 genes used to generate the scores from **h** across all replicates in each group. **j**, Western blot of phosphorylated STAT1 (pSTAT1) levels in *MEF2C* control and KO iMGs after 30 min of exposure to 20 ng ml^−1^ recombinant human IFNβ. pSTAT1 band intensity was normalized to GAPDH and depicted as relative to the average measurement of *MEF2C* control lines with IFNβ stimulation on the same blot (*n* = 9 control and 8 KO iMG biological samples derived from independent iPS cell lines from separate differentiation batches measured in three separate blots); scale bars, 10 μm (**a**, **e** and **f**) and 100 μm (**c**). Data in **c**–**h** and **j** are presented as mean ± s.e.m. Each data point represents the mean measurement for all technical replicates from one independent line in a differentiation batch. Statistical analyses in **c**–**h** were performed by one-way ANOVA, with *P* values adjusted for multiple comparisons using Tukey’s method. *P* values in **j** were determined by two-way ANOVA with a Fisher’s least significant difference test. Source data

**Extended Data Fig. 5:**
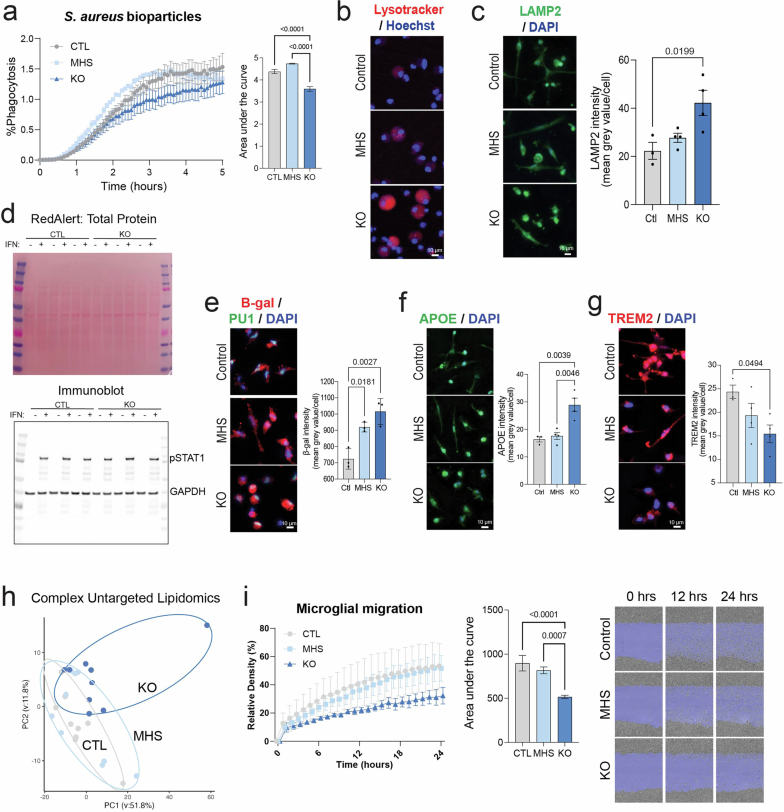
MEF2C KO microglia exhibit phagocytic, lysosomal, and aging-related defects. **a**. Quantification of *Staphylococcus aureus* bioparticle phagocytosis over five hours (left) with Area Under the Curve (AUC) calculation demonstrating phagocytic deficit in KO compared to Control (Ctl) and MHS iMGs (*n* = 3 Ctl, 3 MHS and 3 KO iMG biological samples derived from independent iPSC lines). **b**. Representative images of Lysotracker (*n* = 5 Ctl, 6 MHS, and 6 KO iMG biological samples derived from independent iPSC lines from separate differentiation batches). **c**. Representative images and quantification of lysosomal marker, LAMP2 (*n* = 3 Ctl, 4 MHS, and 4 KO iMG biological samples derived from independent iPSC lines from separate differentiation batches). **d**. One representative pSTAT1 immunoblot analysis with vehicle and IFNβ-treated iMGs (*n* = 3 Ctl and 3 KO iMG biological samples derived from independent iPSC lines treated with vehicle or IFNβ). **e**. Representative images and quantification of Beta-galactosidase (β-gal, *n* = 3 Ctl, 3 MHS, and 3 KO iMG biological samples derived from independent iPSC lines). **f**. Representative images and quantification of neurodegenerative marker, APOE (*n* = 3 Ctl, 4 MHS, and 4 KO iMG biological samples derived from independent iPSC lines from separate differentiation batches). **g**. Representative images and quantification of neurodegenerative marker, TREM2 (*n* = 3 Ctl, 4 MHS, and 4 KO iMG biological samples derived from independent iPSC lines from separate differentiation batches). **h**. Principal component analysis of lipidomic profiles (*n* = 9 Ctl, 9 MHS, and 9 KO iMG biological samples derived from independent iPSC lines from separate differentiation batches). **i**. Quantification of baseline cell migration demonstrates reduced migration in KO compared to Ctl and MHS iMGs. (*n* = 3 Ctl, 3 MHS and 3 KO iMG biological samples derived from independent iPSC lines). Scale bar = 10 μm in b, c, e, f, and g. Data in c, e, f, and g are presented as mean values ± SEM. Each data point represents the mean measurement for all technical replicates from one independent line in a differentiation batch. Statistical analyses in c, e, f, and g were performed using one-way ANOVA tests with P values adjusted for multiple comparisons by Tukey’s method. P values in a and i were determined using one-way ANOVA tests on the area under the curve computed from the related time course measurements with adjustments by Tukey’s method.

We next investigated the functional consequences of MEF2C deficiency to validate predictions from gene expression data. First, we determined that complete loss of MEF2C alters phagocytosis. *MEF2C*-KO iMGs exhibited impaired phagocytosis of zymosan A (Fig. 3a,b) and, to a lesser extent, *Staphylococcus aureus* bioparticles (Extended Data Fig. 5a). Meanwhile, MHS iMGs did not present phagocytic differences from controls.

Microglial lysosomal defects can lead to both impaired phagocytosis and inflammatory cytokine production^36^. We therefore measured protein expression of CD68, a myeloid lysosomal glycoprotein elevated in response to inflammatory stimuli^37^. At baseline, *MEF2C-*KO iMGs exhibited increased CD68 (Fig. 3c), supporting a basal proinflammatory phenotype. *MEF2C-*KO iMGs also exhibited increased lysosomal mass, as measured by LysoTracker (Fig. 3d and Extended Data Fig. 5b), and increased staining for the lysosomal markers LAMP1 (Fig. 3e) and LAMP2 (Extended Data Fig. 5c), indicating alterations in lysosomal function. MHS iMGs exhibited intermediate changes in lysosomal measurements, suggesting a dose-dependent dysregulation of baseline function in MEF2C-deficient iMGs.

### Loss of MEF2C produces a hyperinflammatory phenotype

To assess the impact of MEF2C deficiency on the inflammatory state of microglia, we measured the levels of proinflammatory mediators released in the absence of an inflammatory stimulus. *MEF2C*-KO iMGs had increased inducible nitric oxide synthase (iNOS) and ROS absent of an inflammatory stimulus (Fig. 3f). Additionally, *MEF2C*-KO iMGs released more proinflammatory cytokines (interleukin-1β (IL-1β), IL-6, TNF and IL-8) than MHS and control lines (Fig. 3g). As before, MHS microglia present subtly elevated proinflammatory features.

Microglial MEF2C expression has been negatively correlated with type I IFN signaling after an inflammatory stimulus^16^. To investigate this association in our human model, we generated an IFN-related gene expression score using 26 genes validated to be increased in human IFN-associated disease^38^. In the absence of an inflammatory stimulus, *MEF2C*-KO iMGs express higher levels of IFN-related genes than control and MHS iMGs (Fig. 3h,i), suggesting that KO microglia may be more responsive to IFN stimulation. To assess this primed state, we challenged our iMGs with an acute exposure to human IFNβ and measured phosphorylated STAT1 (pSTAT1) levels. As expected, *MEF2C*-KO microglia showed consistently higher pSTAT1 than control microglia (Fig. 3j and Extended Data Fig. 5d), suggesting a primed response to IFN stimulation. Together, these results indicate that loss of MEF2C leads to dysregulation of the inflammatory response in microglia.

### Loss of MEF2C recapitulates neurodegeneration-related phenotypes

**Fig. 4:**
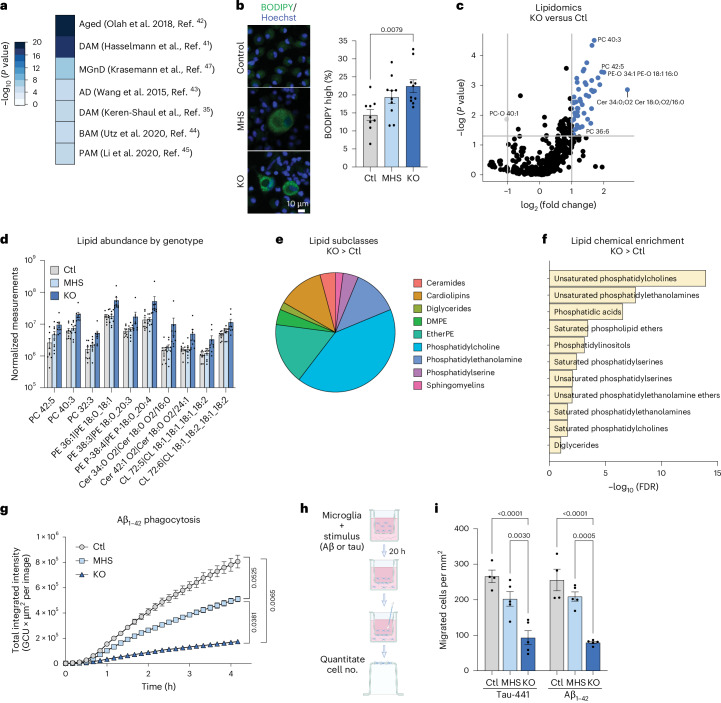
Loss of MEF2C recapitulates a neurodegenerative phenotype. **a**, Overlap of DEGs between control and *MEF2C-*KO iMGs with aged and neurodegenerative microglial datasets; BAM, border-associated macrophages; PAM, proliferative region-associated microglia. **b**, Representative images and quantification of BODIPY in control, MHS and *MEF2C*-KO iMGs suggest increased lipid accumulation in *MEF2C*-KO microglia (*n* = 9 control, 10 MHS and 10 KO iMG biological samples derived from independent iPS cell lines from separate differentiation batches). **c**, Volcano plot of differential analysis of lipids demonstrates that KO iMGs contain more lipids than control iMGs (*n* = 9 control and 9 KO iMG biological samples derived from independent iPS cell lines from separate differentiation batches). Lipids significantly upregulated in control microglia are defined by an FDR of <0.05 and log_2 _(FC) of <–1, and lipids significantly upregulated in KO microglia are defined by an FDR of <0.05 and log_2_ (FC) of >1. **d**, Normalized measurements of select phosphatidylcholines (PC), phosphatidylethanolamines (PE), ceramides (Cer) and cardiolipins across control, MHS and KO iMGs (*n* = 9 control, 9 MHS and 9 KO iMG biological samples derived from independent iPS cell lines from separate differentiation batches). **e**, Distribution of subclasses of significantly increased lipids in KO compared to control iMGs. **f**, Chemical enrichment analysis of increased lipids in KO compared to control iMGs. **g**, Disease-specific phagocytosis of Aβ_1–42_ shows genotype-dependent reductions in phagocytic ability in MHS and KO iMGs (*n* = 2 control, 2 MHS and 2 KO iMG biological samples derived from independent iPS cell lines). GCU, green calibrated unit. **h**, Schematic of a transwell migration assay with disease-specific stimuli. **i**, Quantification of transwell migration assays demonstrating genotype-dependent impaired migration toward the disease-specific stimuli, Tau-441 and Aβ_1–42_ (*n* = 4 control, 5 MHS and 5 KO iMG biological samples derived from independent iPS cell lines from separate differentiation batches); scale bar, 10 μm (**b**). Data in **b**, **d** and **i** are presented as mean values ± s.e.m. Each data point represents the mean measurement for all technical replicates from one independent line in a differentiation batch. Statistical analyses in **b** and **i** were performed by one-way ANOVA with *P* values adjusted for multiple comparisons by Tukey’s method. *P* values from **g** were determined from the final time point measurements using a one-way ANOVA with adjustment for multiple comparisons by Tukey’s method. The graphic in **h** was created with BioRender.com. Source data

MEF2C expression decreases in aged mice^16^, and high MEF2C levels are a predictor of longevity, cognitive potential and resilience to neurodegeneration^39^. Genes dysregulated by *MEF2C* KO overlapped with aging microglia and AD gene sets, presenting an opportunity to assess phenotypes related to aging and neurodegeneration^40–45^ (Fig. 4a). To assess a general connection to microglial aging, we quantified β-galactosidase (β-gal), a common marker of cellular aging^46^, and APOE, which is upregulated in aging and neurodegenerative disease^47^. Both β-gal and APOE were increased in KO iMGs (Extended Data Fig. 5e,f). Additionally, TREM2 expression was decreased (Extended Data Fig. 5g) in *MEF2C*-KO iMGs, providing protein-level validation of gene expression profiling.

Lipid accumulation is a key pathologic feature of microglial aging^48^, and lipid-related processes were notably enriched in the MEF2C transcriptomic analysis (Fig. 2i,l,m). We therefore quantified microglial lipid droplet accumulation and observed increased BODIPY in KO iMGs (Fig. 4b). To identify the lipids specifically affected by loss of MEF2C, we applied unbiased screening of complex lipids by mass spectrometry, measuring 468 lipid species from seven main categories. Multivariate comparison of groups identified that the lipid profile of *MEF2C-*KO iMGs was distinct, whereas MHS iMGs were not different from controls (Extended Data Fig. 5h). Differential analysis identified 48 lipids as elevated in KO iMGs compared to controls, corroborating that *MEF2C*-KO microglia indeed accumulated lipids (Fig. 4c,d). These differentially abundant lipids primarily belonged to the subclasses of phospholipids, phosphatidylethanolamines and phosphatidylcholines (Fig. 4e,f). Phospholipids are the primary components of cell membranes and are remarkably dysregulated in the human brain with age^49^. Notably, *N*-dimethylphosphatidylethanolamines (DMPEs) are also dysregulated in KO iMGs. The DMPE lipids are intermediates in choline production, indicating that loss of MEF2C may contribute to abnormalities in an important neurotransmitter and nutrient in the brain.

To assess microglial function in a neurodegenerative disease-specific context, we measured the phagocytosis and migration capacity of MEF2C-deficient microglia toward disease-relevant stimuli. Although only KO iMGs exhibited reduced phagocytic capacity toward bioparticles (Fig. 3a and Extended Data Fig. 5a), both KO and MHS iMGs phagocytosed fibrillar amyloid-β_1–42_ (Aβ_1–42_) significantly less than controls (Fig. 4g). Moreover, although only KO iMGs exhibited reduced baseline migration capacity (Extended Data Fig. 5i), both KO and MHS iMGs exhibited reduced migration toward disease-specific stimuli, such as Aβ_1–42_ and Tau-441 (Fig. 4h,i). These results indicate that loss of MEF2C is associated with dose-dependent functional deficits pertinent to aging and neurodegenerative disease-associated microglia^35,47,50^.

### MEF2C regulates the active enhancer landscape

**Fig. 5:**
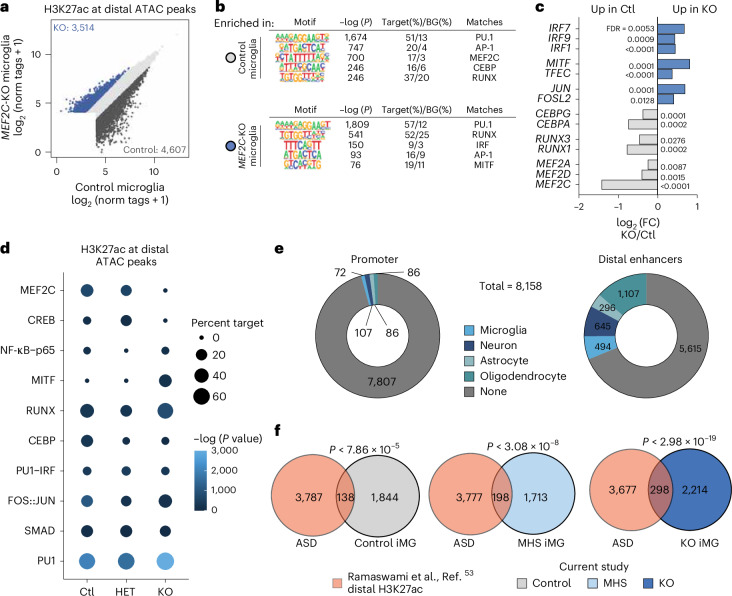
Loss of MEF2C remodels the active enhancer landscape. **a**, Scatter plot of H3K27ac ChIP–seq signal around distal (>1,000 base pairs (bp) from TSS) ATAC–seq peaks in *MEF2C* control and KO iMGs (ATAC–seq: *n* = 3 control and 2 KO; H3K27ac ChIP–seq: *n* = 4 control and 4 KO iMG biological samples derived from independent iPS cell lines from separate differentiation batches). Differentially acetylated regions enriched in control iMGs are defined by DESeq2 as an FDR of <0.05 and log_2_ (FC) of <–1 and enriched in KO iMGs as an FDR of <0.05 and log_2_ (FC) of >1. **b**, De novo motif analysis of differentially acetylated regions enriched in control (top) and KO iMGs (bottom). GC-matched sequences were used as background (BG). **c**, Bar chart showing gene expression of transcription factors whose DNA-binding motifs were identified in **b**. FDR determined by DESeq2. **d**, Bubble plot derived from de novo motif analysis of differentially acetylated regions, delineating transcription factor DNA-binding motif enrichment for all *MEF2C* genotypes (ATAC–seq: *n* = 3 control, 2 MHS and 2 KO; H3K27ac ChIP–seq: *n* = 4 control, 4 MHS and 4 KO iMG biological samples derived from independent iPS cell lines from separate differentiation batches). **e**, Differentially acetylated peaks from Ramaswami et al.^53^ between control and idiopathic autism frontal and cortical brain samples, which were annotated for overlap with cell-type-specific promoter (left) and enhancer (right) peaks from Nott et al.^23^. **f**, Overlaps between genes nearest to differentially acetylated peaks in idiopathic autism compared to control brain tissue (Ramaswami et al.^53^) and genes nearest to H3K27ac-marked distal ATAC peaks in control (left), MHS (middle) and KO iMGs (right). *P* values were determined using hypergeometric tests.

**Extended Data Fig. 6:**
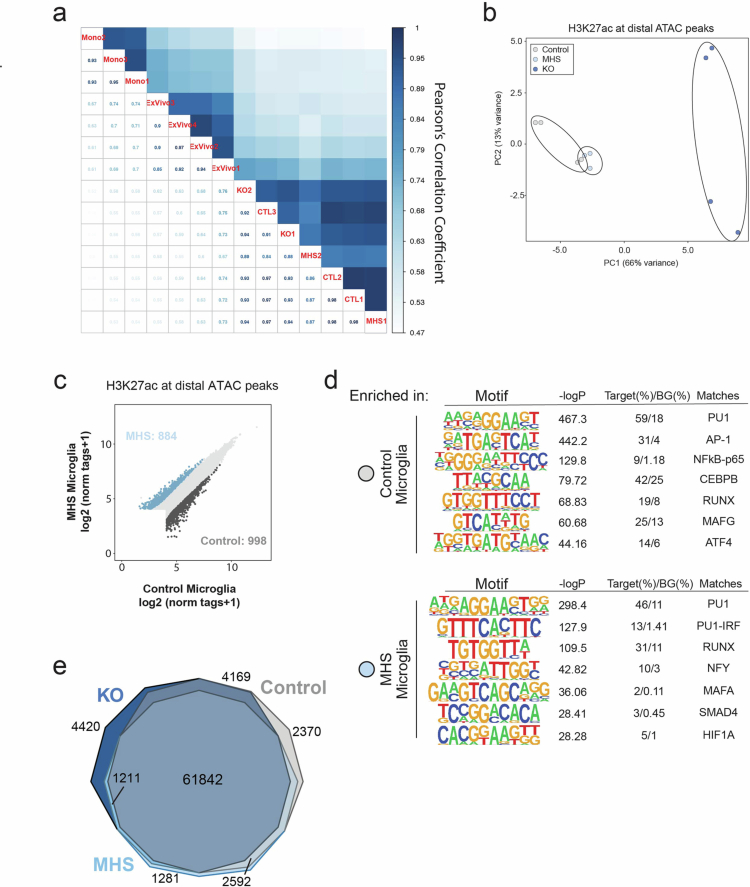
MEF2C microglia exhibits distinct differences in the regulatory landscape. **a**. Correlation plot comparing ATAC-seq peaks from Control (Ctl), MHS and KO iMGs, human primary *ex vivo* microglia, and human primary CD14±CD16^−^ monocytes isolated from umbilical cord blood (*n* = 3 Ctl, 2 MHS and 2 KO iMG biological samples derived from independent iPSC lines from separate differentiation batches, 3 CD14±CD16^−^ human monocyte samples from different donors, and 4 *ex vivo* microglia samples from Gosselin et al, 2017). **b**. Principle component analysis of H3K27ac-marked distal ATAC peaks demonstrates that the active enhancer landscape are similar for Ctl and MHS iMGs, but unique for KO iMGs (ATAC-seq: *n* = 3 Ctl, 2 MHS and 2 KO; H3K27ac ChIP-seq: *n* = 4 Ctl, 3 MHS and 4 KO iMG biological samples derived from independent iPSC lines from separate differentiation batches). **c**. Scatter plot of H3K27ac ChIP-seq signal around distal ( > 1000 bp from TSS) ATAC-seq peaks in Ctl and MHS iMGs (ATAC-seq: *n* = 3 Ctl and 2 MHS; H3K27ac ChIP-seq: *n* = 4 Ctl and 3 MHS iMG biological samples derived from independent iPSC lines from separate differentiation batches). **d**. *De novo* motif analysis of differentially acetylated regions enriched in Control (top) and in MHS iMGs (bottom). GC-matched sequences were used as background. **e**. Chow-Rusky plot demonstrating the overlap of H3K27ac-marked distal ATAC peaks between the three MEF2C genotypes.

Environmentally regulated signals act through a combination of lineage-determining and signal-dependent transcription factors, binding to active distal regulatory regions to initiate cell-type-specific programs^51^. To infer transcription factors that may regulate MEF2C-associated microglial phenotypes, we defined putative enhancers, namely regions of open and active chromatin, through assays for transposase accessible chromatin (ATAC–seq) and chromatin immunoprecipitation with sequencing (ChIP–seq) for histone 3 lysine 27 acetylation (H3K27ac), a mark of active promoters and enhancers^52^. Notably, regions of open chromatin from iMGs in all three groups (control, MHS and KO) correlated more closely to primary ex vivo microglia than to CD14^+^ primary monocytes, indicating retention of the microglial epigenetic signature despite loss of MEF2C (Extended Data Fig. 6a). Principal component analysis of H3K27ac at distal ATAC peaks found that MHS microglia lie between *MEF2C*-KO and control microglia (Extended Data Fig. 6b). In pairwise comparisons, we identified over 8,000 differentially active enhancers (FC > 2, adjusted *P* < 0.05) between control and KO iMGs (Fig. 5a) and more than 1,800 differential enhancers between control and MHS iMGs (Extended Data Fig. 6c). De novo motif analysis of putative microglial enhancers identified loss of MEF2 motifs in KO iMGs (Fig. 5b), but not in MHS (Extended Data Fig. 6d). Notably, we observed enrichment for motifs for IFN regulatory factors (IRFs) and microphthalmia-associated transcription factor (MITF) in active enhancers specific to KO iMGs as well as increased expression of these factors in KO iMGs (Fig. 5c,d). Increased IRF activity may play a role in priming KO iMGs to IFN stimulation, while the MITF/TFE transcription factor family members play significant roles in lysosome function and biogenesis. The changes in active enhancers with loss of MEF2C reveal alterations in transcription factor activity with links to the cellular phenotypes described above.

### Relevance of microglial MEF2C to autism genetics

Previous studies on enhancer activity in frontal and temporal cortical tissue from individuals with idiopathic autism^53^ have not been annotated by their distance from transcription start sites (TSSs) nor by cell-type specificity. Using these published data, we identified distal enhancers with active histone acetylation (H3K27ac) enriched in autism compared to control cortical samples^53^ and mapped these regions to cell-type-specific enhancers, as we have previously described^23^. Complete enhancer overlap between the autism^53^ and brain cell-type-specific^23^ datasets identified that autism-associated enhancers mapped to microglia-, neuron- and oligodendrocyte-specific enhancers relatively equivalently (Fig. 5e). An overlay between the nearest gene for active autism-associated enhancers and active enhancers in each of the three *MEF2C* microglial genotypes identified the greatest overlap with *MEF2C*-KO microglia (Fig. 5f). In line with our transcriptomic analyses, these results further demonstrate how our model of MEF2C loss captures critical aspects of ASD genetics.

### MEF2C is a transcriptional activator in human microglia

**Fig. 6:**
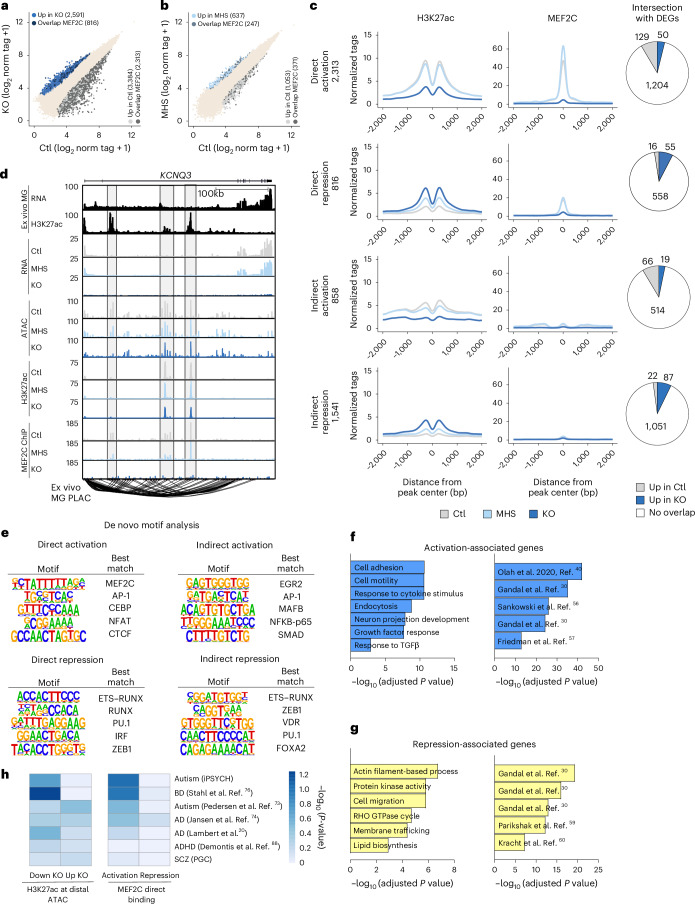
MEF2C acts primarily as an activator in microglia, but repression is more MEF2C dose dependent. **a**, Scatter plot of distal H3K27ac-marked ATAC peaks overlapping with MEF2C binding sites for control and KO iMGs (ATAC–seq: *n* = 3 control and 2 KO; H3K27ac ChIP–seq: *n* = 4 control and 4 KO; MEF2C ChIP–seq: *n* = 2 control and 2 KO iMG biological samples derived from independent iPS cell lines from separate differentiation batches). Differentially acetylated regions enriched in control iMGs are defined by DESeq2 as an FDR of <0.05 and log_2_ (FC) of <–1 and enriched in KO iMGs as an FDR of <0.05 and log_2_ (FC) of >1. **b**, Scatter plot of distal H3K27ac-marked ATAC peaks overlapping with MEF2C binding sites for control and MHS iMGs (ATAC–seq: *n* = 3 control and 2 MHS; H3K27ac ChIP–seq: *n* = 4 control and 4 MHS; MEF2C ChIP–seq: *n* = 2 control and 2 MHS iMG biological samples derived from independent iPS cell lines from separate differentiation batches). Differentially acetylated regions enriched in control iMGs are defined by DESeq2 as an FDR of <0.05 and log_2 _(FC) of <–1 and enriched in MHS iMGs as an FDR of <0.05 and log_2 _(FC) of >1. **c**, Histograms of normalized H3K27ac and MEF2C ChIP–seq tag counts from control, MHS and KO iMGs at distal MEF2C-associated regulatory regions: direct activation (presence of MEF2C and loss of H3K27ac in KO iMGs), direct repression (presence of MEF2C and gain of H3K27ac in KO iMGs), indirect activation (lack of MEF2C and loss of H3K27ac in KO iMGs) and indirect repression (lack of MEF2C and gain of H3K27ac in KO iMGs). **d**, Genome browser tracks of RNA-seq, ATAC–seq, H3K27ac and MEF2C ChIP–seq from control, MHS and KO iMGs with RNA-seq, H3K27ac and PLAC–seq^23,24^ from primary microglia ex vivo at *KCNQ3*, a high-confidence ASD gene. **e**, De novo motif enrichment analysis of MEF2C-associated regulatory regions using total peaks as background. **f**, Gene Ontology (left) and overlap with published gene set enrichment^71^ (right) for MEF2C-associated peaks annotated for transcriptional activation activity. **g**, Gene Ontology (left) and overlap with published gene set enrichment^71^ (right) for MEF2C-associated peaks annotated for transcriptional repression activity. **h**, Heat map of linkage disequilibrium score regression analysis for enrichment of genetic variants associated with the listed conditions^72–77^ displayed as –log_10_ (normalized *P* value) for significance of enrichment in differentially H3K27ac-marked distal enhancers (left) and MEF2C activation and repression-associated enhancers (right); ADHD, attention-deficit/hyperactivity disorder; PGC, Psychiatric Genomics Consortium. *P* values for **f**, **g** and **h** were determined using hypergeometric tests.

**Extended Data Fig. 7:**
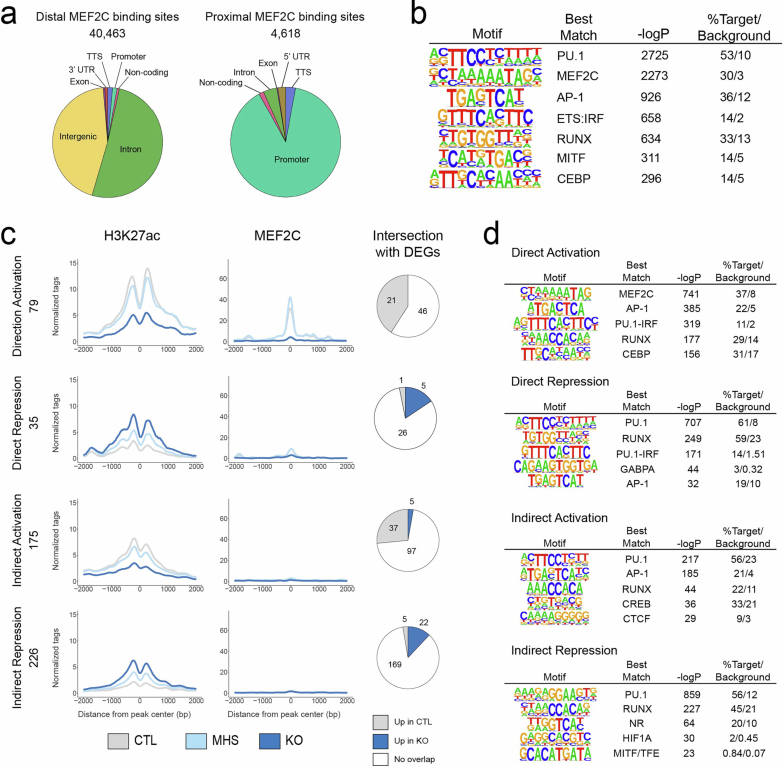
MEF2C acts as a transcriptional activator and repressor at distal and proximal genomic sites in human microglia. **a**. Breakdown of categories of genomic regions for distal ( > 500 bp from TSS) and proximal ( < 500 bp from TSS) sites occupied by MEF2C. **b***. De novo* motif analysis of all MEF2C binding sites. GC-matched sequences were used as background. **c**. Histograms of normalized H3K27ac and MEF2C ChIP-seq tag counts from Ctl, MHS and KO iMGs proximal to promoters, including the amount of overlap of nearby genes with differentially expressed genes between control and MEF2C KO iMGs. **d***. De novo* motif enrichment analysis of MEF2C-associated regulatory regions using total peaks as background (same as Fig. 6e with -log10(p-value) and %target/background). All peaks from the analyzed samples were used as background.

Although MEF2C binding sites have been identified previously in cortical neurons^54^, no dataset exists in microglia. Therefore, to characterize the regulatory function of MEF2C in human microglia, we performed ChIP–seq for MEF2C. We identified 4,708 MEF2C binding sites in control microglia but only 62 sites in *MEF2C-*KO microglia, confirming the specificity of the immunoprecipitation. The majority of MEF2C binding sites (*n* = 4,360) localized to distal regions, namely introns and intergenic regions, whereas a small portion (*n* = 348) resided in regions proximal to the TSS (Extended Data Fig. 7a). The top enriched motifs in MEF2C binding peaks against GC-matched background were PU.1 and MEF2C motifs (Extended Data Fig. 7b). Motifs for microglia lineage-determining factors, such as AP-1 family members (FOS and Jun), RUNX and CEBP, were also identified, suggesting cooperative regulation with these factors. We then intersected MEF2C-bound peaks with putative enhancer regions that gained or lost H3K27ac in *MEF2C*-KO (Fig. 6a) and MHS (Fig. 6b) microglia. As expected, a larger change was observed when comparing control and KO iMGs rather than control and MHS microglia. From the former intersection, we identified four putative categories of MEF2C regulatory regions: direct activation (presence of MEF2C and loss of H3K27ac in KO iMGs, *n* = 2,313), direct repression (presence of MEF2C and gain of H3K27ac in KO iMGs, *n* = 816), indirect activation (lack of MEF2C and loss of H3K27ac in KO iMGs, *n* = 858) and indirect repression (lack of MEF2C and gain of H3K27ac in KO iMGs, *n* = 1,541; Fig. 6c). With 2,313 direct activation sites and 816 direct repression peaks, MEF2C appears to primarily function as a transcriptional activator in microglia. This pattern also holds true for the subset of MEF2C binding sites found proximal to the TSS (Extended Data Fig. 7c). Mild cross-reactivity of our antibody used for immunoprecipitation with other MEF2 family members, such as MEF2A, may account for the minimal signal in KO samples for MEF2C binding. MEF2C and MEF2A are structurally the most similar members in the MEF2 family^55^, and MEF2A expression in KO iMGs is modestly reduced (Fig. 5c).

To explore the relationship between MEF2C regulation and gene expression, we overlapped nearby genes for each putative regulatory category with DEGs between control and *MEF2C*-KO microglia (Fig. 6c). Although the expression of many nearby genes changed consistently with gain or loss of H3K27ac, most genes related to MEF2C regulatory regions were not statistically different in control and KO microglia. *KCNQ3*, a high-confidence ASD-associated gene, is an example of a gene directly activated by MEF2C whose expression was lost in *MEF2C*-KO microglia (Fig. 6d).

To identify potential transcription factor interactions at MEF2C-related regulatory regions, we performed motif analysis using total peaks as background to enrich for unique motifs. The most significantly enriched motif in direct activation peaks is the MEF2C motif (Fig. 6e and Extended Data Fig. 7d), suggesting that MEF2C binds chromatin directly in its role as a transcriptional activator. At direct repression sites, ETS–RUNX and RUNX motifs were enriched; MEF2C may bind at repressive sites via interactions with RUNX and ETS transcription family members (Fig. 6e and Extended Data Fig. 7d). Enrichment of the ETS–RUNX motif at sites of indirect repression also indicates that these transcription factors operate in a MEF2C-dependent manner without direct interaction with MEF2C itself. Notably, the IRF motif was identified at sites of direct repression, hinting that loss of MEF2C could prime microglia for IFN response via loss of repression on IRF activity (Fig. 6e and Extended Data Fig. 7d).

**Extended Data Fig. 8:**
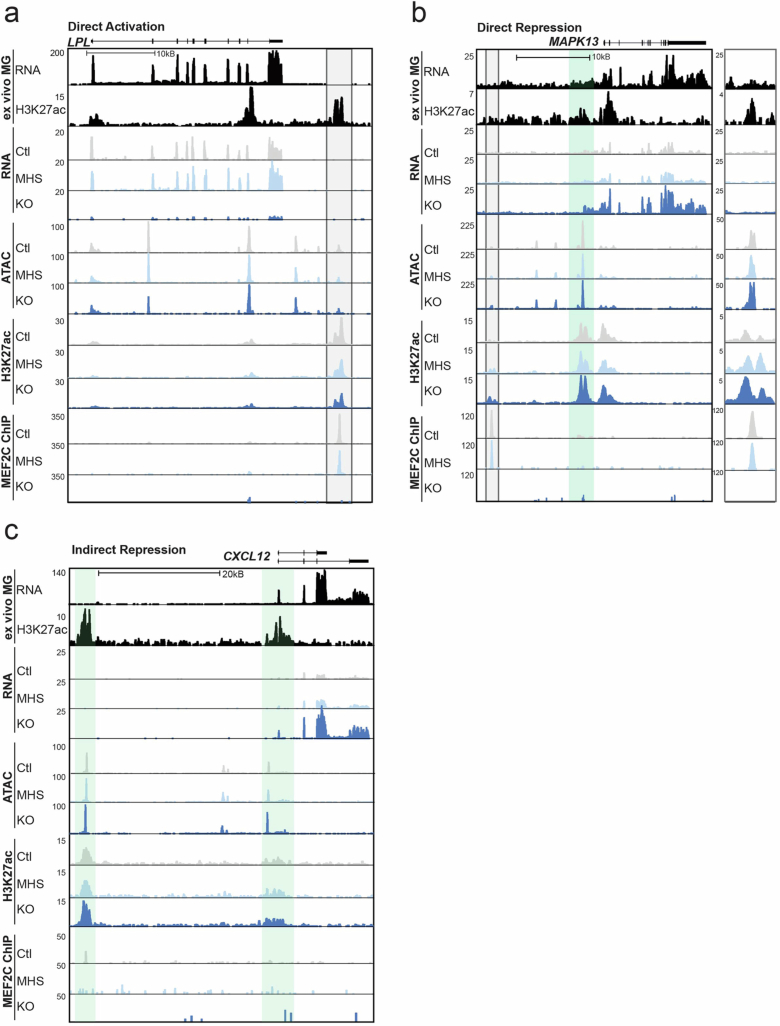
MEF2C regulates key genes related to functional phenotypes. **a**. Genome browser tracks of RNA-seq, ATAC-seq, H3K27ac and MEF2C ChIP-seq from Ctl, MHS and KO iMGs with RNA-seq and H3K27ac ChIP-seq^23,24^ from primary microglia *ex vivo*. *LPL* is a gene regulated by MEF2C-associated direct activation, highlighted by the grey box. **b***. MAPK13* is a gene regulated by MEF2C-associated direct repression, the grey box is highlighted in the right inset and shows direct repression, the light blue box highlights indirect repression at the same gene. **c***. CXCL12* is an exemplar of a gene regulated by MEF2C-associated indirect repression, highlighted by the two grey boxes.

To associate transcriptional regulation with functional consequences, we profiled the set of genes nearest to each MEF2C-associated peak. Gene set enrichment analysis of genes activated by MEF2C identified reactive processes such as cell motility and response to cytokines and endocytosis and maturation processes like neuronal development and the microglial growth factor response (Fig. 6f). Further, the genes activated by MEF2C were enriched in microglia datasets from neurodevelopment, aging and neurodegeneration^56,57^ (Fig. 6f), including lipoprotein lipase (*LPL*) a key regulator of lipid droplet accumulation^58^ (Extended Data Fig. 8a). Meanwhile, genes repressed by MEF2C were related to phagocytosis-associated processes, including actin filament polymerization, Rho GTPase activity and membrane trafficking, as well as lipid metabolism (Fig. 6g). Repressed genes were also primarily enriched in microglia datasets from neurodevelopmental disorders^59,60^ (Fig. 6g). Importantly, identifying MEF2C-regulated genes provides mechanistic insight for the phenotypes observed in *MEF2C*-KO microglia. For example, key MEF2C-repressed genes, including *MAPK13*, which encodes a protein implicated in tau phagocytosis^61^, and *CXCL12*, which mediates microglial migration and process motility^62^, may contribute to the phagocytic deficit of *MEF2C*-KO microglia (Extended Data Fig. 8b,c). Additionally, the lipid profile of KO iMGs can be linked to genes predicted to be regulated by MEF2C-associated repression, such as the key enzymes in phospholipid synthesis *CDS1*, *CHKA* and *PCYT1A*. Overall, the functional phenotype in *MEF2C*-KO microglia may be related to a combination of changes in the activity of other transcription factors and direct MEF2C targets.

**Extended Data Fig. 9:**
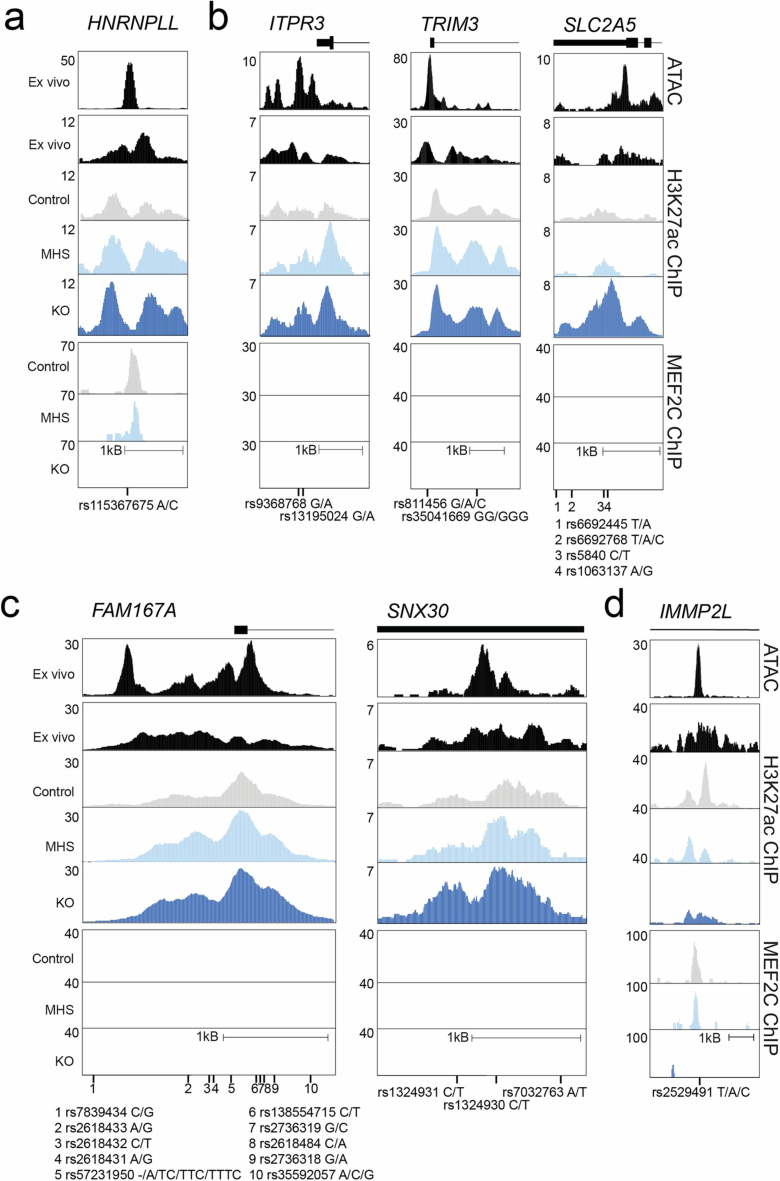
MEF2C-associated epigenetic changes occur at sites with neuropsychiatric disease-related SNPs. Genome browser tracks of H3K27ac and MEF2C ChIP-seq from Ctl, MHS and KO iMGs with ATAC-seq and H3K27ac ChIP-seq^23,24^ from primary microglia *ex vivo* for neuropsychiatric disease-related risk loci regulated by MEF2C-associated direct repression (**a**), indirect repression (**b**-**c**) and direct activation (**d**).

Finally, to connect MEF2C epigenetic regulation to human-specific genetic risk variants involved in neurological phenotypes, we performed linkage disequilibrium score regression analysis on regions of interest. Using genome-wide association study summary statistics, we assessed whether genetic heritability for a phenotype is enriched in single-nucleotide polymorphisms within these regions while accounting for linkage disequilibrium. We found enrichment of genetic variants in autism and neuropsychiatric disorders in regulatory enhancers upregulated with loss of MEF2C, specifically associated with MEF2C activation and not repression (Fig. 6h and Extended Data Fig. 9). Together, MEF2C acts as both a transcription activator and repressor, and the genes regulated in either direction are involved in critical microglial functions and implicated in neuropsychiatric conditions.

### Loss of MEF2C in vivo recapitulates the in vitro phenotype

**Fig. 7:**
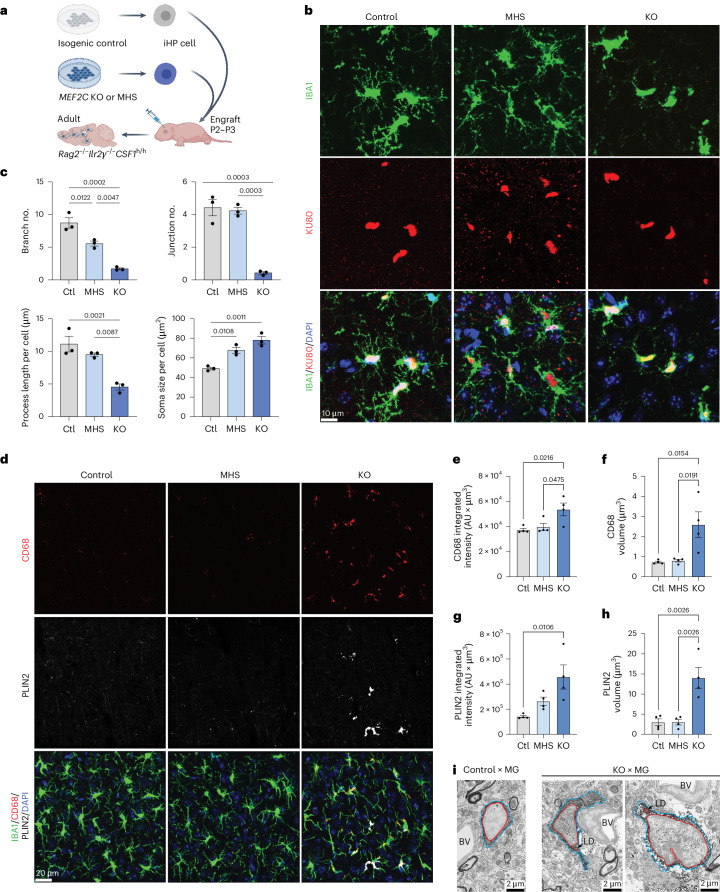
Engraftment of iPS cell-derived MEF2C microglial progenitors into chimeric mouse brain recapitulates a dysfunctional phenotype in vivo*.* **a**, Schematic of the experimental procedure for engraftment of iHP cells from *MEF2C* control, MHS and KO iPS cell lines to humanized, immunosuppressed *CSF1*^h/h^*Rag2*^−/−^*Il2rg*^−/−^ mice. **b**, Representative confocal images of xMGs in chimeric mouse brains demonstrate similar morphological changes in xMGs as observed in the iPS cell-derived in vitro model; KU80, human nuclei marker; IBA1, microglia marker. DAPI, pan-nuclei marker. **c**, Quantification of branch number, junction number, process length and soma size demonstrates a genotype-dependent increase in ameboid morphology in *MEF2C* MHS and KO xMGs (*n* = 3 mice per genotype). **d**, Representative confocal images of cortical xMGs demonstrating increased lysosomal (CD68) mass and lipid droplet (PLIN2) accumulation in KO xMGs. **e**, Quantification of CD68 integrated intensity per IBA^+^ cell (*n* = 3 mice per genotype). **f**, Quantification of CD68 puncta volume (*n* = 3 mice per genotype). **g**, Quantification of PLIN2 integrated intensity per IBA^+^ cell (*n* = 3 mice per genotype). **h**, Quantification of PLIN2 puncta volume (*n* = 3 mice per genotype). **i**, Representative focused ion beam scanning electron microscopy image (resolution of 5 nm) of chimeric mouse brains showing a control xMG with an absence of lipid bodies (left) and two KO xMGs with lipid droplets (LD; right). The human microglia are close to a blood vessel (BV). The red outline indicates the microglial nucleus, the blue outline indicates the microglial cytoplasm, and the orange outline indicates lipid droplets (*n*= 1 mouse per genotype); scale bars, 10 μm (**b**) and 20 μm (**d**). Data in **c** and **e**–**h** are presented as mean values ± s.e.m. Each data point represents the average measurement per mouse. Statistical analyses in **c** and **e**–**h** were performed using one-way ANOVA, with *P* values adjusted for multiple comparisons by Tukey’s method. The graphic in **a** was created with BioRender.com.

**Extended Data Fig. 10:**
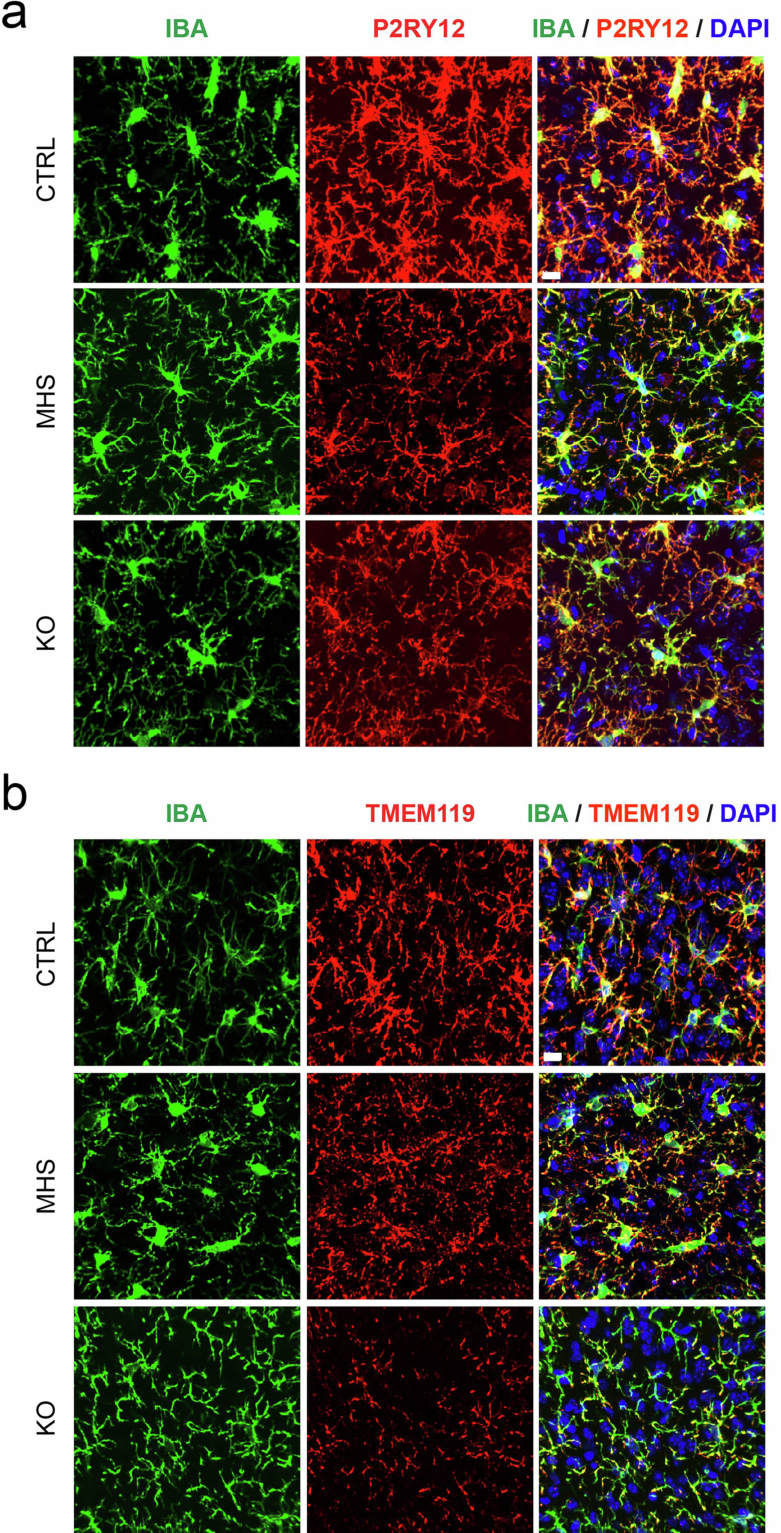
Xeno-transplanted microglia express canonical microglial markers despite loss of MEF2C. **a**. Control, MHS and KO xMGs stain positive for the canonical microglial marker, P2YR12. **b**. Control, MHS and KO xMGs stain positive for the canonical microglial marker, TMEM119. Representative images are shown, results replicated in *n* = 3 mice per genotype. Scale bar = 10 μm in a and b.

Microglia are environmentally regulated and quickly lose their transcriptomic and epigenetic signature after removal from the central nervous system (CNS)^24^. In vitro models recapitulate some, but not all, of these environment-dependent phenotypes^41^. Therefore, to model how loss of MEF2C alters in vivo microglia, we leveraged a chimeric mouse xenotransplantation model^41,63^. Control, MHS and *MEF2C*-KO induced hematopoietic progenitor (iHP) cells were transplanted into an immunodeficient mouse strain that was also humanized for the microglial survival factor CSF-1 (*CSF1*^h/h^*Rag2*^−/−^*Il2rg*^−/−^). At 2 months after transplantation, xenotransplanted microglia (herein, xMGs) from all genotypes expressed some degree of TMEM119, P2YR12 and IBA1 (Extended Data Fig. 10a–c), confirming microglial ontogeny. We then performed morphology analysis as previously described^64^ (Fig. 7a). Compared to both control and MHS xMGs, KO xMGs displayed an overt ameboid morphology, with fewer ramification branch points, shorter average branch lengths and fewer branch junctions per cell (Fig. 7b,c). Notably, MHS xMGs demonstrated an intermediate morphology between ramified and ameboid, indicating that morphological changes occurred in a genotype-dependent manner in vivo. Furthermore, MEF2C-deficient xMGs also recapitulate elevated CD68 lysosomal volume (Fig.7d–f) as well as marked lipid accumulation (Fig. 7d,g,h). PLIN2, a lipid droplet-associated protein, was distinctly present in KO, but not control or MHS, xMGs. As an orthogonal validation of lipid accumulation, we performed focused ion beam scanning electron microscopy and identified round structures with homogenous signal indicative of lipid droplets in KO, but not control, xMGs (Fig. 7i). Together, these quantifications recapitulate the in vitro phenotype and support the findings that gradated loss of MEF2C results in dysfunctional microglia in vivo.

## Discussion

Microglial dysregulation contributes to the pathogenesis of numerous neurological disorders, but phenotypes of microglia in the diseased CNS are heterogeneous and complex. Understanding the network of transcription factors that regulate microglial identity, state and function may provide especially potent therapeutic targets due to the large downstream effects of their transcriptional control. *MEF2C* encodes a transcription factor that regulates microglial identity^24–26^ and is implicated in both diseases of aging and neurodevelopment. The role of MEF2C in neural development has been studied in mouse models^15,16,28,65^, but its role in human brain development and as a regulator of microglial function is not yet well defined. Using an iPS cell-based model, we begin to delineate the role of MEF2C in regulating human microglia function. Human-derived iMGs were generated using a differentiation protocol that recapitulates key aspects of microglia ontogeny and resulted in a differentiated state that is more closely related to in vivo microglia than other tissue-resident macrophage populations, making them a valuable resource for understanding microglial biology. Our investigation indicates that the MEF2C transcriptional program is necessary for maintaining microglial homeostasis, and loss of this epigenetic program leads to dysfunctional and hyperinflammatory microglial phenotypes in the absence of disease or perturbation.

Microglia exist in a variety of cellular states as they respond to their environment during homeostasis and disease, and, over recent years, many microglia gene signatures have been characterized^66^. Although the gene signature of *MEF2C*-KO microglia does not exactly recapitulate a previously described state (that is, damage-associated microglia, proliferative region-associated microglia, lipid droplet-accumulating microglia and so on), there is overlap with multiple datasets, including the transcriptional and epigenetic signatures of ASD^67^, BD and SCZ^30,53^. Furthermore, MEF2C-deficient microglia exhibit many of the functional phenotypes previously described in disease-associated and aging microglia, including unprovoked release of diverse inflammatory mediators, lipid accumulation and impaired phagocytic function. Microglia reactivity has yet to be explicitly identified in individuals with MHS, likely due to the challenges of studying this rare disease, but microglia reactivity has been reported in many neurodevelopmental disorders such as ASD. Inflammation in the developing brain can cause dysregulation of neurogenesis and improper development of neural connectivity^68^, representing potential future pathways for investigation of the microglial contribution to these disease states.

In our in vitro model, changes in both gene expression and functional outcomes were pronounced with full KO of *MEF2C* and more subtle in MHS microglia. Although our *MEF2C* KO appears to capture gene signatures seen in ASD, how the loss of one copy of *MEF2C* in microglia contributes to a similar phenotype is less clear. We have reported that microglial gene expression, including that of *MEF2C*, is environmentally regulated^24^. Therefore, we recognize that our in vitro model may only partially recapitulate the microglial phenotype and thus has limited capacity to capture the complex cellular changes observed in individuals with MHS. Future investigation of human MEF2C-deficient microglia with intact cellular interactions of the CNS in organoid coculture and xenotransplantation models may help further elucidate MEF2C-regulated microglial phenotypes that are relevant to human neurocognitive diseases, including MHS.

Loss of MEF2C in the CNS results in synaptic, cognitive and social deficits in mouse models^15,28,29,65,69^, and the downstream targets of MEF2 in neural development indicate roles for MEF2 in synapse development^70^. This suggests that the neurodevelopmental phenotype seen in individuals with MHS is likely mediated by both microglia and neurons^15,16,28,29,65^. Our transcriptional and epigenetic data identify a network of ASD genes and regulatory elements that are regulated by MEF2C in microglia. Although MHS microglia likely exert influence during fetal brain development, microglia-mediated inflammation may persist and continue to exert effects throughout life. Therefore, microglial MEF2C may serve as a viable therapeutic target to alleviate the effects of persistent neuroinflammation after neurodevelopmental diseases have been diagnosed. Overall, the present study identifies a network of neurodevelopmental and aging-associated genes regulated by MEF2C specifically in microglia. Among the questions remaining to be resolved are the behavioral consequences and the intersection between genetics and environmental stimuli in both development and aging.

## Methods

### Ethics statement

Brain tissue was obtained with informed consent from adult individuals or by informed parental consent and assent when applicable from pediatric individuals under a protocol approved by the University of California, San Diego, and Rady Children’s Hospital Institutional Review Board (UCSD IRB 171361). No compensation for study participation was provided.

### Human tissue procurement for immunostaining

All postnatal individuals were diagnosed with refractory epilepsy and had epileptogenic focus resections at either Rady Children’s Hospital or through the University of California, San Diego, Medical System. Resected brain tissue was immediately placed on ice and transferred to the laboratory for postfixation for histology within 3 h after resection. Charts were reviewed for final pathological diagnosis, epilepsy medications, demographics and timing of stereoelectroencephalography before surgery.

Fetal brain samples were obtained within 1 h of pregnancy termination procedure after informed consent and transported in saline on ice. Tissue was immediately postfixed for histology.

### Immunohistochemistry processing and imaging

Human fetal (*n* = 1, age = gestational week 11, sex unknown) and postnatal tissue (*n* = 2, ages = 11 years and 20 years, male) was fixed in 4% paraformaldehyde (PFA; Electron Microscopy Sciences, 19210) in PBS overnight at 4 °C and transferred to 30% sucrose until the tissue became dense enough to fully sink to the bottom of the tube. The tissue was then embedded in OCT (Fisher Scientific, 23-730-571), and 20-μm sections were collected using a Leica CM1850 cryostat. Sections were immediately mounted on glass slides, and the dried slides were stored at –80 °C until staining. Heat-induced antigen retrieval was performed using 1× Target Retrieval Solution (Dako, S1699) heated to 90 °C for 30 min. After three washes in 0.1 M TBS (Trizma Base, Sigma, T1503, buffered to a pH of 7.5), the slides were treated with blocking buffer (3% normal horse serum and 0.25% Triton X-100 (Sigma, X100) in 0.1 M TBS) for 1 h at 22 °C with gentle rocking. Samples were incubated with primary antibody (see Reporting Summary) in blocking buffer at 4 °C overnight. After three washes in 0.1 M TBS, samples were incubated with secondary antibodies (diluted 1:250 in 0.1 M TBS; see Reporting Summary) for 2 h at 22 °C. Samples were washed with 0.1 M TBS before nuclear counterstaining with 1 μg ml^−1^ DAPI (Thermo Fisher Scientific, 62248). Two more washes with 0.1 M TBS were performed, and the samples were mounted with Shandon Immuno-Mount (Thermo Fisher Scientific, 9990412). Imaging was performed on a Leica TCS SPE confocal microscope, Keyence BZ-X800 Series or Nikon Eclipse Ti2-E with laser-scanning confocal A1R HD.

### Immunohistochemistry quantification

Tiled images of entire slides were acquired on a Keyence BZ-X Analyzer (BZX700) with a ×10/0.45-NA objective. Higher-magnification images were acquired on a Leica TCS SP8 confocal microscope using ×10/0.3-NA, ×40/1.15-NA and ×63/1.4-NA objectives. Images used for quantification were acquired starting at the ventricle wall and moving laterally up to 0.5 mm for MEF2C/IBA1/CTIP2. Ten images per slide were acquired with a thickness of 1 µm for each *z* stack and a total of three slides per sample. Quantifications were performed in Fiji (ImageJ, version2.1.0), and cells were manually counted throughout the *z*-stack images.

### Human pluripotent stem cell culture

All studies were conducted according to the human stem cell (hESCRO) protocol approved by the University of California, San Diego. iPS cell lines, EC11 (derived from primary human umbilical vein endothelial cells from Firth et al.^78^) and GM08330 (ref. ^79^; obtained from K.M. and M.E.T.), were cultured using standard techniques. In brief, cells were cultured in StemMacs iPS-Brew medium (Miltenyi Biotech, 130-104-268) and routinely passaged using Gentle Cell Dissociation Reagent (STEMCELL Technologies, 100-0458) onto plates coated with Cultrex Basement Membrane Matrix (R&D Systems, 3434-010-02). Karyotype was established by standard commercial karyotyping (WiCell Research Institute).

### CRISPR-mediated KO of *MEF2C*

Isogenic *MEF2C* haploinsufficiency and KO were generated using the EC11 iPS cell line with a Synthego Gene Knockout kit and three different guide RNAs to generate three lines each of control, haploinsufficient (MHS) and KO. One control line was derived using a scrambled guide RNA, and the other two control lines were selected from clones that went through the protocol without being edited. Briefly, iPS cells were nucleofected with RNP complex targeting either the MADS or MEF2 domain of MEF2C that mediates DNA binding and were allowed to recover overnight. Three different CRISPR guides were used, of which two specifically target the MADS domain (5’-UCUAGGUGACAUUUACAAAG-3’ and 5’- GAAAUUUGGGUUGAUGAAGA-3’) and a third the MEF2 domain (5’-GAGAAGCACUUUGUCCAUGU-3’). After recovery, cells were dissociated with Tryple (Thermo Fisher, 12604013) and plated at a single-cell density to select for single colonies. Colonies were lifted from the plate with prewarmed Accutase and were mechanically plated to 96-well plates for clonal expansion. Genomic DNA from each colony was amplified and sequenced at the cut site. Loss of *MEF2C* was validated by Sanger sequencing, western blotting and immunostaining. Clonality of heterozygous *MEF2C* KOs was determined through subcloning of at least 20 colonies with Sanger sequencing to validate that the allele segregated appropriately throughout. At least one clone was isolated for each of three CRISPR guides. The frameshift mutations generated and details of each of the MEF2C clones are delineated in Supplementary Table 1. A second cell line (GM038330) that has previously been published^79^ for both control and *MEF2C* KO was also used.

### iHP cell differentiation from iPS cells

Microglia were generated using a two-step protocol as previously described with minor modifications^80^. Briefly, iPS cells were sparsely plated in iPS-Brew with 10 mM ROCK inhibitor (STEMCELL Technologies, 72304) onto Cultrex Basement Membrane Matrix-coated six-well plates^6^ using ReLeSR (STEMCELL Technologies, 100-0484). Cells were differentiated to CD43^+^ hematopoietic progenitors using a StemCell Technologies Hematopoietic kit (STEMCELL Technologies, 05310). On day 1, the medium was removed, and 1× Supplement A was added to the cells. An additional 1 ml per well of 1× Supplement A was added on day 3, and on day 5, the cells were changed to 1× Supplement B. Cells received an additional 1 ml per well of 1× Supplement B on days 7, 9 and 11. The resulting nonadherent iHP cells were collected between days 12 and 14. For in vitro phenotyping, iHP cells were moved to microglia medium and allowed to differentiate as described below. For xenotransplantation studies, iHP cells were frozen and thawed when pups were born.

### Microglial differentiation from iHP cells

iPS cell-derived microglia (referred to as ‘iMGs’) were generated as previously described with minor modifications^81^. Isolated iHP cells were replated onto Cultrex Basement Membrane Matrix-coated plates at a density of 300,000 cells per well in microglia medium (DMEM/F12 (Gibco, 11320033), 2× insulin–transferrin–selenite (Gibco, 41400045), 2× B27 supplement (Gibco, 17504044), 0.5× N2 supplement (Gibco, 17502048), 1× GlutaMAX (Gibco, 35050061), 1× nonessential amino acids (Gibco, 11140050), 800 μM monothioglycerol (Sigma-Aldrich, M6145) and 5 mg ml^−1^ insulin (Sigma-Aldrich, I9278) supplemented with 100 ng ml^−1^ IL-34 (Proteintech, HZ-1316), 50 ng ml^−1^ TGFβ1 (Proteintech, HZ-1011) and 25 ng ml^−1^ M-CSF (Proteintech, HZ-1192)). Cells were supplemented with microglia medium with IL-34, TGFβ1 and M-CSF every other day and differentiated into mature microglia after 28 days, at which time further experiments were conducted. Cell lines were excluded if cells did not grow to sufficient numbers needed for at least two technical replicates for experiments.

### High-throughput morphology analysis

Ramifications were quantified using an IncuCyteS3 Live-Cell Analysis System. Microglia were plated at a density of approximately 300,000 cells per well in a six-well plate coated with Cultrex Basement Membrane Matrix. Phase images were obtained at ×20 magnification in the IncuCyteS3 and then analyzed for ramifications using NeuroTrack software.

### Poly(A) RNA-seq library preparation

For transcriptomic analysis, RNA was extracted from 250,000 mature iMGs from three separate differentiations for each cell line using Trizol-LS reagent. RNA-seq libraries were prepared as previously described. Briefly, mRNAs were incubated with Oligo d(T) Magnetic Beads (New England BioLabs, S1419) and fragmented in 2× Superscript III first-strand buffer (Thermo Fisher Scientific, 18080044) with 10 mM DTT at 94 °C for 9 min. Fragmented mRNA was then incubated with 0.5 μl of random primers (3 mg ml^−1^; Thermo Fisher Scientific, 48190011), 0.5 μl of 50 mM oligo(dT) primer, (Thermo Fisher Scientific, 18418020), 0.5 μl of SUPERase-In (Thermo Fisher Scientific, AM2694) and 1 μl of dNTPs at 50 °C for 1 min. Then, 1 μl of 10 mM DTT, 6 μl of water + 0.02% Tween-20, 0.1 μl of actinomycin D (2 mg ml^−1^; Invitrogen, A7592) and 0.5 μl of Superscript III (Thermo Fisher Scientific, 18080044) were added to the mixture. cDNA was then generated by incubating the mixture in a PCR machine under the following conditions: 25 °C for 10 min, 50 °C for 50 min and a 4 °C hold. Product was purified using RNAClean XP beads (Beckman Coulter, A63987), according to manufacturer’s instructions, and eluted with 10 μl of nuclease-free water. Eluate was then incubated with 1.5 μl of Blue Buffer (Enzymatic, P7050L), 1.1 μl of dUTP mix (10 mM dATP, dCTP and dGTP and 20 mM dUTP), 0.2 μl of RNase H (5 U ml^−1^; Qiagen, Y9220L), 1.2 μl of water + 0.02% Tween-20 and 1 μl of DNA polymerase I (Enzymatics, P7050L) at 16 °C overnight. DNA was then purified using 3 μl of SpeedBeads (Thermo Scientific Fisher, 651520505025) resuspended in 28 μl of 20% PEG8000/2.5 M NaCl to a final concentration of 13% PEG. DNA was eluted with 40 μl of nuclease-free water + 0.02% Tween-20 and underwent end repair by blunting, A-tailing and adaptor ligation, as previously described^10^, using barcoded adapters. Libraries were PCR amplified for 12–15 cycles, size selected by gel extraction for 200–500 bp and sequenced on a HiSeq 4000 (Illumina) or a NextSeq 500 (Illumina) for 51 cycles.

### Bioinformatic analysis

FASTQ files were mapped to the University of California, Santa Cruz, genome build hg38 with STAR (v2.7.9a) using default parameters^23^ and converted to HOMER tag directories. The function ‘analyzeRepeats’ was used to quantify raw reads and then normalize reads as either counts per million (CPM) values or transcripts per million (TPM). A pseudocount of 1 TPM/CPM was added to each gene before base-2 logarithm transformation of TPM/CPM for each gene. Differential expression was performed using R (v4.0.4) and the package DESeq2 (v1.30.1) with an FDR of <0.05 and log_2 _(FC) of >1. In addition to pairwise comparisons, a linear model was set up to compare control microglia, MHS microglia and KO microglia using the R packages DEGreport (v1.26.0) and DEseq2.

For gene set comparisons from RNA-seq library preparation, we generated lists for the published datasets by using an FC cutoff (modified after refs. ^48,82^) and compared these lists with the top upregulated and downregulated genes in *MEF2C* control and KO microglia. For PsychEncode enrichment analysis, we pulled the WGCNA module information from PsychEncode^30^ and performed Fisher’s exact tests to determine gene set overlap odds ratios and significance with an adjusted –log_10_ (*P* value) of <0.05.

### Unbiased complex lipidomic analysis by mass spectrometry

Samples were extracted using the Matyash extraction procedure, which includes methyl *tert*-butyl ether, methanol and water. The organic (top) phase was dried down and submitted for resuspension and injection onto a reverse-phase liquid chromatography–high-resolution tandem mass spectrometer for lipids analysis. All processing of the liquid chromatography–mass spectrometry raw data files was performed using MS-DIAL v5.5 software for data collection, peak detection, alignment and adduct ion and lipid identification^83^. The detailed parameter settings were as follows: MS^1^ tolerance, 0.01 Da; MS^2^ tolerance, 0.025 Da; minimum peak height, 500,000 amplitude; mass slice width, 0.05 Da; smoothing method, linear weighted moving average; smoothing level, three scans; minimum peak width, five scans. [M + H]^+^, [M + NH_4_]^+^, [M + Na]^+^, [2M + H]^+^ and [M + H – H_2_O]^+^ and [M – H]^−^, [M – 2H]^2–^, [M + HCOO]^−^ and [M – H – H_2_O]^−^ were included in adduct ion setting for positive and negative mode, respectively. Compounds were annotated by *m*/*z* and tandem mass spectrometry spectra against the LipidBlast mass spectra database^84^. Internal standards were monitored for retention time and intensity, and principal component analysis was used for multivariate statistics and visualization, specifically for outlier detection. From the MS-DIAL results file, all detected features/metabolites were removed if (sample max) / (blank average) < 10. Known (positively identified/annotated) feature/metabolite sample peak heights were normalized to the cell number. Known (positively identified/annotated) features/metabolites were manually evaluated when flagged by specific parameters that required further investigation of identifications. Remaining replicate features were filtered based on manual evaluation of spectra, MS-DIAL total score, dot product and quality control pooled sample relative standard deviation. Following removal based on the previously mentioned sample max/blank average, all features not positively identified (unknown compounds) that generated both *m*/*z* and MS^2^ data were retained and are reported separately. Unknown compound sample peak heights were normalized to the cell number.

### ATAC–seq and ChIP–seq analysis

HOMER was used to call peaks from the ATAC–seq mapped tag directories, and only ATAC peaks with an irreproducible discovery rate of <0.05 was used for downstream analysis. Peak lists from *MEF2C* control, MHS and KO iMG ATAC were merged with HOMER’s mergePeaks and annotated for H3K27ac reads with HOMER’s annotatePeaks. DESeq2 was used to identify ATAC peaks that are differentially acetylated between *MEF2C* control and KO iMG groups. The peaks with increased acetylation in *MEF2C* control versus KO were overlapped with *MEF2C* peaks to identify direct and indirect *MEF2C* activation regions. The peaks with increased acetylation in *MEF2C* KO versus control were overlapped with *MEF2C* peaks to identify direct and indirect *MEF2C* repression regions. HOMER’s findMotifsGenome.pl was used to identify default and de novo motifs from the final peak lists for *MEF2C* direct and indirect activation and repression sites. The background peaks used were the random genome sequences generated by HOMER. ASD-associated distal H3K27ac-marked peaks were pulled from Ramaswami et al.^53^ and cell-type-specific promoter and distal enhancer genomic locations from Nott et al.^23^. Overlapping genomic ranges were determined using the package GenomicRanges (v.1.54.1) and plotted as the total number of cell-type-specific overlapping peaks for promoters and distal enhancers. Ensembl Assembly Converter (v.111) was used to convert peaks from hg19 to hg38 genomic ranges and pypher (v.0.7.1) for hypergeometric *P* value significance of overlap.

### Mice

*Rag2*^−/−^*IL2rg*^−/−^*CSF1*^h/h^ (referred to as ‘RIM’) mice were purchased from The Jackson Laboratory (strain 017708) and were bred and maintained in local barrier facilities. Mice were housed in groups of two to five individuals under a 12-h light/12-h dark cycle at 22 °C, with food and water available ad libitum. All described animal procedures were approved by the University of California, San Diego, Institutional Animal Care and Use Committee at an Association for Assessment and Accreditation of Laboratory Animal Care International-accredited facility (protocol S19162).

### Endogenous mouse microglial depletion

The CSF1R inhibitor BLZ945 was dissolved in 20% (2-hydroxypropyl)-β-cyclodextrin (Sigma-Aldrich, 332607). Newborns were injected (intraperitoneally) 24 h before human cell transplantation at a dose of 200 mg per kg (body weight) as previously described^63^.

### Early postnatal intracerebroventricular xenotransplantation and brain tissue processing

Xenotransplantation was performed as recently described^50,64^. Briefly, iHP cells were thawed and plated into complete microglia medium and allowed to recover for 2 days before transplantation. Twenty-four hours before engraftment, the endogenous mouse microglia niche was depleted. The next day, postnatal day 2–3 RIM mice (*n* = 3 mice per genotype) were placed in a clean cage over a heating pad with a nestlet from the home cage to maintain the mother’s scent. Pups were then placed on ice for 2–3 min to induce hypothermic anesthesia. Free-hand transplantation was performed using a 30-gauge needle affixed to a 10-μl Hamilton syringe, mice received 1 μl of iHP cells suspended in sterile 1× DPBS at 30,000–50,000 iHP cells per μl at each injection site (see ref. ^41^). Equal numbers of pups in each litter received control, MHS and *MEF2C-*KO lines. Bilateral injections were performed at two-fifths of the distance from the lambda suture to each eye, injecting into the lateral ventricles at 3 mm and into the overlying anterior cortex at 1 mm and into the posterior cortex in line with the forebrain injection sites and perpendicular to lambda at a 45° angle. Transplanted pups were then returned to their home cages and weaned at postnatal day 21. At 2 months of age, engrafted mice were transcardially perfused with ice-cold PBS followed by 4% PFA. Tissue was postfixed in 4% PFA overnight before transfer to 30% sucrose. Tissue processing and staining was performed as described above under ‘Immunostaining processing and imaging’. Only male mice were used in this study. Other than survival to 2 months of age, no exclusion criteria were used.

### Morphological analysis of xenotransplanted microglia

Xenotransplanted microglia were morphologically assessed as previously described^64,85^. Briefly, human microglia were identified as KU80^+^ (human nuclei marker) and IBA1^+^ (microglia/macrophage marker). Skeleton analysis was then performed using the ImageJ plugin AnalyzeSkeleton^86^. Fractal analysis was then performed using the ImageJ plugin FracLac^87^. The outputs of these plugins summarize cell morphology in terms of process endpoints, junctions and length as well as complexity, cell shape and soma size.

### Statistics

All statistical analyses were performed using GraphPad Prism v10. No statistical tests were used to predetermine sample sizes, but our sample sizes are similar to those used in previous publications^8,23,50,64^. Except for experiments using the IncuCyteS3 Live-Cell Analysis System, data collection and analysis were not performed blind to the conditions of the in vitro experiments. For in vivo xenotransplantation quantitation, the individual performing the analysis was blinded to the genotype of the xenotransplanted microglia. Cell seeding in plates and slides and pup engraftments were performed in a random order to implement randomization in experiments. Data distribution was assumed to be normal, but this was not formally tested. Unless otherwise noted in the figure legends, mean measurements between three groups were compared by one-way ANOVA with Tukey’s multiple comparison tests. All *P* values were two sided. Significance was determined by a *P* value of <0.05. Error bars in plots represent s.e.m.

### Reporting summary

Further information on research design is available in the Nature Portfolio Reporting Summary linked to this article.

## Online content

Any methods, additional references, Nature Portfolio reporting summaries, source data, extended data, supplementary information, acknowledgements, peer review information; details of author contributions and competing interests; and statements of data and code availability are available at 10.1038/s41590-025-02299-0.

## Supplementary information

Supplementary Information Supplementary Methods.

Reporting Summary

Supplementary Table 1 CRISPR guides used for MEF2C-deficient iPS cell lines.

## Source data

Source Data Fig. 1 Unprocessed western blots for Fig. 1f.

Source Data Fig. 2 DEG list, enrichment for pathways, upstream regulators and microglia gene lists.

Source Data Fig. 3 Unprocessed western blots for Fig. 3j.

Source Data Fig. 4 Lipidomics ANOVA results and normalized measurements.

Source Data Extended Data Fig. 3 IPA enrichment for pathways and upstream regulators for genes down in *MEF2C-*KO compared to control iMGs.

Source Data Extended Data Fig. 4 Genes associated with modules defined by WGCNA.

## Data Availability

Previously reported data are available from Gene Expression Omnibus, including those from Gosselin et al.^24^ (GSE62826) and Han et al.^5^ (GSE226690). PLAC–seq data are available on dbGAP from Nott et al.^23^ (phs001373.v2.p2). Data generated from this study are accessible under the SuperSeries GSE306993. Source data are provided with this paper.
