# Supplementary material for: Transcriptional and epigenetic targets of MEF2C in human microglia contribute to cellular functions related to autism risk and age-related disease
Source: Nat Immunol. 2025 Oct 22;26(11):1989–2003. doi: 10.1038/s41590-025-02299-0 (PMC12571900; doi:10.1038/s41590-025-02299-0)
Supplement: Supplementary file 1 — Supplementary Methods. [file 41590_2025_2299_MOESM1_ESM.pdf]

nature immunology

Supplementary information https://doi.org/10.1038/s41590-025-02299-0

Transcriptional and epigenetic targets of MEF2C in human microglia contribute to cellular functions related to autism risk and age-related disease

In the format provided by the authors and unedited

Supplementary Methods

Western Blot

MEF2C knockout in iPSC-derived microglia was confirmed by Western blotting. One million iMGs were lysed in 1X RIPA buffer (Millipore #20-188) supplemented with 1X protease/phosphatase inhibitors (Thermo Fisher Scientific #78443). Lysate was run on 4-12% NuPAGE acrylamide gels (Invitrogen #NP0321BOX) in 1X MOPS buffer (Invitrogen #NP0001) for 30 min at 150V followed by 45 min at 180V and transferred to 0.45 µm nitrocellulose blotting membrane (Amersham #1060002) at 30V for 1.5 h. Blocking was performed with 4% milk (Cell Signaling Technologies #9999S) for 1 h. MEF2C protein was detected with 1:1000 rabbit anti-MEF2C primary antibody (Abcam #AB211493) incubated overnight at 4°C with gentle rocking, followed by 1:2000 goat anti-rabbit IgG HRP-linked secondary antibody (Cell Signaling Technology #7074S) for 2 h at 22°C. GAPDH was used as a loading control and detected with 1:5000 mouse anti-GAPDH (Santa Cruz, #sc-47724) and 1:2000 goat anti-mouse IgG HRP-linked secondary antibody (Cell Signaling Technology #7076S). Pierce ECL Western Blotting Substrate (Thermo Fisher Scientific #32106) was used to visualize protein bands in a ChemiDoc MP Imaging System (BioRad). MEF2C expression relative to GAPDH expression was quantified in ImageJ.

Fluorescence-Activated Cell Sorting for microglia markers

Mature iMGs were washed once in PBS (Gibco #14190-144), spun down, and then resuspended in FACS sorting buffer: HBSS (Gibco #14175-095), 1% BSA (Sigma-Aldrich #A9647-10G), 1 mM EDTA (Sigma-Aldrich #E-7889). The cell suspension was blocked in 1:20 human TruStain FCX (BioLegend #422302) for 5 min at 22°C. Cell suspension was stained for 20 min on ice using 1:200 fluorophore-conjugated antibodies: CD64 (BioLegend #305014), CX3CR1 (BioLegend #341614), CD68 (BioLegend #333812), CD11b (BioLegend #301306), HLADR (BioLegend #307616), and CD45 (BioLegend #304014). From each sample, 10 µL of the blocked cell suspension were collected prior to antibody incubation and pooled for an isotype control. Isotype control antibodies for APC (BioLegend #400122), PerCP-Cy5.5 (BioLegend #400632), 488 FITC (BioLegend #400129), PE (BioLegend #400112), PE-Cy7 (BioLegend #400232), and APC Cy7 (BioLegend #400128) were added to the isotype control sample. Zombie violet (BioLegend #423114) was added at a concentration of 1 µg/mL to determine cell viability. The sample was then washed and resuspended in FACS buffer. A BD FACSAria™ Fusion cytometer was used to sort for live cells and measurement of fluorescence intensity for all markers.

Quantification of microglia markers of iMGs by immunostaining

iMGs were seeded in chambered glass slides coated with Cultrex Basement Membrane Matrix for at least 24 h to allow the cells time to adhere. The cells were washed briefly with PBS to remove excess media and fixed in 4% PFA for 10 min at 22°C. After three washes in PBS, the cells were treated with blocking buffer (3% normal horse serum and 0.25% Triton X-100 in 0.1M TBS) for 1 h at 22°C, then stained and imaged as described under "*Immunohistochemistry processing and imaging*". Images from stained iMGs were processed using ImageJ and image quantification was performed on 4 wells per line and with 4 images per well. Percent of positive cells was quantified by manually counting the number of positive cells per image and normalizing to the number nuclei. Nuclei were quantified by analyzing the number of DAPI+ nuclei above a set threshold. Intensity was measured using ImageJ as a mean gray value and then normalized to the number of DAPI+ nuclei as previously described[7].

WGCNA

Weighted gene co-expression network analysis (WGCNA), using filtered $\log_2$ transformed expression data for Control MEF2C microglia and KO microglia. This tool was then applied to

construct scale-free networks that specify coordinately regulated genes (i.e., modules) [10]. To explore the modular structures of the co-expression network, the adjacency matrix was transformed into a topological overlap matrix [10]. Because topological overlap between two genes reflects both their direct and indirect interactions with all other genes in the network, this approach helps create more cohesive and biologically meaningful data interpretation. WGCNA parameters optimized including a minimum module size equal to 75 genes and a 0.99 tree-cut height. The maximal statistical significance for gene set enrichment was determined using a Fishers-exact test, corrected for multiple comparisons using a Benjamini-Hochberg false discovery rate (FDR) < 0.05. Each module was assigned a unique, arbitrary color and number. Individual gene sets from modules and differentially expressed genes were functionally annotated for biological processes and pathway enrichment using the web-based software application Metascape [11] and selected if they passed an adjusted P-value < 0.05. Select gene sets, based upon statistical and biological significance, were assembled to visualize network properties using the bioinformatics tool Cytoscape (v3.8.1)[12].

Phagocytosis assay

Phagocytosis was measured using an IncuCyteS3 Live-Cell Analysis System. Microglia were plated with three lines each of control, MHS, and KO in 3 wells per line in a 24-well Primaria plate (Corning #353847) at a density of 50,000 cells per well. Just prior to imaging in the IncuCyteS3, 100 μL of either pHrodo Red Zymosan BioParticles (Thermo Fisher Scientific #P35364) at a concentration of 20 μg/mL, pHrodo Red *S. aureus* BioParticles (Thermo Fisher Scientific #A10010) at 75 μg/mL or amyloid beta conjugated to 488 at 1:1000 were added per well. Images were obtained every 5 min after the addition of substrate for a total of 2-5 h. Phagocytosis was measured in the IncuCyteS3 by determining the total number of cells containing fluorescent bioparticles normalized to the number of cells.

Lysotracker assay

Microglia were seeded at a density of 50,000 cells per well in a 4-well slide (Ibidi #80426). Cells were allowed to adhere for 24 h before proceeding. Cells were then incubated with Lysotracker (50 nM) (Thermo Fisher Scientific #L7528) for 20 min at 37°C. Then cells were washed thrice with DPBS followed by incubation in 5 μg/mL Hoechst 33342 stain (ThermoFisher #H3570) for 10 min at 22°C. Cells were then washed 3 more times with DBPS for before imaging on a Leica TCS SPE confocal microscope under the 10x objective.

iNOS assay

Microglia were seeded at a density of 25,000 cells per well in a 96-well Primaria plate (Corning, #353847) then incubated overnight at 37°C. iNOS was then detected using the Intracellular Nitric Oxide Synthase Detection Assay Kit (Abcam #ab211085). Briefly, cells were washed twice with 200 μL Assay Buffer each. A 1X Working Solution of the Staining Dye was made immediately prior to use (1:200 dilution). 20 μL/well of diluted Staining Dye were added to each well containing cells. Cells were incubated for 1 h at 37°C in the incubator, protected from light. Cells were then washed with assay buffer to remove excess dye and then imaged using the Incucyte S3 live imaging system.

CellROX assay

Microglia were seeded at a density of 50,000 cells per well in a 4-well slide (Ibidi #80426). Cells were allowed to adhere overnight before proceeding. Cells were then incubated with CellROX (5 μM) (Life Technologies #C10422) for 30 min at 37°C. Then cells were washed thrice with PBS followed by incubation in 5 μg/mL Hoechst 33342 stain (Thermo Fisher Scientific #H3570) for 10

min at 22°C. Cells were then washed more three times with before imaging on a Leica TCS SPE confocal microscope under the 10x objective.

Cytokine multiplexed ELISA
Cytokine profiling was carried out using MesoScale Discovery V-PLEX Proinflammatory Cytokine Panel II (Human) ELISA kits (IL-1β, TNF, IL-6 and IL-8), following the manufacturer's instructions (MSD #K15053D). Supernatants from microglia cultures (Control, MHS, and KO) were plated. Three parallel wells as technical replicates were measured per single data point for each biological replicate.

Acute interferon (IFN)-β stimulation
iMGs were seeded in 12-well plates coated with Cultrex Basement Membrane Matrix at a density of 500,000 cells per well for at least 24 h to allow the cells time to adhere. Either microglia media with 20 ng/mL human recombinant IFN-β (R&D Systems #8499-IF-010/CF) or and media with PBS was added to the iMGs for 30 min before cells were collected for protein analysis. Cell pellets were lysed, and protein was analyzed as described above under "Western Blot" for pSTAT1, STAT1 and GAPDH. Stimulation was performed on three independent microglia differentiations.

Visualizing BODIPY via microscopy
Microglia were seeded at a density of 80,000 cells per well in a 96-well Primaria plate (Corning #353847). Cells were washed once with PBS and then incubated in 1 μg/mL BODIPY 493/503 (Thermo Fisher Scientific #D3922) for 15 min at 37°C. Following staining, microglia were washed twice with DPBS and were then fixed in 4% PFA (Electron Microscopy Sciences #19210) for 30 min. Then cells were washed once with DPBS followed by incubation in 5 μg/mL Hoechst 33342 stain (Thermo Fisher Scientific #H3570) for 10 min at 22°C. Finally, cells were washed three times with DBPS for 5 min per wash and then imaged on a Leica TCS SPE confocal microscope under the 10x objective.

Flow cytometry of BODIPY+ microglia
Microglia were collected and re-suspended in 1 mL PBS. 100 μL of cell suspension was transferred to a separate conical to serve as an unstained control. The cell suspension was then centrifuged at 300 x g for 5 min. Cell pellets were resuspended in 2 mL of 0.5 μg/mL BODIPY 493/503 (Thermo Fisher Scientific #D3922) in PBS and incubated for 20 min at 37°C to stain lipid droplets. After incubation, 10 mL of PBS was added to each conical and followed by centrifugation for 5 min at 300 x g to wash the cells. A second wash was performed by resuspending the cell pellet in 5 mL PBS and again centrifuging for 5 minutes at 300 x g. Microglia were finally resuspended in 1 μg/mL Zombie Violet (BioLegend #423114) in HBSS sorting buffer: HBSS (Gibco #4175-095), 1% BSA (Sigma Aldrich A9647-10G), 1mM EDTA (Sigma Aldrich #E-7889) to assess viability. The cell suspension was then transferred to flow cytometry tubes (Falcon #352052) and sorted for live, BODIPY positive cells using a BD LSRFortessa X-20.

Chemical enrichment analysis on lipidomic data
Chemical similarity enrichment analysis, a form of enrichment based on chemical ontologies and structural similarity, was executed using ChemRICH software (Barupal et al. 2017).

Amyloid beta preparation and aggregation
Amyloid beta (1 mg; Anaspec #AS-20276) was diluted in 1.0% NH_4OH as the solvent and brought to a final concentration of 1 mg/mL with PBS. Amyloid beta 1-42 fibrils were formed by shaking at 300 x g at 37°C for 5 days as previously described[17].

Scratch migration assay
Motility was observed using Essen Incucyte WoundMaker Assay. 50,000-100,000 iPSC-microglia were plated per well on fibronectin-coated 96-well plate (n = 4 wells) for 1 h for cells to become adherent. Scratches were repeated 4 times. Cells were imaged continuously until 48 hours post-scratch. Wound confluence (confluence of cells within original wound region of interest; ROI) was calculated using Incucyte 2019B Software as previously described[18].

Transwell migration assay
Cell migration was evaluated using a Transwell assay (8 μm pore size, a polycarbonate membrane, Corning #3422). Briefly, iMGs were seeded in the upper compartment of a Transwell at 50,000 cells/cm^2 and maintained in a monoculture. In the bottom chamber, 600 μl of chemo-attractant media (amyloid beta 1-42 [1mM], tau-441 [1μM]) or the same volume of media was added. After 24 h, cells on the top compartment were removed and the transwells were fixed in 4% PFA for 30 min. Transwells were allowed to dry for 10 min and stained with 1 μg/mL DAPI solution (Thermo Fisher Scientific #62248) for 10 min at 22°C. The migrating microglia accumulating on the other side of the Transwell membrane were quantified using ImageJ. Threshold was attuned and particle analysis was performed to obtain the total number of cells.

Epigenetic sequencing library preparation
ATAC: ATAC-seq library preparation was performed as previously described on 50,000 cells per cell line per replicate. Live cells were rinsed with 1X PBS and lysed with cold 50 μL lysis buffer (10 mM Tris-HCl pH 7.5, 10 mM NaCl, 3 mM $MgCl_2$, 0.1% IGEPAL CA-630). After centrifugation at 500 x g for 10 min at 4°C, the resulting nuclei pellet was re-suspended in 50 μL tranposase reaction mix (Illumnia #20034197) and incubated at 37°C for 30 min. The Zymo ChIP DNA Clean and Concentrator-5 kit (Zymo Research D5205) was used to purify the DNA, which was then amplified with Nextera primers (1.25 μM) using NebNext High-Fidelity 2X PCR Master Mix (New England Biolabs MO541). The final libraries were purified by gel excision of 155-250 bp fragments and single-end sequenced.

ChIP: Chromatin ImmunoPreciptation-seq library preparation was performed as previously described on 1,000,000 cells per cell line per replicate. For H3K27ac ChIP, the cells were fixed in 1% PFA, pelleted at 1000 x g for 5 min at 4°C, and flash frozen. Frozen cell pellets were lysed on ice in ice-cold LB3 (10 mM Tris-HCl pH 7.5, 100 mM NaCl, 1 mM EDTA, 0.5 mM EGTA, 0.1% Na-deoxycholate, 0.5% *N*-lauroylarcisine, 1X protease inhibitor cocktail). Chromatin was sheared with sonication using a Covaris E220. Samples were spun at maximum speed at 4°C for 10 min to remove debris and one percent of the supernatant, which contained the DNA for immunoprecipation, was saved as input. The H3K27ac antibody (Active Motif 39685, 1 μg per sample) was coupled to Dynabeads Protein A (Invitrogen 10002D, 25 μL per sample) and incubated with sheared chromatin overnight on a rotator at 4°C. The beads were washed: 3 times with Wash Buffer I (20 mM Tris-HCl pH 7.5, 150 mM NaCl, 2 mM EDTA, 0.1% SDS and 1% Triton X-100, 1X protease inhibitor cocktail), 3 times with Wash Buffer III (10 mM Tris-HCl pH 7.5, 250 mM LiCl, 1% Triton X-100, 1mM EDTA, 0.7% Na-deoxycholate, 1X protease inhibitor cocktail), twice with TET buffer (0.2% Tween-20/TE, $\frac{1}{3}$X protease inhibitor cocktail), once with TE-NaCl (50 mM NaCl/TE) and once with IDTET (0.2% Tween-20, 10 mM Tris pH 8, 0.1 mM EDTA) buffer. Final samples were resuspended in 25 μL TT buffer (10 mM Tris pH 8, 0.05% Tween-20) for on-bead library preparation. For MEF2C ChIP, cells were fixed in 2 mM disuccinimidyl glutarate (CovaChem 13301) for 30 min and then in 1% PFA at 22°C. Frozen fixed cell pellets were lysed on ice in ice-cold RLNR1 buffer (20 mM Tris-HCl pH 7.5, 150 mM NaCl, 1 mM EDTA, 0.5 mM EGTA, 0.4% Na-deoxycholate, 1% NP-40, 0.1% SDS, 0.5 mM DTT, 1X protease inhibitor cocktail/PMSF) and sonicated as for H3K27ac ChIP. The MEF2C antibody (Cell Signaling

Technology #5030, 1 μg per sample) was coupled with a 1:1 mixture of Dynabeads Protein A and G (Invitrogen 10002D and 10004D, 10 μL of each per sample) and incubated with sheared chromatin overnight on the rotator. The beads were washed: 3 times with RLNR1 buffer, 6 times with LWB-RCNR1 buffer (10 mM Tris-HCl pH 7.5, 1 mM EDTA, 0.7% Na-deoxycholate, 1% NP-40, 250 mM LiCl, 1X protease inhibitor cocktail/PMSF), 3 times with TET buffer, twice with IDTET buffer. Washed beads were resuspended in TT buffer and all inputs were diluted in TT buffer to match the volumes of the ChIP samples. Library preparation was perform using the NebNext Ultra II DNA Library Prep Kit (New England Biolabs E7645). Crosslinking was reversed with overnight proteinase K treatment. The DNA was purified using SpeedBeads (Thermo Scientific Fisher 651520505025) in 20% PEG8000, 1.5 M NaCl to final 12% PEG concentration and then amplified using NebNext High-Fidelity 2X PCR Master Mix (New England Biolabs MO541). The final library was purified by gel excision of 200-500 bp fragments and single-end sequenced.

Linkage Disequilibrium Score Regression (LDSC)
LDSC was used to evaluate heritability enrichment for a panel of aging and neuropsychiatric traits across genomic regions of interest. By analyzing the relationship between GWAS effect sizes and linkage disequilibrium (LD) between SNPs, LDSC quantifies the contributions of both polygenicity and confounding biases, allowing for the estimation of trait heritability and its distribution across specific genomic regions[16]. European ancestry LD scores and weights were obtained from the 1000 Genomes Phase 3 SNPs as a reference panel, while summary statistics were derived from GWAS studies conducted on autism, Alzheimer's, and neuropsychiatric disorders[17-22]. Annotated SNP lists were created for all SNPs within ±5 kb of ATAC-seq or ChIP-seq peaks of interest, corresponding to the regions where heritability enrichment was being assessed. The Bulik-Sullivan LDSC package was adapted to carry out the calculations, following the recommended LDSC baseline model[23].

Electron microscopy tissue preparation and immunostaining
Mice designated for electron microscopy were transcardially perfused with ice-cold PBS followed by 4% PFA/0.4% glutaraldehyde (Electron Microscopy Sciences #111-30-8). Brains were post-fixed in 4% PFA/0.4% glutaraldehyde for 2 h at 4°C before being washed with PBS for 10 min three times. The fixed tissue was stored in PBS at 4°C until being embedded in 4% agarose. Once the agarose solidified, the tissue was sectioned into 50 μm coronal sections using a vibratome (VT1200S, Leicha Biosystems). Brain sections were stored in cryoprotectant (40% PBS, 30% ethylene glycol, 30% glycerol) at -20°C until further processing. For imaging, brain sections containing the ventral hippocampus cornu ammonis 1 (CA1) from 2-month-old mice (Bregma -2.92 mm to -3.16 mm) were selected. The chosen sections were quenched with 0.3% H_2O_2 (Fisher Scientific #202762) in PBS (50 mM, pH 7.4) for 5 min. Afterwards, the sections were incubated in 0.1% $NaBH_4$ in PBS for 30 min followed by 3 washes of 10 min in PBS. Brain sections were then incubated in a blocking buffer solution containing 10% fetal bovine serum (Jackson ImmunoResearch Labs #005-000-121), 3% bovine serum albumin (Sigma-Aldrich #9048-46-8,), and 0.01% Triton X-100 in PBS for 1 h at 22°C. They were then incubated overnight in blocking buffer solution with a primary rabbit anti-Iba1 antibody (1:1000; FUJIFILM Wako Chemical #019–19741) at 4°C. The following day, the brain sections were washed with TBS (50 mM, pH 7.4) then incubated with a biotinylated goat anti-rabbit polyclonal secondary antibody (1:300; Jackson ImmunoResearch #111-066-046) in TBS for 2 h at 22°C. Afterwards, the sections were washed in TBS and incubated for 1 h at 22°C in an avidin-biotin complex solution (ABC; 1:100; Vector Laboratories PK-6100) in TBS. The staining was revealed with 0.05% 3,3'-diaminobenzidine (DAB; Millipore Sigma #D5905-50TAB) and 0.015% H_2O_2 diluted in Tris buffer (TB; 0.05 M, pH 8.0).

Tissue processing for scanning electron microscopy

The immunostained brain sections were incubated in 3% potassium ferrocyanide (in PB; BioShop #PFC232.250) combined (1:1) with 4% aqueous osmium tetroxide (Electron Microscopy Sciences #9170,) for 1 h, washed in phosphate buffer (PB; 100 mM, pH 7.4), incubated in 1% thiocarbohydrazide (in double distilled water (ddH_2O); Electron Microscopy Sciences #2231-57-4,) for 20 min, washed in ddH_2O, incubated in 2% osmium tetroxide (in ddH_2O), then dehydrated in ascending concentration of ethanol (2 times in 35%, 50%, 70%, 80%, 90%, and 3 times in 100%) followed by 3 incubations in propylene oxide. Post-fixed sections were embedded in Durcupan ACM resin (MilliporeSigma #44611–44614) for 24 h, placed between two ACLAR® sheets (Electron Microscopy Sciences 50425-25) for flat-embedding and resin was polymerised at 55 °C for 72 h. Regions of interest were excised, re-embedded on a resin block, and cut into 73-nm ultrathin sections using a Leica ARTOS 3D ultramicrotome (Leica Biosystems). The ultrathin sections were mounted on dust-free silicon nitride chip, glued on specimen mounts, and inserted into a Zeiss Crossbeam 350 focused ion-beam scanning electron microscope (FIB-SEM). The samples were imaged using backscattered electrons (ESB) and secondary electrons (SE2) detectors at 10 kV for initial focus, then at 1.4 kV when samples were at a sufficient working distance to perform high-resolution focus on the hippocampal ultrastructure. These steps were controlled using SmartSEM software (Fibics). Microglial cell bodies, identified by their overall size and shape, unique heterochromatin pattern, associated extracellular space pockets, as well as long and narrow stretches of endoplasmic reticulum among other key defining ultrastructural features [34,35] were imaged at a 5 nm resolution and exported as TIFF file format using the Zeiss ATLAS Engine 5 software (Fibics).

Antibodies

Antibody	Manufacturer	Catalog #	Dilution	Application
Rabbit anti-MEF2C	Abcam	ab211493	1:200, 1:1000	IF, WB
Goat anti-IBA1	Abcam	ab5076	1:200	IF
Rabbit anti-IBA1	Wako	019-19741	1:500/1:1000	IF/EM
Rat anti- CTIP	Abcam	ab18465	1:200	IF
Mouse anti-CD68	Dako	M0814	1:200	IF
Mouse anti-LAMP1	Invitrogen	14-1079-80	1:200	IF
Rat anti-LAMP2	Abcam	ab13524	1:250	IF
Goat anti-B-gal	Biogenesis	103006	1:250	IF
Goat anti-TREM2	R&D system	AF1828	1:100	IF
Rabbit anti-APOE	Invitrogen	701241	1:100	IF
Rabbit anti-Ku80	Abcam	ab80592	1:100	IF
Rabbit anti-pSTAT1	Cell Signaling Technology	#8826	1:1000	WB
Rabbit anti-STAT1	Cell Signaling Technology	#14994	1:1000	WB
Mouse anti-GAPDH	Santa Cruz Biotech.	Sc-47724	1:5000	WB
Rabbit anti-P2RY12	Sigma	HPA014518	1:200	IF
Rabbit anti-TMEM119	Abcam	AB185333	1:200	IF
Rabbit anti-PLIN2	Proteintech	15294-1-AP	1:200	IF
Donkey Cy3 anti Goat	Jackson Laboratories	705-165-147	1:250	IF
Donkey Alexa Fluor 488 anti-Goat	Jackson Laboratories	705-545-147	1:250	IF
Donkey Alexa Fluor 647 anti-Goat	Jackson Laboratories	705-175-147	1:250	IF
Donkey Cy3 anti Rabbit	Jackson Laboratories	711-165-152	1:250	IF

Donkey Alexa Fluor 488 anti Rabbit	Jackson Laboratories	711-545-152	1:250	IF
Donkey Alexa Fluor 647 anti Rabbit	Jackson Laboratories	711-175-152	1:250	IF
Donkey Cy3 anti Mouse	Jackson Laboratories	715-165-151	1:250	IF
Goat Alexa Fluor 488 anti Mouse	Jackson Laboratories	715-545-151	1:250	IF
Donkey Alexa Fluor 647 anti Mouse	Jackson Laboratories	715-545-151	1:250	IF
Goat anti-rabbit HRP	Cell Signaling Technology	#7074	1:2000	WB
Goat anti-mouse HRP	Cell Signaling Technology	#7076	1:2000	WB
Hoechst	Thermo Scientific	62249	20mM	IF
DAPI	Thermo Scientific	62248	1 mg/mL	IF
CD64	Biolegend	#305014	0.6µg per 136µL reaction	FACS
CX3CR1	Biolegend	#341614	0.6µg per 136µL reaction	FACS
CD68	Biolegend	#333812	0.6µg per 136µL reaction	FACS
CD11b	Biolegend	#301306	0.6µg per 136µL reaction	FACS
HLA-DR	Biolegend	#307616	0.6µg per 136µL reaction	FACS
CD45	Biolegend	#304014	0.6µg per 136µL reaction	FACS
APC	Biolegend	#400122	100µg/mL	FACS
PCP-Cy5.5	Biolegend	#400632	200µg/mL	FACS

488-FITC	Biolegend	#400129	200μg/mL	FACS
PE	Biolegend	#400112	100μg/mL	FACS
PE-Cy7	Biolegend	#400232	100μg/mL	FACS
APC-Cy7	Biolegend	#400128	50μg/mL	FACS
Zombie Violet	Biolegend	#423114	1:1000	FACS
H3K27ac	Active Motif	#39685	1μg per IP	ChIP
MEF2C	Cell Signaling Technology	#5030	1μg per IP	ChIP

Reagents

	**Manufacturer**	**Catalog number**
Lysotracker Red DND-99	Invitrogen	L7528
Cell ROX Orange Reagent	Invitrogen	C10443
iNOS detection kit	abcam	ab211085
pHrodo Zymosan beads	Invitrogen	35364
Amyloid beta 1-42 (488-conjugated)	AnaSpec	AS-60479-01
Amyloid beta 1-42	AnaSpec	AS-20276
pHrodo Red	Invitrogen	P36600
Tau-441 fibrils	rPeptide	TF-1001-2
MSD Multi-Spot Assay System Human Proinflammatory Panel II	Mesoscale Discovery	K15053D
BODIPY 493/503	Invitrogen	D3922
Transwell permeable supports	Costar	3422
Paraformaldehyde	EMS	19210
Disuccinimidyl glutarate	CovaChem	13301
Tagment DNA Enzyme and Buffer Kit	Illumnia	20034197

ChIP DNA Clean and Concentrator Kit	Zymo Research	D5205
NEBNext High-Fidelity 2X PCR Mix	New England Biolabs	M0541
Dynabead Protein A beads	Invitrogen	10002D
Dynabead Protein G beads	Invitrogen	10004D
NEBNext Ultra II DNA Library Prep Kit for Illumina	New England Biolabs	E7645
SpeedBeads	Thermo Scientific Fisher	651520505025

References for Supplementary Methods

1. Gosselin, D., Skola, D., Coufal, N.G., Holtman, I.R., Schlachetzki, J.C.M., Sajti, E., Jaeger, B.N., O'Connor, C., Fitzpatrick, C., Pasillas, M.P., et al. (2017). An environment-dependent transcriptional network specifies human microglia identity. Science *356*. 10.1126/science.aal3222.
2. Firth, A.L., Dargitz, C.T., Qualls, S.J., Menon, T., Wright, R., Singer, O., Gage, F.H., Khanna, A., and Verma, I.M. (2014). Generation of multiciliated cells in functional airway epithelia from human induced pluripotent stem cells. Proc Natl Acad Sci U S A *111*, E1723-1730. 10.1073/pnas.1403470111.
3. Mohajeri, K., Yadav, R., D'haene, E., Boone, P.M., Erdin, S., Gao, D., Moyses-Oliveira, M., Bhavsar, R., Currall, B.B., O'Keefe, K., et al. (2022). Transcriptional and functional consequences of alterations to MEF2C and its topological organization in neuronal models. Am J Hum Genet *109*, 2049-2067. 10.1016/j.ajhg.2022.09.015.
4. Nott, A., Holtman, I.R., Coufal, N.G., Schlachetzki, J.C.M., Yu, M., Hu, R., Han, C.Z., Pena, M., Xiao, J., Wu, Y., et al. (2019). Brain cell type-specific enhancer-promoter interactome maps and disease. Science *366*, 1134-1139. 10.1126/science.aay0793.
5. Abud, E.M., Ramirez, R.N., Martinez, E.S., Healy, L.M., Nguyen, C.H.H., Newman, S.A., Yeromin, A.V., Scarfone, V.M., Marsh, S.E., Fimbres, C., et al. (2017). iPSC-Derived Human Microglia-like Cells to Study Neurological Diseases. Neuron *94*, 278-293.e279. 10.1016/j.neuron.2017.03.042.
6. McQuade, A., Coburn, M., Tu, C.H., Hasselmann, J., Davtyan, H., and Blurton-Jones, M. (2018). Development and validation of a simplified method to generate human microglia from pluripotent stem cells. Mol Neurodegener *13*, 67. 10.1186/s13024-018-0297-x.
7. Warden, A., Truitt, J., Merriman, M., Ponomareva, O., Jameson, K., Ferguson, L.B., Mayfield, R.D., and Harris, R.A. (2016). Localization of PPAR isotypes in the adult mouse and human brain. Sci Rep *6*, 27618. 10.1038/srep27618.
8. Han, C.Z., Li, R.Z., Hansen, E., Trescott, S., Fixsen, B.R., Nguyen, C.T., Mora, C.M., Spann, N.J., Bennett, H.R., Poirion, O., et al. (2023). Human microglia maturation is underpinned by specific gene regulatory networks. Immunity. 10.1016/j.immuni.2023.07.016.
9. Bohlen, C.J., Bennett, F.C., Tucker, A.F., Collins, H.Y., Mulinyawe, S.B., and Barres, B.A. (2017). Diverse Requirements for Microglial Survival, Specification, and Function

Revealed by Defined-Medium Cultures. Neuron *94*, 759-773.e758. 10.1016/j.neuron.2017.04.043.
10. Marschallinger, J., Iram, T., Zardeneta, M., Lee, S.E., Lehallier, B., Haney, M.S., Pluvinage, J.V., Mathur, V., Hahn, O., Morgens, D.W., et al. (2020). Lipid-droplet-accumulating microglia represent a dysfunctional and proinflammatory state in the aging brain. Nat Neurosci *23*, 194-208. 10.1038/s41593-019-0566-1.
11. Gandal, M.J., Zhang, P., Hadjimichael, E., Walker, R.L., Chen, C., Liu, S., Won, H., van Bakel, H., Varghese, M., Wang, Y., et al. (2018). Transcriptome-wide isoform-level dysregulation in ASD, schizophrenia, and bipolar disorder. Science *362*. 10.1126/science.aat8127.
12. Zhang, B., and Horvath, S. (2005). A general framework for weighted gene co-expression network analysis. Stat Appl Genet Mol Biol *4*, Article17. 10.2202/1544-6115.1128.
13. Zhou, Y., Zhou, B., Pache, L., Chang, M., Khodabakhshi, A.H., Tanaseichuk, O., Benner, C., and Chanda, S.K. (2019). Metascape provides a biologist-oriented resource for the analysis of systems-level datasets. Nat Commun *10*, 1523. 10.1038/s41467-019-09234-6.
14. Shannon, P., Markiel, A., Ozier, O., Baliga, N.S., Wang, J.T., Ramage, D., Amin, N., Schwikowski, B., and Ideker, T. (2003). Cytoscape: a software environment for integrated models of biomolecular interaction networks. Genome Res *13*, 2498-2504. 10.1101/gr.1239303.
15. Tsugawa, H., Cajka, T., Kind, T., Ma, Y., Higgins, B., Ikeda, K., Kanazawa, M., VanderGheynst, J., Fiehn, O., and Arita, M. (2015). MS-DIAL: data-independent MS/MS deconvolution for comprehensive metabolome analysis. Nat Methods *12*, 523-526. 10.1038/nmeth.3393.
16. Kind, T., Liu, K.H., Lee, D.Y., DeFelice, B., Meissen, J.K., and Fiehn, O. (2013). LipidBlast in silico tandem mass spectrometry database for lipid identification. Nat Methods *10*, 755-758. 10.1038/nmeth.2551.
17. Evans, C.G., Wisen, S., and Gestwicki, J.E. (2006). Heat shock proteins 70 and 90 inhibit early stages of amyloid beta-(1-42) aggregation in vitro. J Biol Chem *281*, 33182-33191. 10.1074/jbc.M606192200.
18. McQuade, A., Kang, Y.J., Hasselmann, J., Jairaman, A., Sotelo, A., Coburn, M., Shabestari, S.K., Chadarevian, J.P., Fote, G., Tu, C.H., et al. (2020). Gene expression and functional deficits underlie TREM2-knockout microglia responses in human models of Alzheimer's disease. Nat Commun *11*, 5370. 10.1038/s41467-020-19227-5.
19. Ramaswami, G., Won, H., Gandal, M.J., Haney, J., Wang, J.C., Wong, C.C.Y., Sun, W., Prabhakar, S., Mill, J., and Geschwind, D.H. (2020). Integrative genomics identifies a convergent molecular subtype that links epigenomic with transcriptomic differences in autism. Nat Commun *11*, 4873. 10.1038/s41467-020-18526-1.
20. Bulik-Sullivan, B.K., Loh, P.R., Finucane, H.K., Ripke, S., Yang, J., Schizophrenia Working Group of the Psychiatric Genomics, C., Patterson, N., Daly, M.J., Price, A.L., and Neale, B.M. (2015). LD Score regression distinguishes confounding from polygenicity in genome-wide association studies. Nat Genet *47*, 291-295. 10.1038/ng.3211.
21. Grove, J., Ripke, S., Als, T.D., Mattheisen, M., Walters, R.K., Won, H., Pallesen, J., Agerbo, E., Andreassen, O.A., Anney, R., et al. (2019). Identification of common genetic risk variants for autism spectrum disorder. Nat Genet *51*, 431-444. 10.1038/s41588-019-0344-8.
22. Pedersen, E.M., Agerbo, E., Plana-Ripoll, O., Steinbach, J., Krebs, M.D., Hougaard, D.M., Werge, T., Nordentoft, M., Borglum, A.D., Musliner, K.L., et al. (2023). ADuLT: An

efficient and robust time-to-event GWAS. Nat Commun *14*, 5553. 10.1038/s41467-023-41210-z.
23. Jansen, I.E., Savage, J.E., Watanabe, K., Bryois, J., Williams, D.M., Steinberg, S., Sealock, J., Karlsson, I.K., Hagg, S., Athanasiu, L., et al. (2019). Genome-wide meta-analysis identifies new loci and functional pathways influencing Alzheimer's disease risk. Nat Genet *51*, 404-413. 10.1038/s41588-018-0311-9.
24. Schizophrenia Working Group of the Psychiatric Genomics, C. (2014). Biological insights from 108 schizophrenia-associated genetic loci. Nature *511*, 421-427. 10.1038/nature13595.
25. Stahl, E.A., Breen, G., Forstner, A.J., McQuillin, A., Ripke, S., Trubetskoy, V., Mattheisen, M., Wang, Y., Coleman, J.R.I., Gaspar, H.A., et al. (2019). Genome-wide association study identifies 30 loci associated with bipolar disorder. Nat Genet *51*, 793-803. 10.1038/s41588-019-0397-8.
26. Levey, D.F., Gelernter, J., Polimanti, R., Zhou, H., Cheng, Z., Aslan, M., Quaden, R., Concato, J., Radhakrishnan, K., Bryois, J., et al. (2020). Reproducible Genetic Risk Loci for Anxiety: Results From approximately 200,000 Participants in the Million Veteran Program. Am J Psychiatry *177*, 223-232. 10.1176/appi.ajp.2019.19030256.
27. Finucane, H.K., Bulik-Sullivan, B., Gusev, A., Trynka, G., Reshef, Y., Loh, P.R., Anttila, V., Xu, H., Zang, C., Farh, K., et al. (2015). Partitioning heritability by functional annotation using genome-wide association summary statistics. Nat Genet *47*, 1228-1235. 10.1038/ng.3404.
28. Mancuso, R., Van Den Daele, J., Fattorelli, N., Wolfs, L., Balusu, S., Burton, O., Liston, A., Sierksma, A., Fourne, Y., Poovathingal, S., et al. (2019). Stem-cell-derived human microglia transplanted in mouse brain to study human disease. Nat Neurosci *22*, 2111-2116. 10.1038/s41593-019-0525-x.
29. Kiani Shabestari, S., Morabito, S., Danhash, E.P., McQuade, A., Sanchez, J.R., Miyoshi, E., Chadarevian, J.P., Claes, C., Coburn, M.A., Hasselmann, J., et al. (2022). Absence of microglia promotes diverse pathologies and early lethality in Alzheimer's disease mice. Cell Rep *39*, 110961. 10.1016/j.celrep.2022.110961.
30. Hasselmann, J., Coburn, M.A., England, W., Figueroa Velez, D.X., Kiani Shabestari, S., Tu, C.H., McQuade, A., Kolahdouzan, M., Echeverria, K., Claes, C., et al. (2019). Development of a Chimeric Model to Study and Manipulate Human Microglia In Vivo. Neuron *103*, 1016-1033.e1010. 10.1016/j.neuron.2019.07.002.
31. Young, K., and Morrison, H. (2018). Quantifying Microglia Morphology from Photomicrographs of Immunohistochemistry Prepared Tissue Using ImageJ. J Vis Exp. 10.3791/57648.
32. Arganda-Carreras, I., Fernández-González, R., Muñoz-Barrutia, A., and Ortiz-De-Solorzano, C. (2010). 3D reconstruction of histological sections: Application to mammary gland tissue. Microsc Res Tech *73*, 1019-1029. 10.1002/jemt.20829.
33. Karperien, A. (2013). FracLac for ImageJ. http://rsb.info.nih.gov/ij/plugins/fraclac/FLHelp/Introduction.htm . 1999-2013.
34. Nahirney, P.C., and Tremblay, M.E. (2021). Brain Ultrastructure: Putting the Pieces Together. Front Cell Dev Biol *9*, 629503. 10.3389/fcell.2021.629503.
35. Peters, A., Josephson, K., and Vincent, S.L. (1991). Effects of aging on the neuroglial cells and pericytes within area 17 of the rhesus monkey cerebral cortex. Anat Rec *229*, 384-398. 10.1002/ar.1092290311.
