# Supplementary material for: Transcriptional and epigenetic targets of MEF2C in human microglia contribute to cellular functions related to autism risk and age-related disease
Source: Nat Immunol. 2025 Oct 22;26(11):1989–2003. doi: 10.1038/s41590-025-02299-0 (PMC12571900; doi:10.1038/s41590-025-02299-0)
Supplement: Supplementary file 2 — Reporting Summary [file 41590_2025_2299_MOESM2_ESM.pdf]

nature portfolio

Corresponding author(s): Nicole Coufal & Christopher Glass

Last updated by author(s): Sep 2, 2025

Reporting Summary

Nature Portfolio wishes to improve the reproducibility of the work that we publish. This form provides structure for consistency and transparency in reporting. For further information on Nature Portfolio policies, see our Editorial Policies and the Editorial Policy Checklist.

Statistics

For all statistical analyses, confirm that the following items are present in the figure legend, table legend, main text, or Methods section.

n/a	Confirmed	
☐	☒	The exact sample size (*n*) for each experimental group/condition, given as a discrete number and unit of measurement
☐	☒	A statement on whether measurements were taken from distinct samples or whether the same sample was measured repeatedly
☐	☒	The statistical test(s) used AND whether they are one- or two-sided<br>*Only common tests should be described solely by name; describe more complex techniques in the Methods section.*
☐	☒	A description of all covariates tested
☐	☒	A description of any assumptions or corrections, such as tests of normality and adjustment for multiple comparisons
☐	☒	A full description of the statistical parameters including central tendency (e.g. means) or other basic estimates (e.g. regression coefficient) AND variation (e.g. standard deviation) or associated estimates of uncertainty (e.g. confidence intervals)
☐	☒	For null hypothesis testing, the test statistic (e.g. *F*, *t*, *r*) with confidence intervals, effect sizes, degrees of freedom and *P* value noted<br>*Give P values as exact values whenever suitable.*
☒	☐	For Bayesian analysis, information on the choice of priors and Markov chain Monte Carlo settings
☐	☒	For hierarchical and complex designs, identification of the appropriate level for tests and full reporting of outcomes
☒	☐	Estimates of effect sizes (e.g. Cohen's *d*, Pearson's *r*), indicating how they were calculated

Our web collection on statistics for biologists contains articles on many of the points above.

Software and code

Policy information about availability of computer code

Data collection

Incucyte S3 live imaging software v2022B (Sartorius Bioanalytical Instruments Inc).

Data analysis

Software: Graphpad Prism 10. and R studio version 1.3.1056.

Data preprocessing
FASTQ-files were mapped to the UCSC genome build hg38 with STAR (v2.7.9a) using default parameters and converted to HOMER tag directories. The function “analyzeRepeats” was used to quantify raw reads and then normalize reads as transcripts per million (TPM). A pseudocount of 1 TPM was added to each gene before base-2 logarithm transformation of TPM for each gene. ATAC-seq FASTQ files were trimmed prior to mapping with Bowtie 2 (2.3.5.1); ATAC-seq files were trimmed to 30 bp. Following trimming, ATAC-seq FASTQ files were mapped using Bowtie 2. After mapping, tag directories were created using the HOMER (v4.11.1) command makeTagDirectory.

RNA analysis
Differential expression was performed using R (v4.0.4) and the package DESeq2 (v1.30.1) with an FDR<0.05 and log2 Fold Change > 1. In addition to pairwise comparisons, a linear model was set up to compare Control microglia, MHS microglia and KO microglia using the R packages (DEGreport (v1.26.0), DEseq2). For gene set comparisons from RNA-seq library preparation, we generated lists for the published datasets by using a fold change cutoff and compared these lists with the top upregulated and downregulated genes in MEF2C Control and KO microglia. For PsychEncode enrichment analysis, we pulled the WGCNA module information from PsychEncode and performed Fisher’s exact tests to determine gene set overlap odds ratio and significance with an adjusted -log10 P-value < 0.05.

Weighted gene co-expression network analysis (WGCNA), using filtered log2 transformed expression data for Control MEF2C microglia and KO microglia. This tool was then applied to construct scale-free networks that specify coordinately regulated genes (i.e., modules). To explore the

modular structures of the co-expression network, the adjacency matrix was transformed into a topological overlap matrix. Because topological overlap between two genes reflects both their direct and indirect interactions with all other genes in the network, this approach helps create more cohesive and biologically meaningful data interpretation. WGCNA parameters optimized including a minimum module size equal to 75 genes and a 0.99 tree-cut height. The maximal statistical significance for gene set enrichment was determined using a Fishers-exact test, corrected for multiple comparisons using a Benjamini-Hochberg false discovery rate (FDR) < 0.05. Each module was assigned a unique, arbitrary color and number. Individual gene sets from modules and differentially expressed genes were functionally annotated for biological processes and pathway enrichment using Metascape and selected if they passed an adjusted P-value of P < 0.05. Select gene sets, based upon statistical and biological significance, were assembled to visualize network properties using the bioinformatics tool Cytoscape (v3.8.1).

IDR analysis of ChIP and ATAC peaks
Peaks were called with HOMER for each tag directory with relaxed peak finding parameters "-L 0 -C 0 -fdr 0.9". ATAC peaks were called with additional parameters "-minDist 200 -size 200". HOMER was used to call peaks from the ATAC-seq mapped tag directories and only ATAC peaks with an Irreproducible Discovery Rate (IDR) < 0.05 was used for downstream analysis. Peak lists from MEF2C CTRL, HET and KO iMG ATAC were merged with HOMER's mergePeaks and annotated for H3K27ac reads with HOMER's annotatePeaks. DESeq2 was used to identify ATAC peaks that are differentially acetylated between MEF2C CTRL and KO iMG groups. The peaks with increased acetylation in MEF2C CTRL vs KO were overlapped with MEF2C peaks to identify direct and indirect MEF2C activation regions. The peaks with increased acetylation in MEF2C KO vs CTRL were overlapped with MEF2C peaks to identify direct and indirect MEF2C repression regions. HOMER's findMotifsGenome.pl was used to identify default and de novo motifs from the final peak lists for MEF2C direct and indirect activation and repression sites. The background peaks used were the random genome sequences generated by HOMER. For cell type-specific mapping of ASD distal H3K27ac peaks from publicly available differential peak information was pulled from Ramaswami et al as well as cell-type specific promoter and distal enhancer genomic locations from Nott et al. Overlapping genomic ranges was determined by the package GenomicRanges (v.1.54.1) and plotted as the total number of cell type specific overlapping peaks for promoters and distal enhancers. To determine the peaks overlap between our H3K27ac at distal ATAC peaks and distal ASD peaks, first we lifted over the hg19 genomic ranges from Ramaswami56 using Ensembl Assembly Converter (v.111). Next, we determined the number of overlapping genomic ranges with our dataset using GenomicRanges (v.1.54.1) and pypher (v.0.7.1) for hypergeometric p-value significance of overlap.

ATAC-seq and ChIP-seq analysis
To quantify the TF binding and chromatin accessibility between conditions, raw and normalized tag counts at merged IDR peaks identified by HOMER's mergePeaks were identified using HOMER's annotatePeaks with "-noadj," "-size 500" for TF ChIP-seq peaks and "-size 1000" for ATAC peaks annotated with H3K27ac reads. DESeq2 was used to identify differentially bound TF binding distal sites or differential distal chromatin accessibility (p-adj. < 0.05 and FC >2 or <-2).

Motif Analysis
To identify motifs enriched in peak regions over the background, HOMER's motif analysis (findMotifsGenome.pl) including known default motifs and de novo motifs was used. The background peaks used random genome sequences generated automatically by HOMER. Statistical Analyses. Gene expression differences and differential TF binding/H3K27ac signal was calculated with DESeq2 with Benjamini-Hochberg multiple testing correction. Genes and peaks were considered differential at FC >1 or <-1, p.adj < 0.05. Significance of gene set overlap was calculated using the Fisher exact test, p.value < 0.05.

Linkage disequilibrium analysis (LDSC)
European ancestry LD scores and weights were obtained from the 1000 Genomes Phase 3 SNPs as a reference panel, while summary statistics were derived from GWAS studies conducted on autism, Alzheimer's, and neuropsychiatric disorders. Annotated SNP lists were created for all SNPs within ±5 kb of ATAC-seq or ChIP-seq peaks of interest, corresponding to the regions where heritability enrichment was being assessed. The Bulik-Sullivan LDSC package was adapted to carry out the calculations, following the recommended published LDSC baseline model.

For manuscripts utilizing custom algorithms or software that are central to the research but not yet described in published literature, software must be made available to editors and reviewers. We strongly encourage code deposition in a community repository (e.g. GitHub). See the Nature Portfolio guidelines for submitting code & software for further information.

Data

Policy information about availability of data

All manuscripts must include a data availability statement. This statement should provide the following information, where applicable:

- Accession codes, unique identifiers, or web links for publicly available datasets
- A description of any restrictions on data availability
- For clinical datasets or third party data, please ensure that the statement adheres to our policy

Previously reported data are available from GEO include Gosselin et al.31: GSE62826, Han et 521 al.37: GSE226690. PLAC-seq data is available on dbGAP for Nott et al30 on dbGAP: 522 phs001373.v2.p2. Data generated from this study are accessible under the SuperSeries GSE306993 and will be released on publication.

Research involving human participants, their data, or biological material

Policy information about studies with human participants or human data. See also policy information about sex, gender (identity/presentation), and sexual orientation and race, ethnicity and racism.

Reporting on sex and gender	We did not consider sex and gender in this study. However, it was noted that both postnatal tissue were from male donors. Meanwhile this information is unknown for the fetal sample.
Reporting on race, ethnicity, or	We did not consider race or ethnicity in this study and therefore do not report on these variables.

other socially relevant groupings	
Population characteristics	Fetal tissue = Gestational Week 17, sex unknown; Postnatal tissue = 17 years and 20 years old, both male
Recruitment	Brain tissue was obtained with informed consent from adult patients, or by informed parental consent and assent when applicable from pediatric patients
Ethics oversight	UC San Diego and Rady Children's Hospital Institutional Review Board (UCSD IRB 171361)

Note that full information on the approval of the study protocol must also be provided in the manuscript.

Field-specific reporting

Please select the one below that is the best fit for your research. If you are not sure, read the appropriate sections before making your selection.

☒ Life sciences ☐ Behavioural & social sciences ☐ Ecological, evolutionary & environmental sciences

For a reference copy of the document with all sections, see nature.com/documents/nr-reporting-summary-flat.pdf

Life sciences study design

All studies must disclose on these points even when the disclosure is negative.

Sample size	Three independent cell lines generated per genotype (MEF2C KO and MHS/heterozygous) with different CRISPR guides targeting alternate parts of the MEF2C gene were utilized, with CRISPR guide matched controls selected from the unedited but transfected matched cell populations, which was paired with a previously published second cell line MEF2C KO and isogenic control. Each experiment was replicated independently utilizing multiple lines/genotype. For all experiments, no statistical methods were used to pre-determine sample size but our sample sizes are similar to those reported in previous publications (Nott et al 2019, McQuade et al 2020).
Data exclusions	The reported data sets are from sequential samples for which cell viability and sequencing libraries met technical quality standards. No other criteria were used to include or exclude samples.
Replication	For RNA-seq studies, 6-8 biologically independent samples per group were used. For ATAC-seq, H3K27ac ChIP-seq and MEF2C ChIP-seq 2-4 biologically independent samples were used. All assays were successfully replicated 2-3 times; quantification and statistics are run on combined replicate experiments. Only findings that were independently replicated were included within the manuscript.
Randomization	Cell seeding in plates and slides and pup engraftments were performed in a random order to implement randomization. Our study utilizes isogenic lines to eliminate the need to account for additional covariates.
Blinding	For the in vivo xenotransplantation quantitation, the individual performing the analysis was blinded as to the genotype of the xenotransplanted microglia. For in vitro studies, blinding was not performed since the genotype could easily be determined from cellular morphology. Incucyte experiments were batch-processed by software; the same parameters were applied across all replicates.

Reporting for specific materials, systems and methods

We require information from authors about some types of materials, experimental systems and methods used in many studies. Here, indicate whether each material, system or method listed is relevant to your study. If you are not sure if a list item applies to your research, read the appropriate section before selecting a response.

Materials & experimental systems

n/a	Involved in the study	
☐	☒	Antibodies
☐	☒	Eukaryotic cell lines
☒	☐	Palaeontology and archaeology
☐	☒	Animals and other organisms
☒	☐	Clinical data
☒	☐	Dual use research of concern
☒	☐	Plants

Methods

n/a	Involved in the study	
☐	☒	ChIP-seq
☐	☒	Flow cytometry
☒	☐	MRI-based neuroimaging

Antibodies

Antibodies used

Antibody Manufacturer Catalog # Dilution Application
Rabbit anti-MEF2C Abcam ab211493 1:200, 1:1000 IF, WB
Goat anti-IBA1 Abcam ab5076 1:200 IF
Rabbit anti-IBA1 Wako 019-19741 1:500/1:1000 IF/EM
Rat anti- CTIP Abcam ab18465 1:200 IF

Mouse anti-CD68 Dako M0814 1:200 IF
Mouse anti-LAMP1 Invitrogen 14-1079-80 1:200 IF
Rat anti-LAMP2 Abcam ab13524 1:250 IF
Goat anti-B-gal Biogenesis 103006 1:250 IF
Goat anti-TREM2 R&D system AF1828 1:100 IF
Rabbit anti-APOE Invitrogen 701241 1:100 IF
Rabbit anti-Ku80 Abcam ab80592 1:100 IF
Rabbit anti-pSTAT1 Cell Signaling Technology #8826 1:1000 WB
Rabbit anti-STAT1 Cell Signaling Technology #14994 1:1000 WB
Mouse anti-GAPDH Santa Cruz Biotech. Sc-47724 1:5000 WB
Rabbit anti-P2RY12 Sigma HPA014518 1:200 IF
Rabbit anti-TMEM119 Abcam AB185333 1:200 IF
Rabbit anti-PLIN2 Proteintech 15294-1-AP 1:200 IF
Donkey Cy3 anti Goat Jackson Laboratories 705-165-147 1:250 IF
Donkey Alexa Fluor 488 anti-Goat Jackson Laboratories 705-545-147 1:250 IF
Donkey Alexa Fluor 647 anti-Goat Jackson Laboratories 705-175-147 1:250 IF
Donkey Cy3 anti Rabbit Jackson Laboratories 711-165-152 1:250 IF
Donkey Alexa Fluor 488 anti Rabbit Jackson Laboratories 711-545-152 1:250 IF
Donkey Alexa Fluor 647 anti Rabbit Jackson Laboratories 711-175-152 1:250 IF
Donkey Cy3 anti Mouse Jackson Laboratories 715-165-151 1:250 IF
Goat Alexa Fluor 488 anti Mouse Jackson Laboratories 715-545-151 1:250 IF
Donkey Alexa Fluor 647 anti Mouse Jackson Laboratories 715-545-151 1:250 IF
Goat anti-rabbit HRP Cell Signaling Technology #7074 1:2000 WB
Goat anti-mouse HRP Cell Signaling Technology #7076 1:2000 WB
Hoechst Thermo Scientific 62249 20mM IF
DAPI Thermo Scientific 62248 1 mg/mL IF
CD64 Biolegend #305014 0.6μg per 136μL reaction FACS
CX3CR1 Biolegend #341614 0.6μg per 136μL reaction FACS
CD68 Biolegend #333812 0.6μg per 136μL reaction FACS
CD11b Biolegend #301306 0.6μg per 136μL reaction FACS
HLA-DR Biolegend #307616 0.6μg per 136μL reaction FACS
CD45 Biolegend #304014 0.6μg per 136μL reaction FACS
APC Biolegend #400122 100μg/mL FACS
PCP-Cy5.5 Biolegend #400632 200μg/mL FACS
488-FITC Biolegend #400129 200μg/mL FACS
PE Biolegend #400112 100μg/mL FACS
PE-Cy7 Biolegend #400232 100μg/mL FACS
APC-Cy7 Biolegend #400128 50μg/mL FACS
Zombie Violet Biolegend #423114 1:1000 FACS
H3K27ac Active Motif #39685 1μg per IP ChIP
MEF2C Cell Signaling Technology #5030 1μg per IP ChIP

Validation: Antibodies were validated by the manufacturers listed above.

Eukaryotic cell lines

Policy information about cell lines and Sex and Gender in Research

Cell line source(s): Two cell lines were utilized in this study, the EC11 cell line (with references, extensively published) and the Coriell GM08330 cell line. Both lines are XY by karyotyping.

Authentication: All cell lines were karyotyped and verified to express pluripotency markers and to be free of mycoplasma contamination.

Mycoplasma contamination: All cell lines were regularly mycoplasma tested and were negative.

Commonly misidentified lines (See ICLAC register): N/A

Animals and other research organisms

Policy information about studies involving animals; ARRIVE guidelines recommended for reporting animal research, and Sex and Gender in Research

Laboratory animals: JAX strain 017708 was utilized in these studies.

Wild animals: N/A

Reporting on sex: Male mice were utilized, for a sex-matched xenotransplantation given both iPSC cell lines were also male.

Field-collected samples: N/A

Ethics oversight: All animal procedures described have been approved by UCSD IACUC at an AAALAC accredited facility.

Note that full information on the approval of the study protocol must also be provided in the manuscript.

Plants

Seed stocks	*Report on the source of all seed stocks or other plant material used. If applicable, state the seed stock centre and catalogue number. If plant specimens were collected from the field, describe the collection location, date and sampling procedures.*
Novel plant genotypes	*Describe the methods by which all novel plant genotypes were produced. This includes those generated by transgenic approaches, gene editing, chemical/radiation-based mutagenesis and hybridization. For transgenic lines, describe the transformation method, the number of independent lines analyzed and the generation upon which experiments were performed. For gene-edited lines, describe the editor used, the endogenous sequence targeted for editing, the targeting guide RNA sequence (if applicable) and how the editor was applied.*
Authentication	*Describe any authentication procedures for each seed stock used or novel genotype generated. Describe any experiments used to assess the effect of a mutation and, where applicable, how potential secondary effects (e.g. second site T-DNA insertions, mosiacism, off-target gene editing) were examined.*

ChIP-seq

Data deposition

☒ Confirm that both raw and final processed data have been deposited in a public database such as GEO.

☒ Confirm that you have deposited or provided access to graph files (e.g. BED files) for the called peaks.

Data access links
May remain private before publication.

Previously reported data are available from GEO include Gosselin et al.24: GSE62826, Han et al.5: GSE226690. PLAC-seq data is available on dbGAP for Nott et al23 on dbGAP: phs001373.v2.p2. Data generated from this study are accessible under the SuperSeries GSE306993.

Files in database submission

human_iMs_RNA_polyA_Control_MEF2C_42N_CZH_l20200513_ATTCCT_S19_L002_R1_001.fastq.gz
human_iMs_RNA_polyA_Control_MEF2C_42N_CZH_s20190307_ACTTGA_S28_L002_R1_001
human_iMs_RNA_polyA_Control_MEF2C_71N_CZH_l20200513_GTTTCG_S8_L001_R1_001.fastq.gz
human_iMs_RNA_polyA_Control_MEF2C_71N_CZH_s20190307_ATGTCA_S29_L002_R1_001.fastq.gz
human_iMs_RNA_polyA_Control_MEF2C_D4N_CZH_l20200513_CAAAAG_S20_L002_R1_001.fastq.gz
human_iMs_RNA_polyA_Control_MEF2C_D4N_CZH_s20190307_CCGTCC_S30_L002_R1_001.fastq.gz
human_iMs_RNA_polyA_Het_MEF2C_237_CZH_s20190307_GTGGCC_S34_L002_R1_001.fastq.gz
human_iMs_RNA_polyA_Het_MEF2C_2H11_CZH_l20200513_CGTACG_S18_L002_R1_001.fastq.gz
human_iMs_RNA_polyA_Het_MEF2C_2H11_CZH_s20180828_CGGAAT_S31_L002_R1_001.fastq.gz
human_iMs_RNA_polyA_Het_MEF2C_2H11_CZH_s20190307_GTCCGC_S32_L002_R1_001.fastq.gz
human_iMs_RNA_polyA_Het_MEF2C_2H9_CZH_l20200513_GAGTGG_S9_L001_R1_001.fastq.gz
human_iMs_RNA_polyA_Het_MEF2C_2H9_CZH_s20190307_GTGAAA_S33_L002_R1_001.fastq.gz
human_iMs_RNA_polyA_Het_MEF2C_rep237_CZH_l20200513_ACTGAT_S11_L001_R1_001.fastq.gz
human_iMs_RNA_polyA_Homo_MEF2C_1F37_CZH_s20180828_ATTCCT_S37_L002_R1_001.fastq.gz
human_iMs_RNA_polyA_Homo_MEF2C_1F37_CZH_s20190307_CAAAAG_S38_L002_R1_001.fastq.gz
human_iMs_RNA_polyA_Homo_MEF2C_1G4317_CZH_s20180828_ACTGAT_S35_L002_R1_001.fastq.gz
human_iMs_RNA_polyA_Homo_MEF2C_1G4317_CZH_s20190307_ATGAGC_S36_L002_R1_001.fastq.gz
human_iMs_RNA_polyA_Homo_MEF2C_2H10131_CZH_s20180828_CAACTA_S39_L002_R1_001.fastq.gz
human_iMs_RNA_polyA_Homo_MEF2C_2H10131_CZH_s20190307_CACCGG_S40_L002_R1_001.fastq.gz
human_iMs_RNA_polyA_KO_MEF2C_1F37_CZH_l20200513_GGTAGC_S10_L001_R1_001.fastq.gz
human_iMs_RNA_polyA_KO_MEF2C_2H10_CZH_l20200513_ATGAGC_S12_L001_R1_001.fastq.gz
HOMER.rawCount.txt
HOMER.rawTPM.txt
human_Monocytesinvitro1_ATAC_JINGW_l20240710_GCTCATGA_S37_L006_R1_001.trim.fastq.gz
human_Monocytesinvitro1_ATAC_JINGW_l20240710_GCTCATGA_S37_L006_R2_001.trim.fastq.gz
human_Monocytesinvitro2_ATAC_JINGW_l20240710_ATGCGCAG_S44_L006_R1_001.trim.fastq.gz
human_Monocytesinvitro2_ATAC_JINGW_l20240710_ATGCGCAG_S44_L006_R2_001.trim.fastq.gz
human_Monocytesinvitro3_ATAC_JINGW_l20240710_TAGCGCTC_S45_L006_R1_001.trim.fastq.gz
human_Monocytesinvitro3_ATAC_JINGW_l20240710_TAGCGCTC_S45_L006_R2_001.trim.fastq.gz
human_monocytesrep1_RNA_JINGW_l20240209_TTCTGGTG_CCAGTGGT_S38_L003_R1_001.fastq.gz
human_monocytesrep1_RNA_JINGW_l20240209_TTCTGGTG_CCAGTGGT_S38_L003_R2_001.fastq.gz
human_monocytesrep2_RNA_JINGW_l20240209_CTACGAAG_CTCGACAG_S39_L003_R1_001.fastq.gz
human_monocytesrep2_RNA_JINGW_l20240209_CTACGAAG_CTCGACAG_S39_L003_R2_001.fastq.gz
human_monocytesrep3_RNA_JINGW_l20240209_CTTAATAC_CCATTGTG_S40_L003_R1_001.fastq.gz
human_monocytesrep3_RNA_JINGW_l20240209_CTTAATAC_CCATTGTG_S40_L003_R2_001.fastq.gz
human_monocytesrep4_RNA_JINGW_l20240209_CTTATGAA_GCCAATGT_S41_L003_R1_001.fastq.gz
human_monocytesrep4_RNA_JINGW_l20240209_CTTATGAA_GCCAATGT_S41_L003_R2_001.fastq.gz
tpm_monocytes_rep1_RNA.txt
tpm_monocytes_rep2_RNA.txt
tpm_monocytes_rep3_RNA.txt
tpm_monocytes_rep4_RNA.txt
monocytes_invitro1.txt
monocytes_invitro2.txt
monocytes_invitro3.txt
human_iMG_ATAC_237_MEF2CHet_rep1_CZH_l20210409_TTGACCCT_S18_L001_R1_001.trim.fastq.gz

human_iMG_ATAC_237_MEF2CHet_rep2_CZH_l20210409_CACGTCGA_S19_L001_R1_001.trim.fastq.gz
human_iMG_ATAC_2H10_MEF2CKO_rep1_CZH_l20210409_AAGAGGCA_S14_L001_R1_001.trim.fastq.gz
human_iMG_ATAC_2H10_MEF2CKO_rep2_CZH_l20210409_AGGTTGGG_S15_L001_R1_001.trim.fastq.gz
human_iMG_ATAC_2H11_MEF2CHet_rep1_CZH_l20210409_CCTCCACG_S20_L001_R1_001.trim.fastq.gz
human_iMG_ATAC_2H11_MEF2CHet_rep2_CZH_l20210409_AAGGAACG_S21_L001_R1_001.trim.fastq.gz
human_iMG_ATAC_42N_MEF2CCtrl_rep1_CZH_l20210409_CGTACTAG_S10_L001_R1_001.trim.fastq.gz
human_iMG_ATAC_42N_MEF2CCtrl_rep2_CZH_l20210409_TCCTGAGC_S11_L001_R1_001.trim.fastq.gz
human_iMG_ATAC_71N_MEF2CCtrl_rep1_CZH_l20210409_GGACTCCT_S12_L001_R1_001.trim.fastq.gz
human_iMG_ATAC_71N_MEF2CCtrl_rep2_CZH_l20210409_CTCTCTAC_S13_L001_R1_001.trim.fastq.gz
human_iMG_ATAC_IG4_MEF2CKO_rep1_CZH_l20210409_GTGTGGTG_S16_L001_R1_001.trim.fastq.gz
human_iMG_ATAC_IG4_MEF2CKO_rep2_CZH_l20210409_TGGGTTTC_S17_L001_R1_001.trim.fastq.gz
ATAC_Control_42N_rep1.txt
ATAC_Control_42N_rep2.txt
ATAC_Control_71N_rep1.txt
ATAC_Control_71N_rep2.txt
ATAC_Het_237_rep1.txt
ATAC_Het_237_rep2.txt
ATAC_Het_2H11_rep1.txt
ATAC_Het_2H11_rep2.txt
ATAC_KO_2H10_rep1.txt
ATAC_KO_2H10_rep2.txt
ATAC_KO_IG4_rep1.txt
ATAC_KO_IG4_rep2.txt
human_CTRL42N_iMGs_ChIP_MEF2C_CTN_l20240905_CATGATGA_GTTAGAGG_S8_L007_R1_001.fastq.gz
human_CTRL42N_iMGs_ChIP_MEF2C_CTN_l20240905_CATGATGA_GTTAGAGG_S8_L007_R2_001.fastq.gz
human_CTRLD4N_iMGs_ChIP_MEF2C_CTN_l20240905_CAATGATG_CACGGACG_S7_L007_R1_001.fastq.gz
human_CTRLD4N_iMGs_ChIP_MEF2C_CTN_l20240905_CAATGATG_CACGGACG_S7_L007_R2_001.fastq.gz
human_HET237_iMGs_ChIP_MEF2C_CTN_l20240905_CAGACCAC_GCTTCGGC_S9_L007_R1_001.fastq.gz
human_HET237_iMGs_ChIP_MEF2C_CTN_l20240905_CAGACCAC_GCTTCGGC_S9_L007_R2_001.fastq.gz
human_HET2H11_iMGs_ChIP_MEF2C_CTN_l20240905_CTGGAAGC_GGTGCAGA_S10_L007_R1_001.fastq.gz
human_HET2H11_iMGs_ChIP_MEF2C_CTN_l20240905_CTGGAAGC_GGTGCAGA_S10_L007_R2_001.fastq.gz
human_iMG_ChIP_H3K27ac_237_Het_rep1_CZH_s20210820_TGCTGAGG_CGCAACGT_S33_L002_R1_001.fastq.gz
human_iMG_ChIP_H3K27ac_237_Het_rep2_CZH_s20211008_TTATCAAC_CGAGTACG_S40_L002_R1_001.fastq.gz
human_iMG_ChIP_H3K27ac_2H10_KO_rep1_CZH_s20210820_GGATAATA_TGGTACTA_S31_L002_R1_001.fastq.gz
human_iMG_ChIP_H3K27ac_2H10_KO_rep2_CZH_s20210820_TGAAGAAT_GAAGACAG_S38_L002_R1_001.fastq.gz
human_iMG_ChIP_H3K27ac_2H11_Het_rep1_CZH_s20210820_ACCATTAA_GAACCGGT_S30_L002_R1_001.fastq.gz
human_iMG_ChIP_H3K27ac_2H11_Het_rep2_CZH_s20210820_TTAGGTTG_GACGATTG_S37_L002_R1_001.fastq.gz
human_iMG_ChIP_H3K27ac_42N_Control_rep1_CZH_s20210820_ACCGAGGT_CTCGTGTC_S29_L002_R1_001.fastq.gz
human_iMG_ChIP_H3K27ac_42N_Control_rep2_CZH_s20210820_ACCATATC_TACACGTT_S36_L002_R1_001.fastq.gz
human_iMG_ChIP_H3K27ac_71N_Control_rep1_CZH_s20211008_CTCTTCGT_CCGTCTAA_S34_L002_R1_001.fastq.gz
human_iMG_ChIP_H3K27ac_71N_Control_rep2_CZH_s20210820_GTTCTACT_GTAGCCAC_S35_L002_R1_001.fastq.gz
human_iMG_ChIP_H3K27ac_IG4_KO_rep1_CZH_s20210820_ACGTCCAT_GTGGCGAA_S32_L002_R1_001.fastq.gz
human_iMG_ChIP_H3K27ac_IG4_KO_rep2_CZH_s20210820_ACTCTACG_CCAGCTGA_S39_L002_R1_001.fastq.gz
human_iMG_ChIP_input_237_Het_rep1_CZH_s20210820_CACAGTAA_CACTAGAG_S46_L002_R1_001.fastq.gz
human_iMG_ChIP_input_237_Het_rep2_CZH_s20211008_CAGACCAC_GCTTCGGC_S53_L002_R1_001.fastq.gz
human_iMG_ChIP_input_2H10_KO_rep1_CZH_s20210820_CGATTATC_GAGTTGAT_S44_L002_R1_001.fastq.gz
human_iMG_ChIP_input_2H10_KO_rep2_CZH_s20210820_CAATGATG_CACGGACG_S51_L002_R1_001.fastq.gz
human_iMG_ChIP_input_2H11_Het_rep1_CZH_s20210820_GAATGCTC_GTCTAACA_S43_L002_R1_001.fastq.gz
human_iMG_ChIP_input_2H11_Het_rep2_CZH_s20210820_TGAGTCAG_CCGAACTT_S50_L002_R1_001.fastq.gz
human_iMG_ChIP_input_42N_Control_rep1_CZH_s20210820_CGTATTGG_GGTATCTT_S42_L002_R1_001.fastq.gz
human_iMG_ChIP_input_42N_Control_rep2_CZH_s20210820_GAACTTAT_AGATACGC_S49_L002_R1_001.fastq.gz
human_iMG_ChIP_input_71N_Control_rep1_CZH_s20210820_CGAAGGAC_GTTGACGC_S41_L002_R1_001.fastq.gz
human_iMG_ChIP_input_71N_Control_rep2_CZH_s20210820_GATCAACA_CTATGTTA_S48_L002_R1_001.fastq.gz
human_iMG_ChIP_input_IG4_KO_rep1_CZH_s20210820_CGGTGGTA_GCCTAGTA_S45_L002_R1_001.fastq.gz
human_iMG_ChIP_input_IG4_KO_rep2_CZH_s20210820_CATGATGA_GTTAGAGG_S52_L002_R1_001.fastq.gz
human_KO1F37_iMGs_ChIP_MEF2C_CTN_l20240905_CAACCGTG_CTCTCACG_S11_L007_R1_001.fastq.gz
human_KO1F37_iMGs_ChIP_MEF2C_CTN_l20240905_CAACCGTG_CTCTCACG_S11_L007_R2_001.fastq.gz
human_KO2H10_iMGs_ChIP_MEF2C_rep4_CTN_l20240905_TGAGGCGC_GGAGACCA_S12_L007_R1_001.fastq.gz
human_KO2H10_iMGs_ChIP_MEF2C_rep4_CTN_l20240905_TGAGGCGC_GGAGACCA_S12_L007_R2_001.fastq.gz
H3K27ac_Control_42N_rep1.txt
H3K27ac_Control_42N_rep2.txt
H3K27ac_Control_71N_rep1.txt
H3K27ac_Control_71N_rep2.txt
H3K27ac_Het_237_rep1.txt
H3K27ac_Het_237_rep2.txt
H3K27ac_Het_2H11_rep1.txt
H3K27ac_Het_2H11_rep2.txt
H3K27ac_KO_2H10_rep1.txt
H3K27ac_KO_2H10_rep2.txt
H3K27ac_KO_IG4_rep1.txt
H3K27ac_KO_IG4_rep2.txt
MEF2C_ChIP_Control_42N_CN.txt
MEF2C_ChIP_Control_D4N_CN.txt
MEF2C_ChIP_KO_1F37_CN.txt
MEF2C_ChIP_KO_2H10_CN.txt
MEF2C_ChIP_Het_237_CN.txt

MEF2C_ChIP_Het_2H11_CN.txt
human_iMG_ATAC_237_MEF2CHet_rep1_CZH_l20210409_TTGACCCT.ucsc.bedGraph.gz
human_iMG_ATAC_237_MEF2CHet_rep2_CZH_l20210409_CACGTCGA.ucsc.bedGraph.gz
human_iMG_ATAC_2H10_MEF2CKO_rep1_CZH_l20210409_AAGAGGCA.ucsc.bedGraph.gz
human_iMG_ATAC_2H10_MEF2CKO_rep2_CZH_l20210409_AGGTTGGG.ucsc.bedGraph.gz
human_iMG_ATAC_2H11_MEF2CHet_rep1_CZH_l20210409_CCTCCACG.ucsc.bedGraph.gz
human_iMG_ATAC_2H11_MEF2CHet_rep2_CZH_l20210409_AAGGAACG.ucsc.bedGraph.gz
human_iMG_ATAC_42N_MEF2CCtrl_rep1_CZH_l20210409_CGTACTAG.ucsc.bedGraph.gz
human_iMG_ATAC_42N_MEF2CCtrl_rep2_CZH_l20210409_TCCTGAGC.ucsc.bedGraph.gz
human_iMG_ATAC_71N_MEF2CCtrl_rep1_CZH_l20210409_GGACTCCT.ucsc.bedGraph.gz
human_iMG_ATAC_71N_MEF2CCtrl_rep2_CZH_l20210409_CTCTCTAC.ucsc.bedGraph.gz
human_iMG_ATAC_IG4_MEF2CKO_rep1_CZH_l20210409_GTGTGGTG.ucsc.bedGraph.gz
human_iMG_ATAC_IG4_MEF2CKO_rep2_CZH_l20210409_TGGGTTTC.ucsc.bedGraph.gz
human_iMG_ChIP_H3K27ac_237_Het_rep1_CZH_s20210820_TGCTGAGG_CGCAACGT.ucsc.bedGraph.gz
human_iMG_ChIP_H3K27ac_237_Het_rep2_CZH_s20211008_TTATCAAC_CGAGTACG.ucsc.bedGraph.gz
human_iMG_ChIP_H3K27ac_2H10_KO_rep1_CZH_s20210820_GGATAATA_TGGTACTA.ucsc.bedGraph.gz
human_iMG_ChIP_H3K27ac_2H10_KO_rep2_CZH_s20210820_TGAAGAAT_GAAGACAG.ucsc.bedGraph.gz
human_iMG_ChIP_H3K27ac_2H11_Het_rep1_CZH_s20210820_ACCATTAA_GAACCGGT.ucsc.bedGraph.gz
human_iMG_ChIP_H3K27ac_2H11_Het_rep2_CZH_s20210820_TTAGGTTG_GACGATTG.ucsc.bedGraph.gz
human_iMG_ChIP_H3K27ac_42N_Control_rep1_CZH_s20210820_ACCGAGGT_CTCGTGTC.ucsc.bedGraph.gz
human_iMG_ChIP_H3K27ac_42N_Control_rep2_CZH_s20210820_ACCATATC_TACACGTT.ucsc.bedGraph.gz
human_iMG_ChIP_H3K27ac_71N_Control_rep1_CZH_s20211008_CTCTTCGT_CCGTCTAA.ucsc.bedGraph.gz
human_iMG_ChIP_H3K27ac_71N_Control_rep2_CZH_s20210820_GTTCTACT_GTAGCCAC.ucsc.bedGraph.gz
human_iMG_ChIP_H3K27ac_IG4_KO_rep1_CZH_s20210820_ACGTCCAT_GTGGCGAA.ucsc.bedGraph.gz
human_iMG_ChIP_H3K27ac_IG4_KO_rep2_CZH_s20210820_ACTCTACG_CCAGCTGA.ucsc.bedGraph.gz
human_iMG_ChIP_input_237_Het_rep1_CZH_s20210820_CACAGTAA_CACTAGAG.ucsc.bedGraph.gz
human_iMG_ChIP_input_237_Het_rep2_CZH_s20211008_CAGACCAC_GCTTCGGC.ucsc.bedGraph.gz
human_iMG_ChIP_input_2H10_KO_rep1_CZH_s20210820_CGATTATC_GAGTTGAT.ucsc.bedGraph.gz
human_iMG_ChIP_input_2H10_KO_rep2_CZH_s20210820_CAATGATG_CACGGACG.ucsc.bedGraph.gz
human_iMG_ChIP_input_2H11_Het_rep1_CZH_s20210820_GAATGCTC_GTCTAACA.ucsc.bedGraph.gz
human_iMG_ChIP_input_2H11_Het_rep2_CZH_s20210820_TGAGTCAG_CCGAACTT.ucsc.bedGraph.gz
human_iMG_ChIP_input_42N_Control_rep1_CZH_s20210820_CGTATTGG_GGTATCTT.ucsc.bedGraph.gz
human_iMG_ChIP_input_42N_Control_rep2_CZH_s20210820_GAACTTAT_AGATACGC.ucsc.bedGraph.gz
human_iMG_ChIP_input_71N_Control_rep1_CZH_s20210820_CGAAGGAC_GTTGACGC.ucsc.bedGraph.gz
human_iMG_ChIP_input_71N_Control_rep2_CZH_s20210820_GATCAACA_CTATGTTA.ucsc.bedGraph.gz
human_iMG_ChIP_input_IG4_KO_rep1_CZH_s20210820_CGGTGGTA_GCCTAGTA.ucsc.bedGraph.gz
human_iMG_ChIP_input_IG4_KO_rep2_CZH_s20210820_CATGATGA_GTTAGAGG.ucsc.bedGraph.gz
MEF2C_ChIP_Control_42N_CN.ucsc.bedGraph.gz
MEF2C_ChIP_Control_D4N_CN.ucsc.bedGraph.gz
MEF2C_ChIP_Het_237_CN.ucsc.bedGraph.gz
MEF2C_ChIP_Het_2H11_CN.ucsc.bedGraph.gz
MEF2C_ChIP_KO_1F37_CN.ucsc.bedGraph.gz
MEF2C_ChIP_KO_2H10_CN.ucsc.bedGraph.gz

Genome browser session (e.g. UCSC)

https://genome.ucsc.edu/s/awarden/MEF2C_combined

Methodology

Replicates

Each MEF2C ChIP seq experiment includes 2 samples per genotype. Each H3K27ac ChIPseq experiment includes 4 samples per genotype. Each ATAC ChIP seq experiment includes 2 samples per genotype

Sequencing depth

RNA-seq: 20 million reads per sample
ATAC-seq: 25 million reads per sample
H3K27ac ChIP-seq: 25 million reads per sample
MEF2C ChIP-seq: 25 million reads per sample

Antibodies

MEF2C ab = Cell signaling tech #5030
H3K27ac ab = Active Motif #39685

Peak calling parameters

Fastq reads were mapped to hg38 genome build with default parameters. Aligned reads were saved in sam files and subsequently converted to tag directories with HOMER. Standard Homer peak calling: findPeaks using the parameters: '-L 0 -C 0 -fdr 0.9 -minDist 200 -size 200'.

Data quality

ChIP-seq with replicates were filtered using Irreproducible Discovery Rate (IDR., Peaks with IDR>=0.05 were filtered).

Software

HOMER

Flow Cytometry

Plots

Confirm that:

☒ The axis labels state the marker and fluorochrome used (e.g. CD4-FITC).

☒ The axis scales are clearly visible. Include numbers along axes only for bottom left plot of group (a 'group' is an analysis of identical markers).

☒ All plots are contour plots with outliers or pseudocolor plots.

☒ A numerical value for number of cells or percentage (with statistics) is provided.

Methodology

Sample preparation	iPSC derived microglia
Instrument	BD Influx
Software	Flow-Jo software v10
Cell population abundance	Cells were analyzed but not sorted by flow cytometry.
Gating strategy	Supplementary figure 2

☒ Tick this box to confirm that a figure exemplifying the gating strategy is provided in the Supplementary Information.
