# Supplementary figures and images for: Transcriptional and epigenetic targets of MEF2C in human microglia contribute to cellular functions related to autism risk and age-related disease
Source: Nat Immunol. 2025 Oct 22;26(11):1989–2003. doi: 10.1038/s41590-025-02299-0 (PMC12571900; doi:10.1038/s41590-025-02299-0)

Preview window displaying pre-stained ladder

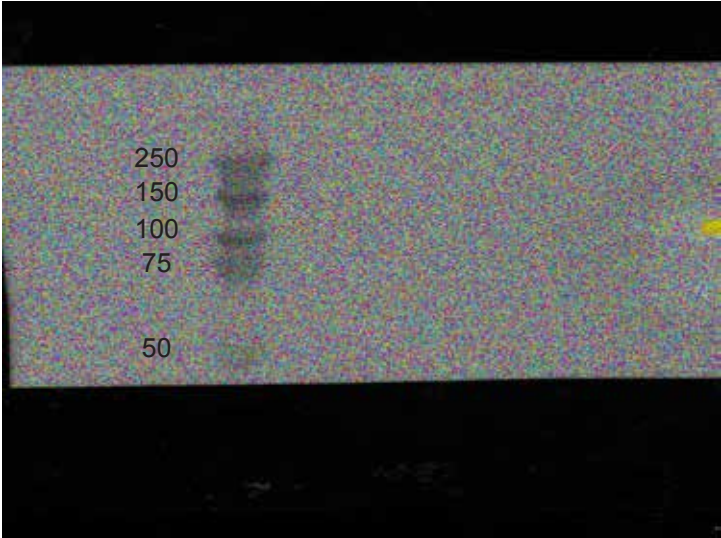

Chemiluminescence

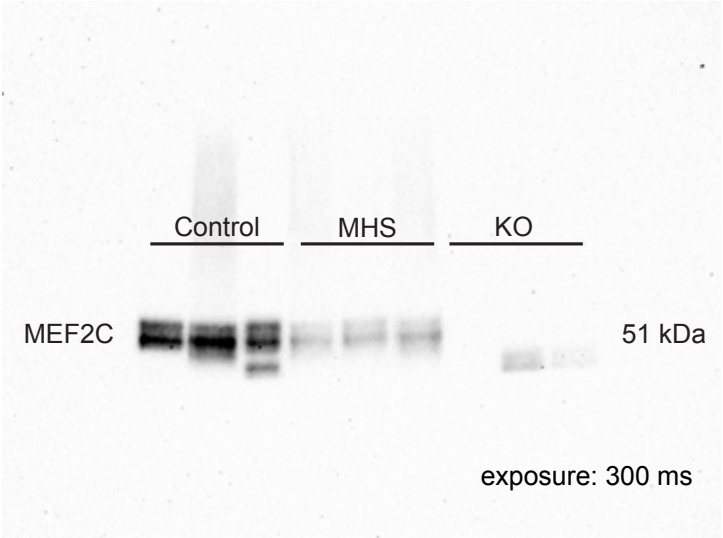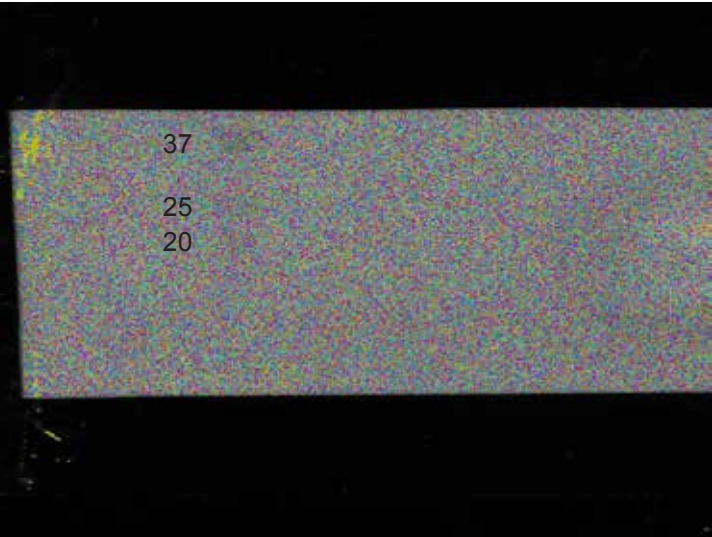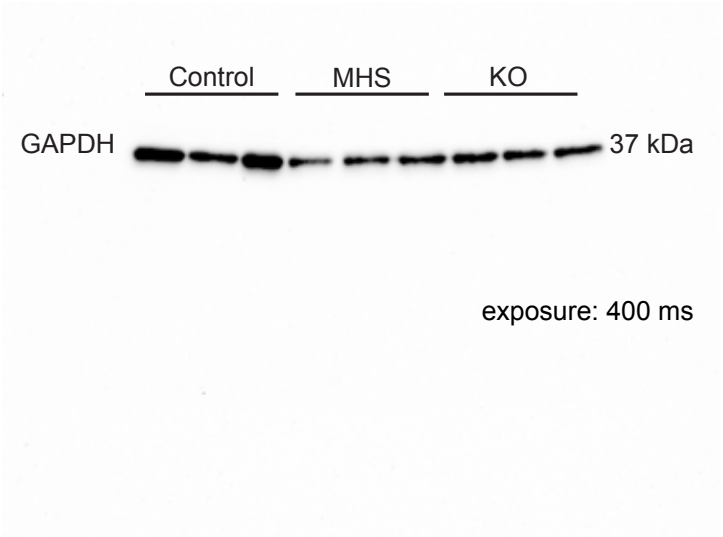

Supplement: Supplementary file 4 — Unprocessed western blots for Fig. 1f. [file 41590_2025_2299_MOESM4_ESM.pdf]

## RedAlert: Total Protein

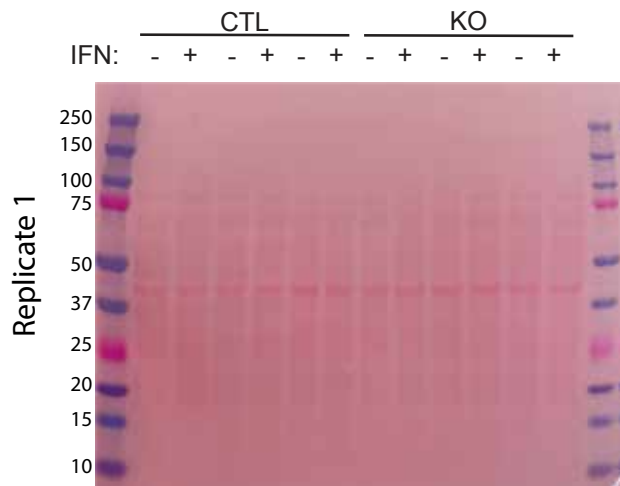

## Immunoblot (fluorescence)

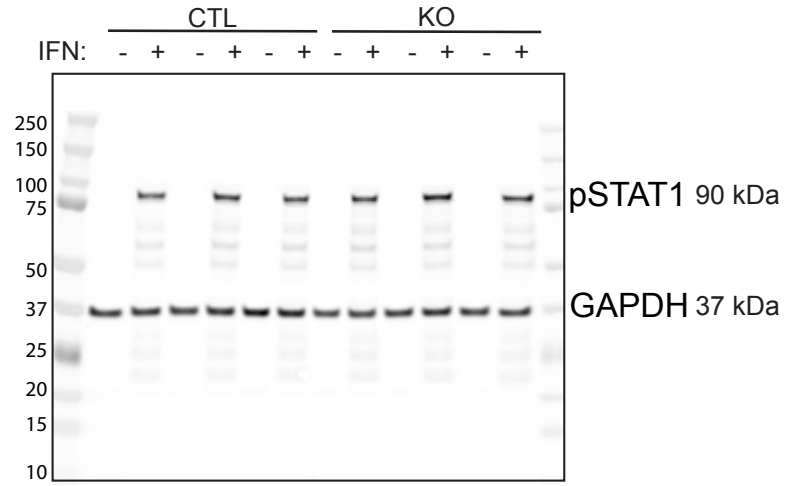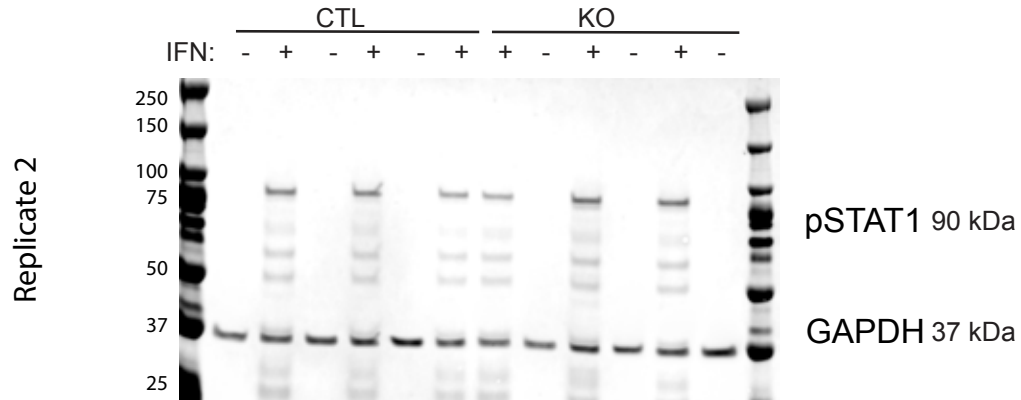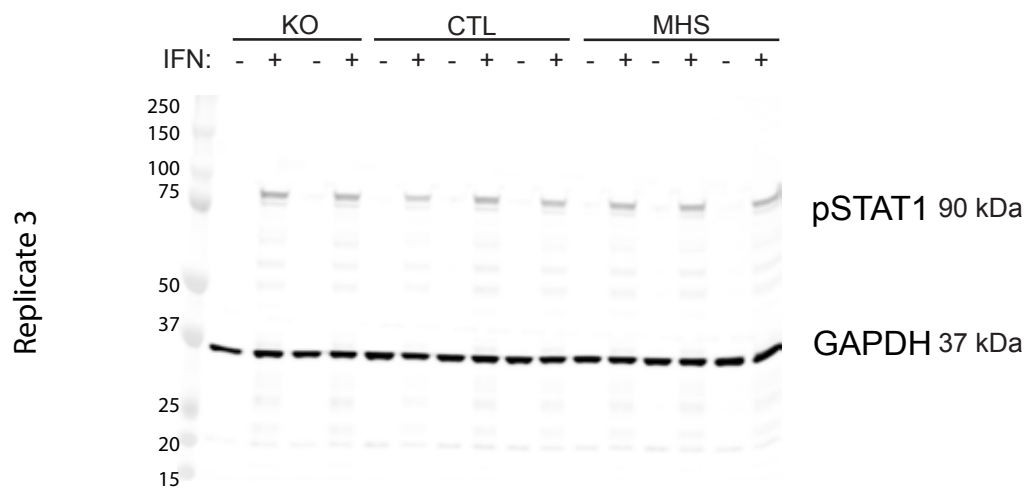

Supplement: Supplementary file 6 — Unprocessed western blots for Fig. 3j. [file 41590_2025_2299_MOESM6_ESM.pdf]
